# Detection of Patient Subgroups with Differential Expression in Omics Data: A Comprehensive Comparison of Univariate Measures

**DOI:** 10.1371/journal.pone.0079380

**Published:** 2013-11-22

**Authors:** Maike Ahrens, Michael Turewicz, Swaantje Casjens, Caroline May, Beate Pesch, Christian Stephan, Dirk Woitalla, Ralf Gold, Thomas Brüning, Helmut E. Meyer, Jörg Rahnenführer, Martin Eisenacher

**Affiliations:** 1 Medizinisches Proteom-Center, Ruhr-University Bochum, Bochum, Germany; 2 Department of Statistics, TU Dortmund University, Dortmund, Germany; 3 Institute for Prevention and Occupational Medicine of the German Social Accident Insurance, Institute of the Ruhr-Universität Bochum (IPA), Bochum, Germany; 4 Neurological Clinic, St. Josef-Hospital, Ruhr-University Bochum, Bochum, Germany; University of California, Los Angeles, United States of America

## Abstract

Detection of yet unknown subgroups showing differential gene or protein expression is a frequent goal in the analysis of modern molecular data. Applications range from cancer biology over developmental biology to toxicology. Often a control and an experimental group are compared, and subgroups can be characterized by differential expression for only a subgroup-specific set of genes or proteins. Finding such genes and corresponding patient subgroups can help in understanding pathological pathways, diagnosis and defining drug targets. The size of the subgroup and the type of differential expression determine the optimal strategy for subgroup identification. To date, commonly used software packages hardly provide statistical tests and methods for the detection of such subgroups. Different univariate methods for subgroup detection are characterized and compared, both on simulated and on real data. We present an advanced design for simulation studies: Data is simulated under different distributional assumptions for the expression of the subgroup, and performance results are compared against theoretical upper bounds. For each distribution, different degrees of deviation from the majority of observations are considered for the subgroup. We evaluate classical approaches as well as various new suggestions in the context of omics data, including *outlier sum*, *PADGE*, and *kurtosis*. We also propose the new *FisherSum* score. ROC curve analysis and AUC values are used to quantify the ability of the methods to distinguish between genes or proteins with and without certain subgroup patterns. In general, FisherSum for small subgroups and 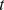-test for large subgroups achieve best results. We apply each method to a case-control study on Parkinson's disease and underline the biological benefit of the new method.

## Introduction

Subgroup detection is a common goal in many analyses of modern omics data. For gene and protein expression data, the most frequent research task is the comparison of a control group and an experimental group, or of healthy and diseased subjects. Parametric methods, e.g. based on Student's 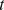-test or moderated 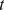-test statistics, as well as non-parametric methods, e.g. based on permutations as in the Wilcoxon test statistic, are commonly used. However, in many cases the underlying assumption of these methods, a homogenous experimental group, is not justified. This is especially true for cancer biology where even a clearly defined cancer type can be associated with a subgroup structure due to inherent biological heterogeneity. It has been shown that for several cancer types certain oncogenes can cause heterogeneous expression patterns, e.g. in breast cancer [1], lung cancer [2], and prostate cancer [3]. In such cases the disease group might be decomposed into subgroups that can be characterized by differential expression for different sets of genes or proteins.

For some cancer types, such patient subgroups are known to some extent and can be explained by different molecular subtypes of the cancer, or by clinical variables like tumor grade or stage. Moreover, the underlying aim for the ongoing development of subgroup detection methods is the identification of yet unknown subgroups that are of clinical relevance, for example since group membership correlates with progression or therapy response. Results from subgroup detection analysis might be highly relevant to personalized medicine, e.g. for the development of new drug targets. Therefore, a large amount of data is collected and searched for variables with a distinct expression pattern: Preferably, only the (yet unknown but) relevant patient subgroup shows e.g. higher expression levels in particular variables, whereas the expression between healthy controls and other diseased patients does not differ. Thus, instead of identifying biomarkers that are able to distinguish all observations in the experimental group from controls, often the focus is on the detection of single features, i.e. genes or proteins, that characterize disease stages or disease subtypes. In this case, subgroup detection approaches are based on univariate methods.

In many software packages for the analysis of omics data only basic measures and tests for differential expression are available, typically including Student's 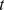-test. This is the optimal test for the detection of location differences between two groups of samples if all observations per group are realizations of random variables with the same normal distribution. However, depending on the aim of the study exactly those variables that do not meet the assumption of identical distributions are the most interesting ones.

For that reason, many univariate methods for subgroup detection have been proposed in the literature, especially in the context of omics data analysis. Depending on the interest of the researcher these methods can be used as stand-alone methods for univariate analyses or for dimensionality reduction before the application of multivariate methods [4]. introduced COPA, a method for *cancer outlier profile analysis*, which is shown to be more powerful than the 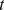-test, in case of a small number of up-regulated values. An important aspect of COPA is the search for pairs of genes with mutually exclusive outlier samples. For the above mentioned reasons, we focus on univariate methods and do not consider COPA in our work [5]. proposed a two-step procedure called *profile analysis using clustering and kurtosis* (PACK), which consists of a preselection step to identify variables of interest and the subsequent computation of the kurtosis to characterize the subgroup pattern and rank the variables according to their importance [6]. proposed the *outlier sum* (OS), in which first location and variance are determined with robust measures and then values of extreme observations are summed up to an overall score [7]. presents a refinement, the *outlier robust* 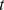 *-statistic* (ORT), in which location and variability are estimated only from observations of the control group [8]. presented the *percentile analysis for differential gene expression* (PADGE), a strategy that makes use of existing statistical tests (e.g. 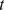-test, Wilcoxon test) and applies them iteratively to subsets of extreme observations, where the size of the subset is decreasing. Existing subgroups will cause a characteristic increase in the fold change when plotted against percentiles.

In previously published simulation studies, subgroup size is varied and for the subgroup of interest, a fixed distribution is considered, mostly a normal distribution with shift 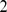 [9]. compared the performance of OS and Student's 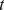-test and were the first to consider a more general, non-parametric shift alternative. However, a comprehensive comparison of all these approaches is still missing. The goal of this paper is to perform a profound comparison of basic tests commonly used in omics facilities with such tests that are specifically developed for outlier or subgroup detection. We provide guidance in which situation which method is best or at least competitive. We also include a new test strategy that is based on the simple idea of Fisher's exact test.

In addition to univariate statistical tests for differential analysis, many software solutions provide multivariate procedures such as principle component analysis (PCA) or hierarchical clustering. These methods are useful for visualization of the structure of the data and to detect global differences between groups of samples, and we highly recommend their use to obtain a first impression of the data. They facilitate the examination if the most relevant groups (such as healthy vs. diseased or treated vs. untreated) separate well or if problems with the sample material or preprocessing steps are likely. Thus, systematic errors that result from batch or lab biases may become apparent. Obviously, in case the PCA reveals any subgroups of samples in the disease group, these should be analyzed further. But unfortunately, in many applications, there are no subgroups that stand out in resulting biplots. This is due to the fact that small subgroups often have only a small impact on the overall variation in the data, especially if the subgroup only differs regarding a small proportion of the variables in the data set or if the difference in expression levels is small. This has been shown before, e.g. by [10], who have illustrated by means of real and simulated data, that this type of subgroup does not necessarily separate from the remainder of observations in ordinary biplots. Again, basic methods like PCA are not appropriate for the detection of small subgroups in single variables of larger data sets. Precisely since each variable is assessed independently at first, univariate approaches permit a different view on the data in comparison to common multivariate methods.

We present an innovative design for simulation studies in the context of subgroup detection. We focus on variables where observations in the reference group (controls or healthy subjects) and the majority of the disease group follow the same distribution and the remaining observations form exactly one subgroup within the disease group and show differential values. Note that the assumption of a single subgroup per variable does not imply that the disease group in general incorporates only one subgroup. In fact, it is quite possible that there are several subgroups that reflect e.g. different subtypes of the disease. More details on this issue can be found in the discussion. In our simulation, different assumptions about the differential subgroup size and the corresponding distribution are made. We investigate different types of location and scale shifts of the expression in the subgroup. In contrast to previous publications, we do not vary total sample size and the size of the subgroup alone, but also the type of the subgroup distribution as well as the degree of deviation of the subgroup. This means that different methods are compared over a wide range of alternatives in contrast to the common comparison at one or two alternatives, which may or may not reflect the general performance of the methods. Furthermore, the consideration of a case-control design allows for the consideration of variables that show non-disease-specific subgroups, which is an important factor when it comes to real data application. However, this has been ignored for the most part up to now. Another advantage of our approach is that we can calculate an upper bound for the performance, since in the controlled scenario of the simulation study the true distributions and therefore the theoretically optimal likelihood ratio is available. We also apply the considered tests to real proteomics data from a case-control study on Parkinson's disease and show that the new FisherSum method generates new biological insights.

The remainder of this work is organized as follows. The section *Methods* introduces all measures and tests that are taken into consideration for our simulation study, including the new method. We then describe the overall design of the *Simulation study for subgroup detection*, list the different parameter settings that were used with different distributional assumptions, elucidate our advanced study design, and explain the quality criterion we chose to compare the different methods. Afterwards, we present the *Results* of the simulation study and of the real data analysis. We close with a *Discussion* and an outlook.

## Methods

### Tests and Scores in comparison

In this section, we introduce all methods for subgroup detection that are compared in the simulation study. Mostly up-regulation is regarded as the preferred direction for biomarkers. Thus we consider, w.l.o.g., the one-sided case of up-regulated subgroups, with corresponding adaptations in tests and scores where possible, e.g. the one-sided instead of the two-sided 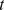-test. If desired, the methods are also easily applicable to bidirectional regulations.

As standard method, we include Student's 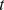-test, a good choice for global shifts. In the next section, we first introduce four methods for subgroup detection that were developed in the context of omics data, namely OS [6], ORT [7], PADGE [8] and kurtosis as central part of PACK [5]. Corresponding R code can be found in File S1. Additionally, we include Bartlett's test for homoscedasticity as possible alternative. All these methods are univariate and corresponding scores and 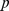-values respectively are computed separately for each variable in the data set, independently from observations in other variables.

We then introduce a new score called *FisherSum* that combines ideas from the *Minimum M* score introduced by [11] and the outlier sum. Finally, we describe the calculation of an upper theoretical bound on the performance of the tests.

### Previously presented methods for subgroup detection

The test statistic OS sums up expression values that are regarded as outliers according to a robust definition. First step is the robust standardization of the expression values per gene by dividing the median-centered values by the MAD (median absolute deviation). Values from the disease group 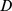 that exceed a threshold 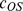, which is the sum of the 75 percent quantile 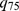 and the interquartile range 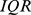 per gene, are defined as outliers and added up to the OS, i.e.
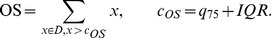
Large values of OS can result from a single outlier much greater than 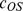 or from an outlier subgroup above the threshold.

The outlier definition that is used for ORT is presented as an improved version of OS. The authors argue that in the calculation of OS, both centering and scaling factors per gene are derived from all observations, i.e. from both groups, which leads to overestimation of variance and location in case of present subgroups. In contrast to OS, the threshold for outlier definition 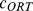 is derived based on the observations in the control group only. Let 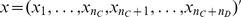 denote the observed expression values for a single variable. The median of the controls 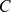, 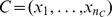, is denoted by 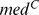, and 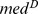 is defined analogously for the disease group 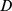, 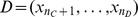. 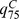 and 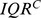 denote the 75 percent quantile and the interquartile range, respectively, of the expression values in 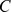. The set 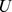 of *outlier disease samples* contains the observations in 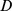 that exceed the threshold 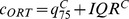. Then the test statistic can be written as
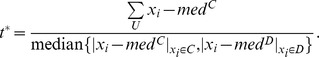
The authors showed that ORT outperforms OS in several situations.

PADGE is a more complicated method that conducts statistical tests on a set of subsets of two groups. First, the user chooses a series 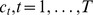, e.g. 80, 85 and 90 percent, which are used to define subsets in the controls 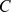 and diseased patients 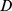, respectively, as follows:
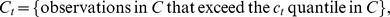


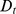
 analogously. The chosen statistical test, e.g. Student's 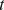-test or Wilcoxon's rank-sum test, is derived on each of the 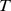 resulting pairs of subsets, and the corresponding 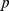-values are corrected for multiple percentiles afterwards. Additionally, the expression ratios 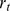 between the subsets are calculated. Assuming that the control group is homogeneous and the disease group contains an up-regulated subgroup, the difference in location will become more apparent with increasing quantiles. The authors propose a summary score for candidate ranking that considers the corrected 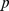-values 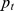 as well as the (relative) expression ratio between the (subgroups of) samples:
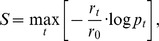
where 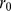 is the expression ratio between the groups when all observations are included. Thus, the term 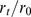 describes the relative variability of overexpression in the disease group. As the exact computational steps are not described down to the last detail in the corresponding publication, we use a PADGE-like score in our simulation study.

PACK allows the researcher to look for variables where the sample falls into major subdivisions as well as variables that show small outlier groups. The purpose of PACK's clustering step is the preselection of variables that are most likely to show heterogeneous expression. We skip the initial step and directly compute the kurtosis for each variable by using the R package e1071 [12]. This simpler one-step version is referred to as PAK in the manuscript.

We also included Bartlett's test for homoscedasticity (from the R package stats, [13]) in our study, because basically each variable that shows significant changes in variation between the two groups in comparison can be potentially interesting. The test assesses if two groups have equal variance, under the assumption of normality.

### FisherSum: a new method based on the idea of Fisher's exact test

The software *ProtoArray Prospector* (Life Technologies, Carlsbad, California, USA) provides a test statistic that is called Minimum M statistic. It is a rank-based method that aims at finding unknown subgroups of patients in two-group comparisons. Basically, the proposed procedure is equivalent to a Minimum Fisher's exact test, where the minimum is determined from a set of 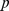-values. In turn, each observation is tested as possible cutpoint. Since we focus our attention on smaller subgroups, we do not adopt the method as it stands but instead use Fisher's exact test in a similar manner.

Generally, Fisher's exact test is used to analyze the statistical dependence of two binary variables. We investigate the dependence between the group membership (disease group 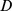 or controls 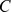) and the size of the observed values, i.e. whether the individual values exceed a particular cutoff 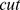. This corresponds to a contingency table as shown in Table 1.

**Table 1:** Contingency table to assess the dependence between the group membership and the size of the observed values.

	*D*	*C*	
>*cut*	*n*_11_	*n*_12_	*n*_1_.
≤*cut*	*n*_21_	*n*_22_	*n*_2_.
	*n_D_*	*n_C_*	

Here, 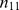 denotes the number of observations in 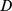 with expression values above the cutoff 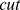. For our purpose, a reasonable cutoff is a quantile of the values in the disease group per variable. In the following, 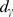 denotes the 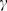 percent quantile of 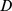, i.e. 10 percent of the values in 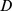 are larger than 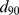. Under the null hypothesis that there is no difference between the groups, 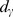 should be similar to 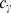, the corresponding 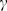 percent quantile of the controls, and one would expect that about 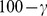 percent of the values in 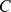 and in 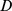, respectively, exceed 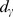. On the contrary, in case of a real e.g. 10 percent subgroup in 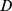 with increased values, no (or at least few) values in the control group should exceed the cutpoint 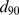.

For each variable in the data set, we choose the corresponding quantile 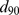 as cutpoint 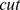. Our experience is that this cutoff yields good results on both real and simulated data compared to other quantiles. It does not attach too much importance to single outliers or very small groups of up-regulated observations, that are more likely to be false positives or hard to validate, and at the same time it works also well for subgroups larger than 10 percent. In the latter case, only the most extreme observations are taken into account, which corresponds to a smaller subgroup with larger deviation from the remainder of the group. Therefore, variables that show a global difference between the two groups will also yield a higher FisherSum score depending on the amount of shift. In principle, the 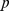-values of the corresponding Fisher test (which we call Fisher10, see File S1) serve the purpose to rank variables according to the potential existence of up-regulated subgroups, but deriving the Fisher tests for each variable of a high-throughput data set leads to large numbers of ties regarding the 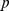-values, due to the discrete character of the test statistic. Besides, it can yield a number of false positive results. Thus, instead of directly using the 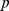-values, we propose a scoring method following the idea of OS. Basically, after centering the observations with the median of the controls, we sum up the values that correspond to 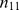, i.e. the values in 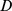 above the cutpoint, and subtract the sum of observations corresponding to 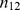. In more detail, both sums are adjusted using weights 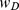 and 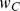 such that our test statistic FisherSum can be written as
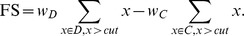
The subtraction represents a penalty for variables that show up-regulated subgroups in both control and disease group. We refer to this pattern as non-disease-specific subgroups. A natural choice for 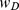 and 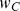 is 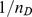 and 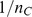, respectively, which becomes important in case of unbalanced designs or for the comparison of scores across studies with different sample sizes. If only a single study with balanced design is of interest, one can simply choose 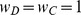. In general, the weights can also be used to adjust the magnitude of the penalty for a non-disease-specific pattern if desired by the researcher.

Depending on the study design, one might want to provide 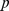-values in addition to the actual value of the FisherSum FS to control the type I error. As for methods like OS and ORT where the distribution of the test statistic is not expressible in closed form, 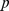-values can be obtained by estimating the distribution of FS under the null situation. For an explorative analysis and to generate hypotheses for future studies, it might be sufficient to rank the variables according to decreasing FS values, as it was analogously proposed for PACK [5].

We are well aware that Fisher's exact test assumes fixed margins instead of fixed entries in certain cells. In our simulation we compared these two methods, computing the quantile 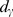 from the disease group only, which results in stochastic margins with fixed cell values as well as the computation of the 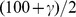 quantile from the pooled data. The first approach outperformed the theoretically more accurate second version in the simulation (data not shown) and was chosen as the new Fisher type algorithm. Due to our focus on up-regulated subgroups in 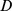, we apply a slight modification where we set negative values to zero.

## Simulation Study for Subgroup Detection

A commonly assumed distribution for expression values is the normal distribution 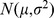 which is meant to represent the noise in real data. Usually, in simulation studies the values of a subgroup are then drawn from 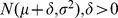, most often with 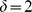. While we generally agree with the assumption of normality for the majority of observations, we recommend to reconsider the assumptions regarding the dysregulated subgroup. We are convinced that in the biological context, a shift in means usually goes along with an increased variance, which makes truly existing subgroups even harder to detect. To our knowledge, we are the first to evaluate the effect of simultaneously increased mean and variance in the patient subgroup together with the yet ignored non-disease-specific patterns. Note that this simultaneous increase of mean and variance is due to biological reasons and relates to the values corresponding to the patient subgroup in comparison to the other observations. This biological effect appears for variables regardless of their location, i.e. for variables with high as well as with lower values. The increased variance would also apply to subgroups that are down-regulated in comparison to the remainder of the observations. It is not to be confused with the general effect of variances depending on the mean, which should be taken care of by appropriate data normalization: a log-like scale instead of the original scale, e.g. log-intensities in gene expression analysis or in fluorescence-based techniques like protein microarrays. Without proper normalization and transformation, larger values tend to have larger variances due to technical reasons. For variables with values in the lower to medium range the effects may be negligible. On the other hand, for variables with large absolute values and a truly present patient subgroup there is more variation to the values themselves and the resulting computed scores.

In summary, the aims and special features of our study are as follows. We assess the effect of different types of distributions for the observations in a patient subgroup. For each distribution type, a range of parameters is considered, which corresponds to an increasing difference between the subgroup and the remainder of the observations. This permits a comprehensive comparison of the different methods. Additionally, our extended definition of the null situation allows the assessment of the robustness of each method against false positive results, where both control and disease group show up-regulated subgroups. This indicates a non-disease-specific subgroup, that might be due to unknown confounders or to single values that are extreme by chance, and that is not considered relevant for the characterization of disease subgroups.

### Design of the simulation study

Before going into details of the simulation, we first establish some notation. Consider a two group comparison between a control group 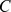 and a disease group 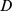, both of sample size 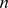. Let 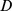 contain a patient subgroup of size 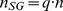 with 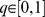, and w.l.o.g. assume 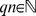. In clinical practice, the focus often is on smaller subgroups with 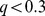. However, for statistical considerations and for the sake of completeness, we also include large values of 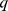 up to 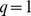, which corresponds to the whole disease group. Let 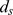 denote the underlying distribution for the patient subgroup in scenario 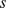, 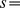
I, II, III, and let 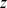 denote the degree of deviation from the remainder of observations which follow the standard normal distribution 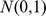. Then the distribution 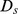 in the complete disease group 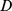 is modelled as follows as in equation (1) with the subgroup distribution 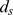 as listed in Table 2.
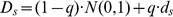
(1)


**Table 2:** Different subgroup distributions considered in the simulation studies.

*s*	*z*	*d_s_*	Increase in
I	*δ*>0	*N*(*δ*,1)	mean
II	*b*>0	*N*(0,1)+*U*[0,*b*]	mean and variance
III	*σ*>1	*N*(0,*σ* ^2^)	variance

Distribution 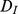 reflects the assumption that is made most commonly in publications to date. 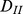 is an example for the combination of simultaneously increased mean and variance, where 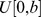 denotes the uniform distribution on the interval 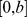. We have chosen a one-parameter alternative hypothesis, due to comparability with the other cases. Even though this distribution is not a typical choice, the resulting distributional patterns look much more like real data distributions in comparison to 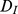. Regardless of the actual distribution 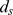 of the subgroup (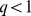), the variance in the disease group is increased compared to the variance in the control group. Moreover, it seems quite reasonable from a biological point of view, that levels of e.g. disease related proteins have a wider distribution in 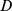. In contrast to scenarios I and II, there is no shift in the expected means between the groups in scenario III, and the dysregulation is two-sided. We include this different scenario to point out that there are even more possibly interesting alternatives. It is interesting to see how methods mainly optimized for scenario I perform in scenario III. For fixed sample and subgroup sizes, each of the scenarios can be parameterized by a single parameter which will be referred to as 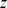. Depending on the scenario, 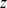 equals 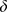, 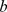, or 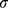, respectively.

To our knowledge, we are the first to consider different distributional assumptions as well as varying subgroup and sample sizes. Previous publications mostly varied the proportion of the subgroup for a fixed shift in means. Our simulation study includes all combinations of sample sizes 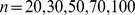 per group and subgroup proportions 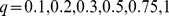. Results not shown in this paper can be found in File S1. Depending on the omics technique, sample sizes are sometimes very low (below 10). Even so, whenever the aim of a study is the characterization of unknown subgroups, the data sets should contain significantly larger numbers of samples in order to achieve reliable and meaningful results. Hence, our study only includes sample sizes starting from 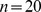.

In our setting, we want to distinguish variables that show the desired up-regulation only in a subgroup of the disease group 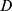 from variables with a so-called null situation. Let 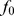 and 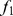 denote the density of a variable without and with subgroup, respectively. Variables with disease-specific subgroup pattern (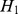) have densities 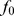 and 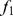 in control and disease group, respectively, whereas the other variables have the same densities in 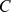 and 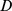. In case of no subgroups at all, 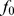 holds true for 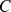 and 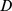 and if subgroups are present in both groups, all observations follow 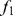 (according to scenario I, II, or III). These two null situations are called 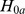 and 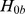, respectively. By expanding the definition of the null situation, we are able to assess the robustness of the different methods against non-disease-specific patterns (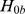). Results of simulation study and real data analysis will demonstrate this advantage of our study design over the commonly used simpler version. For reasons of comparability with previous studies, we conduct our comparison study both with the simple null situation 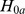 only and with the combination of the two null distributions 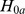 and 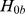. Let 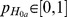 be the proportion of variables from 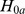 among the null situations. For our simulations we chose 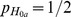 in order to evaluate the impact of the new null situation on the different methods. Per parameter setting we simulated 1000 variables in total and obtain and obtain a data matrix that contains variables from 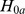, 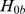 and 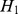 with different frequencies which are given in Table 3.

**Table 3:** Structure of the simulated data matrix.

	C	D	# Variables	Distributional Pattern
*H*_0*a*_	*f*_0_	*f*_0_	250	no subgroups at all
*H*_0*b*_	*f*_1_	*f*_1_	250	subgroups in *C* and *D*
*H*_1_	*f*_0_	*f*_1_	500	subgroup only in *D*

We compare the different methods by means of a quality criterion that is defined analogously to the AUC (area under the receiver operating characteristics curve). In the clinical context, the AUC is mostly used to assess the performance of a binary classifier. For example, a binary response such as healthy vs. diseased is predicted using a continuous variables like gene expression values or clinical parameters. In our study, the presence of the required distributional pattern for each variable is interpreted as binary response which is predicted with 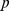-values or scores, respectively. In particular, we generate a data set of size 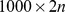 for every parameter combination according to the described pattern. This represents 1000 variables with 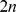 observations each (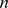 for 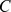 and 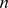 for 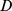), where the first 500 are drawn from a null situation and the last 500 from the respective alternative. The response vector *truth* thus has the form *truth* 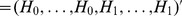. Then, the AUC is derived for each method separately by predicting this truth with the corresponding vector of *p*-values and scores, respectively. Sensitivity corresponds to the probability of correctly identifying a variable with a present subgroup only in 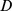, whereas specificity means that variables with identical distributions for both groups 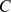 and 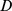 are classified correctly. We utilize the R package pROC [14].

Note that the application of AUC does not require the specification of a threshold based on which the single variables are classified as 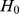 and 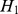, respectively. The choice of this cutoff depends on the study aim, where the focus may be either on specificity or on sensitivity, in other words on minimizing type I and type II error, respectively. Depending on the subsequent steps of the analysis one might want to start with a larger set of potentially interesting variables. To provide generally applicable results, we chose the AUC as quality criterion, as it allows the simultaneous assessment of sensitivity and specificity of each test method. The AUC scale has been used before, see e.g. [15]. Most of previous work on subgroup detection methods used *true positive vs. false positive* plots to illustrate results and compare different methods with respect to type I error. This is a convenient presentation only if the number of considered alternatives is small. As our focus is on the performance of the methods across a large range of alternatives (e.g. depending on the degree 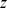 of deviation from the null hypothesis), this kind of presentation is less appropriate than a comparison on the AUC scale.

### Assessing upper bounds for performance using the likelihood ratio

In our simulations, we are not only able to compare different tests and scores regarding their performance, but we can also calculate the theoretically best performance achieved by the likelihood ratio (LR). It can reveal for which scenario or parameter range an improvement of the current methods would be most worthwhile. The LR is derived as ratio of 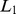, the likelihood based on the desired distributional pattern, and 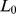, the likelihood assuming a null situation. According to the notation introduced above, LR can be derived as
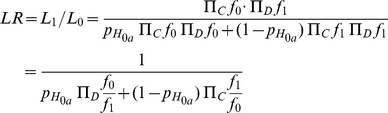
Note that LR must outperform the other methods in all our simulations, but it is not applicable to real data in this exact manner, since true subgroup size and subgroup distribution are not known for real data scenarios. However, in the simulation study the densities 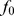 and 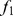 are defined by equation (1) and with all parameters fixed, the densities of the mixed distributions can be simulated easily. The parameter 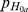, that characterizes the composition of the null situation, is chosen by the researcher as well. Altogether, the derivation of LR is straight forward in the simulation study.

## Results

We first present the results of an extensive simulation study. Then, a real data analysis is provided for proteomics measurements from a study on Parkinson's disease. This study shows that interesting patient subgroups and corresponding variables can be detected with the appropriate tests.

### Simulation study

**Figure 1:**
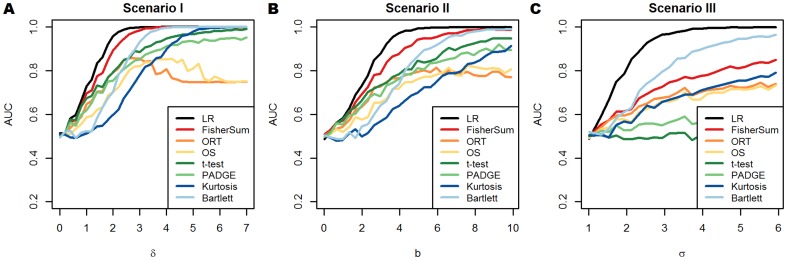
Comparison of different methods by means of AUC values. Deviation 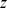 from 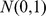 is given on x-axis, AUC value for distinguishing variables with and without the desired subgroup pattern on y-axis, according to simulation scenario I (mean increase, panel A), scenario II (mean and variance increase, panel B) and scenario III (variance increase, panel B), with group sizes 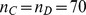 and true subgroup proportion 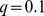. Plots correspond to 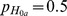, see text for more details. Colors of lines correspond to upper theoretical bound (black, LR) and seven tests and scores (other colors, see legend).

We illustrate the results of the simulation study in two steps. First, we consider a specific combination of sample size 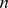 and subgroup proportion 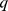 under various distributional assumptions, see Figure 1. We discuss the case of a 10 percent subgroup in a 70 versus 70 comparison, corresponding to the real data set that is analyzed afterwards. Our focus is on the advanced simulation design that incorporates two patterns for the null situation. Second, we describe the influence of variations in the parameters 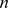 and 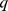, when the scenario is fixed (see File S1, section 1.1 for composite and section 1.2 for the simple null situation).

**Figure 2:**
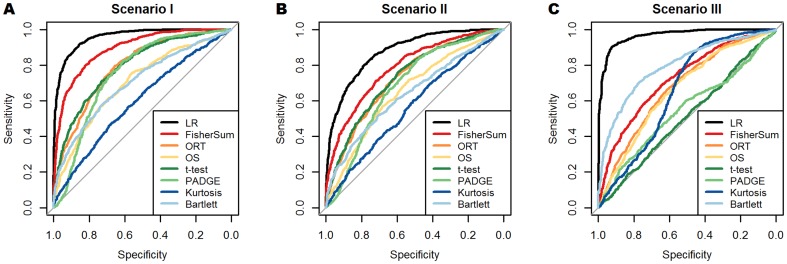
Comparison of different methods by means of ROC curves. Three panels correspond to panels in Figure 1, i.e. scenarios I, II, III with 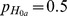 from left to right, see text for more details. The curves correspond to the following 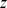 values: 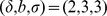. Colors of lines correspond to upper theoretical bound (black, LR) and seven tests and scores (other colors, see legend).

The following results correspond to the simulation design with the composite null situation and 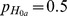. In Figure 1, each scenario is summarized by a single plot that depicts the AUC of the different methods depending on the degree 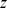 of deviation of the subgroup's distribution from 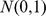. Thus, for each point 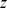 the methods could be compared in more detail regarding false positive and false negative rates by comparing the corresponding ROC curves (see also Figure 2). Again, we focus on the dependency of AUC values from 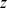, to which to date little attention has been paid. As can be seen from Figure 1, our new method FisherSum outperforms the other methods considerably in the case of up-regulated subgroups (scenarios I and II). The next best methods are 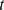-test and Bartlett's test, where the former one attains higher AUC values for smaller deviations while the latter one performs second best for larger values of 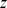. Note that AUC values for methods that do not check for outlier observations in the control group, namely OS and ORT, do not necessarily converge to 1. Overall, the performance of all methods is quite similar between scenario I and II and the order of the tests is basically the same.

The essential difference of scenario III compared to scenarios I and II is that the deregulation is bidirectional and does not cause a shift in the theoretical mean. Hence, AUC values from the 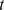-test are close to 0.5, and PADGE works only slightly better. From Figure 1 we also see that the gap between the optimal LR and the best method is much larger in this scenario, i.e. other tests are required for this kind of subgroup pattern. Except for very small deviations, Bartlett's test for homoscedasticity performs best. FisherSum is second best and distinctly better than the group of OS, ORT and kurtosis. Basically, FisherSum considers variables in scenario III as variables with an up-regulated subgroup of size 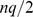.

The most interesting numbers regarding the parameter combination 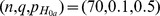 are summarized in Table 4.

**Table 4:** Comparison of methods for subgroup detection with respect to AUC values, for group size 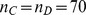 and subgroup size 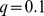, 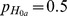, corresponding to Figure 1.

Criterion	AUC(LR)≥0.95			AUC(Method) = 0.95		
Scenario	I	II	III	I	II	III
LR (optimum)	0.96	0.95	0.96	2.0	3.63	2.93
FisherSum	**0.89**	**0.87**	0.72	**2.6**	**5.28**	>6
ORT	0.79	0.77	0.68	∞	∞	∞
OS	0.70	0.72	0.66	∞	∞	∞
*t*-test	0.79	0.77	0.49	4.2	9.24	∞
PADGE	0.75	0.74	0.55	6.6	>10	∞
Kurtosis	0.60	0.62	0.66	4.6	>10	>15
Bartlett	0.70	0.72	**0.80**	3.2	6.60	**5.33**

Left three columns contain AUC values when tests are compared at 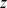 value with AUC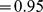 for LR. Right three columns correspond to 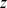 values with AUC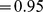 for each test. Best values plotted in bold per simulation scenario.

To receive an impression how the type I errors of the different methods compare, we present a generic set of ROC curves for each scenario and the combination 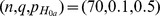 in Figure 2. Each panel in Figure 2 corresponds to one single value of 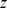 in one of the three panels in Figure 1. Since we want to summarize the method comparison across a range of alternatives, we prefer to present the results on the AUC scale.

For other combinations of group size 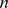 and subgroup proportion 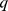 similar statements hold true. We summarize the findings in the next paragraphs. Corresponding plots can be found in File S1, section 1.1. For small to moderate subgroup sizes up to about 20–30 percent the new method FisherSum outperforms the existing methods in scenario I and II for all considered sample sizes in the middle range of deviations. For large subgroups of at least 50 percent, in scenarios I and II, the 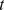-test performs best, followed by FisherSum, ORT and PADGE which yield quite similar results. For small deviations, these scores achieve values distinctly above 0.5, whereas kurtosis, OS, and Bartlett's test perform worse.

In case of a composite null situation with 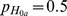, simulation results point out the major drawback of OS and ORT. For moderate subgroup sizes around 20 percent, they seem to work well for small deviations but then AUC values decrease, with an asymptotic value of 0.75 (in general 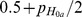). Apart from the fact that AUC values of kurtosis converge to 1 for small subgroups, it appears that generally other methods should be favored.

We point out that the kurtosis allows for the detection of two kinds of alternatives. On the one hand, large positive values indicate the existence of smaller subgroups, which we are interested in. On the other hand, if the size of the subgroup is about 50 percent, then large negative values are observed. As we analyze positive kurtosis as measure for subgroup detection, simulation results with larger subgroups show that the corresponding AUC values become much smaller than 0.5. Switching the group assignment in the calculation of the ROC curve would mirror the corresponding curves horizontally at 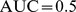.

In scenario III, in contrast to scenarios I and II, Bartlett's test performs best for virtually each combination of sample size and subgroup size. Only for smaller values of 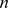, 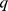, and 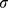 FisherSum performs slightly better. Noteworthy, even the gap between the the optimal LR and the Bartlett test for homoscedasticity is quite large.

Additionally, we conducted the whole simulation study again with 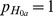, which corresponds to the commonly used design. In absence of non-disease-specific subgroups, OS and ORT perform much better and AUC values converge to 1 as desired. While there are some more differences concerning the other tests, the overall results are similar when it comes to best performances.

### Application to real data

In this section we assess the performance of the methods on a real data set. Whereas the task in the simulation study is to distinguish between the (composed) null situation and a single type of alternative, there is a mixture of several alternative patterns in the real data set, that may also include global shifts or single outliers for example. As can already be seen from the simulations, different methods detect different patterns well.

We analyze a subset of the data from the ParkCHIP project. The data have been described before, see [16], and are available at www.medizinisches-proteom-center.de/Ahrens_et_al. Samples from 72 patients with Parkinson's disease (PD) were compared to 72 samples from age and gender-matched healthy controls. The protein microarray used in this study provides about 9500 variables. As PD is known to be a heterogeneous disease (e.g. [17]), we expect that the methods detect interesting variables.

We applied all seven methods studied above to this data set and inspected the empirical distributions of the top ranked variables according to the obtained 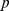-values and scores, respectively. File S1 contains the corresponding plots for the top 15 candidates for all methods (File S1, section 1.3). In summary, only FisherSum, Fisher10 and PADGE find a large number of variables with the desired pattern, where only a subgroup of observations in the disease group is up-regulated. FisherSum favors variables with wider distributions, as was expected because FS only centers but does not scale the observations. In contrast, among the top ranked variables of Fisher10 and PADGE, there are also candidates with small variation and only slight increases in the subgroup. Although even slight increases may have a biological relevance, they are often considered to be false positive results. Most candidates chosen by OS and ORT show small non-disease-specific subgroups. Kurtosis and Bartlett's test primarily detect variables with a single or very few outliers and thus are not suitable for our task either.

**Figure 3:**
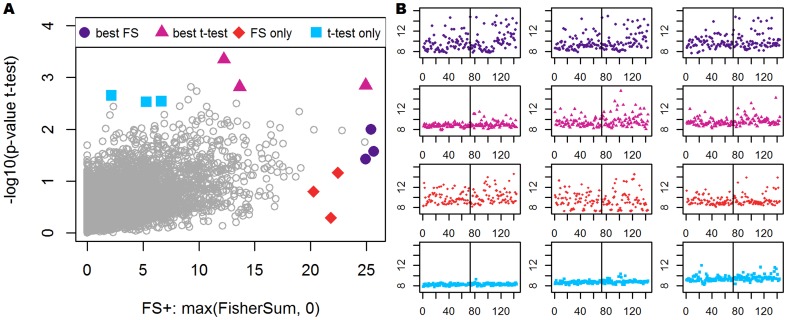
Comparison of FisherSum and 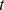-test on real data. Panel A: Score of 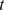-test (−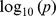) plotted against FisherSum, applied to ParkCHIP data [16] using weights 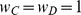. Each point represents a variable in the data set and the two corresponding measures. Highlighted are three variables for each of the following categories: highest score for FisherSum, smallest 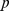-value for 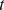-test, highly ranked in FisherSum but not in 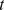-test, and vice versa. Panel B: 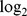-intensity plots corresponding to variables highlighted in panel A. Observations on the left-hand side of each plot represent the control group, and the right-hand side corresponds to patients with Parkinson's disease. To point out the differences in variation, we used the same scale for all variables.

Next, we focus on the results of FisherSum and compare it to the 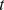-test. In Figure 3 (panel A) we plot the (−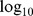) 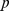-value of the 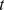-test against FS for each variable of the data set. The measures show moderate correlation (Pearson correlation 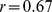), but especially the extremes are quite different and FS reveals some interesting additional variables. We picked three variables from each of the following categories: largest FS, smallest 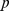-value in 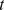-test, highly ranked in FS but poorly ranked in 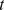-test, and vice versa. The corresponding (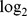)-intensity plots are shown in Figure 3 (panel B).

Finally, we searched the literature for the top candidates found by FisherSum, see Table 5. Our method finds candidates that have been associated to Parkinson's disease or neurodegenerative diseases in general as well as yet unknown candidates. We conclude that FisherSum is able to detect biologically relevant subgroups. Note that the two top-ranked variables are both assigned to the PALM2 gene with basically the same potential subgroup identified. The same holds true for the PDPK1 gene, with ranks 6 and 19 (not shown here). Hence presumably these candidates are true positives.

**Table 5:** FisherSum's top-ranked variables for ParkCHIP data [16].

FisherSum			*t*-test		
Rank	FS	Description	*p_t_*	Rank	Reference
1	25.7	PALM2	0.027	141	[18]
2	25.4	PALM2	0.010	42	[18]
3	24.9	MTHFR	0.037	217	[19]
4	24.9	GSK3A	0.001	2	—
5	24.9	PPP1R2P9	0.017	90	—
13	18.7	CALB2	0.083	636	[20]

For the above mentioned reasons, we chose 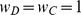. In the literature, some of them have already been associated with neurodegenerative diseases, in general or in particular with PD. According to 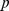-values of the 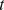-test (no adjustment for multiple testing), some of these candidates would have been missed.

## Discussion

We compared various tests and measures for subgroup detection on simulated and real data. We have shown that our FisherSum method outperforms existing methods over a large range of sample sizes if the subgroup size is small or moderate (up to about 25 percent). This limitation in subgroup size is in line with our initial aim to detect smaller subgroups. We use the 90 percent quantile in the disease group as cutpoint to construct a contingency table (Table 1) as it is used for Fisher's exact test. This corresponds to a minimal subgroup size of interest equal to 10 percent and also yields good results for moderately larger subgroup sizes.

It has been shown previously, that the best strategy for subgroup detection may depend on the combination of sample and subgroup size as well as on the true degree of deviation from the null situation with no differential expression. To date, the influence of the underlying distribution of the subgroup has been ignored for the most part. By comparing the commonly assumed distribution with two others scenarios, we point out that the true underlying distribution does have an effect on the performance of the methods that were included in our simulation study. However, having a closer look at the results of scenarios I and II, we do not notice a fundamental difference between the test performances. In our opinion, the widely assumed subgroup distribution 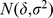 is too simple, because of the fairly good separation of the subgroup from the remainder of the disease group for larger values of 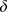. On the other hand, the distribution for scenario II makes it quite hard to detect the true subgroup, as it always overlaps with the other observations. Due to the similar results for these two extreme situations, we expect comparable conclusions for mixtures of these scenarios. We included scenario III to demonstrate that the class of subgroup problems is not limited to location shift problems. To our knowledge, an increased variance in a patient subgroup has not been discussed before. If non-specific subgroups are included in the null situation, none of the compared methods shows good performance for small subgroups when compared to LR. This underlines that novel methods are required if new classes of subgroup distributions are considered, because the commonly used subgroup detection methods might fail.

The aim of our simulation study was to provide an overall comparison of several methods for the detection of patient subgroups. Therefore, we chose three different distributions for the subgroup and within these scenarios we gradually increased the degree of deviation from the null situation. Thus, we are able to check for a uniformly best method or assess the performance of competing methods across a certain range of alternatives rather than focussing on a single parameter value. For example, in our simulation, comparing the 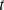-test and Bartlett's test for homoscedasticity, the 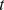-test performs better for small deviations from the null situation, whereas Bartlett's test is better for larger deviations for certain combinations of sample and subgroup size. In short, the two major advantages of our study design arise from the consideration of a wide range of alternatives for several distributions as well as from the incorporation of a new type of null situation, where subgroups are present in both groups. Results of simulation and real data analysis show that our new method is less likely to yield false positive results in terms of these non-disease-specific variables.

Since our data is drawn from normal distributions with variance equal to one, the relevant values of the deviation (e.g. the required 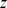 value to attain a certain AUC value, or the value 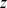 where one test outperforms another one) might not be appropriate for real data. In cases of doubt, we recommend to adapt this study with variances that fit a specific application. Differences in the amount of variance in the data may be due to different biological variances or different technical variances that correspond to different omics techniques. To generate data sets that are as close to real data as possible, variances might be drawn from empirical distributions of variances from real data sets measured with the technique of interest.

At this point, we did not include multivariate methods that are commonly applied in the field of statistics. A univariate approach is appropriate, if a study either aims explicitly at the detection of univariate biomarker candidates or if dimensionality reduction is required. Candidates of univariate analyses may be combined by multivariate procedures like hierarchical clustering afterwards. Note that due to the different selection criteria the results will differ from clustering results using the common approach. For the detection of small subgroups that are present in a small proportion of the observed variables, specific approaches are required. An important next step is to derive an appropriate method for the combination of subgroup indicating variables.

As already stated in the beginning, the assumption of at most one single subgroup per variable does not exclude the existence of several subgroups in the disease group regarding the multivariate dataset. The distributional pattern that we use in the simulation study only requires that if the disease group is composed of more than one subgroup, then the sets of effected variables are disjoint. For methods presented previously in the literature as well as for FisherSum, the subgroup structure may be assessed in more detail by an additional subsequent analysis. Just a short remark on the effect of multiple subgroups that are present in the same variable. In this case one has to differentiate further according to the direction of the regulation in the subgroups. If the subgroup regulations show the same direction, e.g. up-regulation, the variable is even easier to detect for the majority of the presented methods. On the other hand, if a variable shows at least one up-regulated and at the same time at least one down-regulated subgroup, the performance of the different methods varies. FisherSum for example is not affected in this case, while outlier sum could perform worse due to increased variation in the variable. In this context, an alternative interpretation of the third scenario with increased variance in the subgroup is possible. Consider the subgroup observations above and below the mean as two different subgroups. One of them is up-regulated, the other one down-regulated with the simplification that the degree of deviation is equal for both subgroups. The actual size of each subgroup would equal 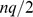.

The presented methods are appropriate for explorative analyses and for the generation of new hypotheses on potential patient subgroups. Thus, the resulting candidate variables and the respective marked patient subgroups require further biological validation. Apart from correlations within the data sets one can try to correlate the potentially subgroup indicating variables with clinical variables such as survival times to check for their relevance. The explicit determination of the potential patient subgroup is of particular interest, but it is provided directly only for OS and ORT. Obviously, for FisherSum, it is a natural choice to assign the samples above the chosen cutoff to the potential subgroup. This will work well, if the true subgroup size is about 10 percent. Another way to identify subgroups based on the set of potentially interesting variables is to conduct hierarchical clustering with the top (e.g. 50) candidates of FisherSum. Assuming that a small number of variables indicate the same subgroup, an existing subgroup structure is expected to become apparent.

The presented results for our new method are promising as we are able to detect disease-related variables in real data. One option for future research is the introduction of scoring methods that allow for the separate optimization of sensitivity and specificity, respectively. There are several ways to adjust our method to the specific characteristics of a data set, for example by choosing a different quantile, by varying the weights 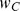 and 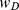, by defining thresholds for 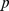-values of Fisher's exact test, or by incorporating the distance between the potential subgroup and the remainder of the group. An important goal for the future is to evaluate different approaches for the combination of subgroup indicating variables in order to gain insight into the overall patient subgroup structure.

## Supporting Information

File S1
**Figure S1, In-depth comparison of different methods' AUC values for the composite null situation.** The study includes all combinations of sample sizes 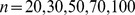, subgroup proportions 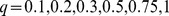, and scenarios 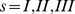 with 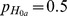. AUC values are plotted against the deviation 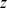. **Figure S2, In-depth comparison of different methods' AUC values for the simple null situation.** The study includes all combinations of sample sizes 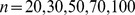, subgroup proportions 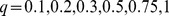, and scenarios 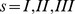 with 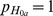. AUC values are plotted against the deviation 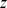. **Figure S3, (log2-)intensity plots of the top candidates for ParkCHIP data.** To compare the considered methods, the respective top 15 candidates of each method are shown in (log2-)intensity plots. We used controls and Parkinson patients from the ParkCHIP project. **Table S1, Overview scenario I, composite null situation.** Best tests for all combinations of sample size 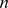 and subgroup proportions 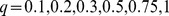 in scenario I, 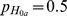, neatly arranged in a color-coded table. **Table S2, Overview scenario II, composite null situation.** Best tests for all combinations of sample size 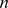 and subgroup proportions 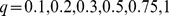 in scenario II, 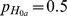, neatly arranged in a color-coded table. **Table S3, Overview scenario III, composite null situation.** Best tests for all combinations of sample size 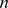 and subgroup proportions 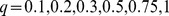 in scenario III, 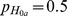, neatly arranged in a color-coded table. **Table S4, Overview scenario I, simple null situation.** Best tests for all combinations of sample size 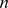 and subgroup proportions 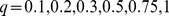 in scenario I, 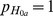, neatly arranged in a single table. **Table S5, Overview scenario II, simple null situation.** Best tests for all combinations of sample size 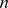 and subgroup proportions 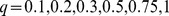 in scenario II, 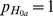, neatly arranged in a single table. **Table S6,Overview scenario III, simple null situation.** Best tests for all combinations of sample size 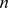 and subgroup proportions 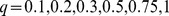 in scenario III, 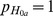, neatly arranged in a single table.(PDF)
