# Supplementary material for: Detection of Patient Subgroups with Differential Expression in Omics Data: A Comprehensive Comparison of Univariate Measures
Source: PLoS One. 2013 Nov 22;8(11):e79380. doi: 10.1371/journal.pone.0079380 (PMC3838370; doi:10.1371/journal.pone.0079380)
Supplement: File S1 — Figure S1, In-depth comparison of different methods' AUC values for the composite null situation. The study includes all combinations of sample sizes , subgroup proportions , and scenarios with . AUC values are plotted against the deviation . Figure S2, In-depth comparison of different methods' AUC values for the simple null situation. The study includes all combinations of sample sizes , subgroup proportions , and scenarios with . AUC values are plotted against the deviation . Figure S3, (log2-)intensity plots of the top candidates for ParkCHIP data. To compare the considered methods, the respective top 15 candidates of each method are shown in (log2-)intensity plots. We used controls and Parkinson patients from the ParkCHIP project. Table S1, Overview scenario I, composite null situation. Best tests for all combinations of sample size and subgroup proportions in scenario I, , neatly arranged in a color-coded table. Table S2, Overview scenario II, composite null situation. Best tests for all combinations of sample size and subgroup proportions in scenario II, , neatly arranged in a color-coded table. Table S3, Overview scenario III, composite null situation. Best tests for all combinations of sample size and subgroup proportions in scenario III, , neatly arranged in a color-coded table. Table S4, Overview scenario I, simple null situation. Best tests for all combinations of sample size and subgroup proportions in scenario I, , neatly arranged in a single table. Table S5, Overview scenario II, simple null situation. Best tests for all combinations of sample size and subgroup proportions in scenario II, , neatly arranged in a single table. Table S6,Overview scenario III, simple null situation. Best tests for all combinations of sample size and subgroup proportions in scenario III, , neatly arranged in a single table. (PDF) [file pone.0079380.s001.pdf]

Supplement to:

Detection of patient subgroups with differential expression in omics data: a comprehensive comparison of univariate measures

Ahrens et al.

Contents

1 Figures **2**
1.1 Simulation results (composite null situation) 2
1.2 Simulation results (simple null situation) 21
1.3 Top candidates of different measures for ParkCHIP data . . . 40

2 Pseudocode **49**
2.1 FisherSum and Fisher10 49
2.2 PADGE 50
2.3 ORT 51
2.4 OS 52

1 Figures

1.1 Simulation results (composite null situation)

In the following we provide the simulation results that complement the results in Figure 1 in the main paper. The study includes all combinations of sample sizes $n = 20, 30, 50, 70, 100$ and subgroup proportions $q = 0.1, 0.2, 0.3, 0.5, 0.75, 1$, and the null situation is comprised of H_{0a} and H_{0b} in equal shares (i.e. $p_{H_{0a}} = 0.5$).
Table S1- S3 give summary of the best tests for each combination of sample size n and subgroup proportion q for the different scenarios for the composite null situation with $p_{H_{0a}} = 0.5$. In general, t-test performs best, if the majority of the disease is upregulated, i.e. for subgroups with $q \geq 0.5$, in scenario I and II. For smaller subgroup sizes, the best test depends on the degree of deviation. Whereas the t-test is able to detect very small deviations, our FisherSum is best for rather moderate deviations, and for larger deviations Bartlett's test yields best results. Please note that for large deviations several tests gain AUC values virtually equal to 1, see following pages. Even in scenario III (bidirectional deregulation), FisherSum yields slightly better results than Bartlett's test for small deviations and subgroup proportions.

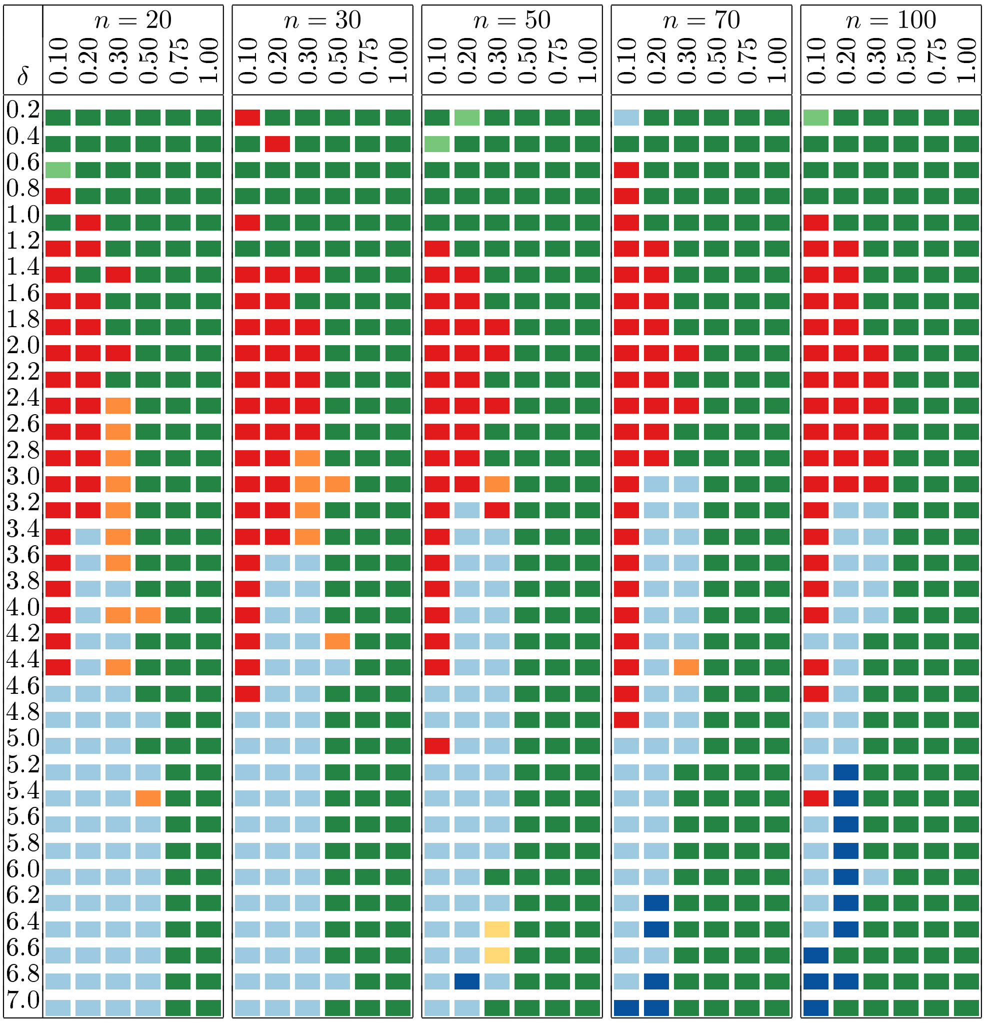

Table S1: Best tests for all combinations of sample size n and subgroup proportions $q = 0.1, 0.2, 0.3, 0.5, 0.75, 1$ in scenario I, $p_{H_{0a}} = 0.5$.
Color code: ■ FisherSum, ■ Bartlett's test, ■ t-test, ■ ORT, ■ Kurtosis, ■ OS, ■ PADGE.

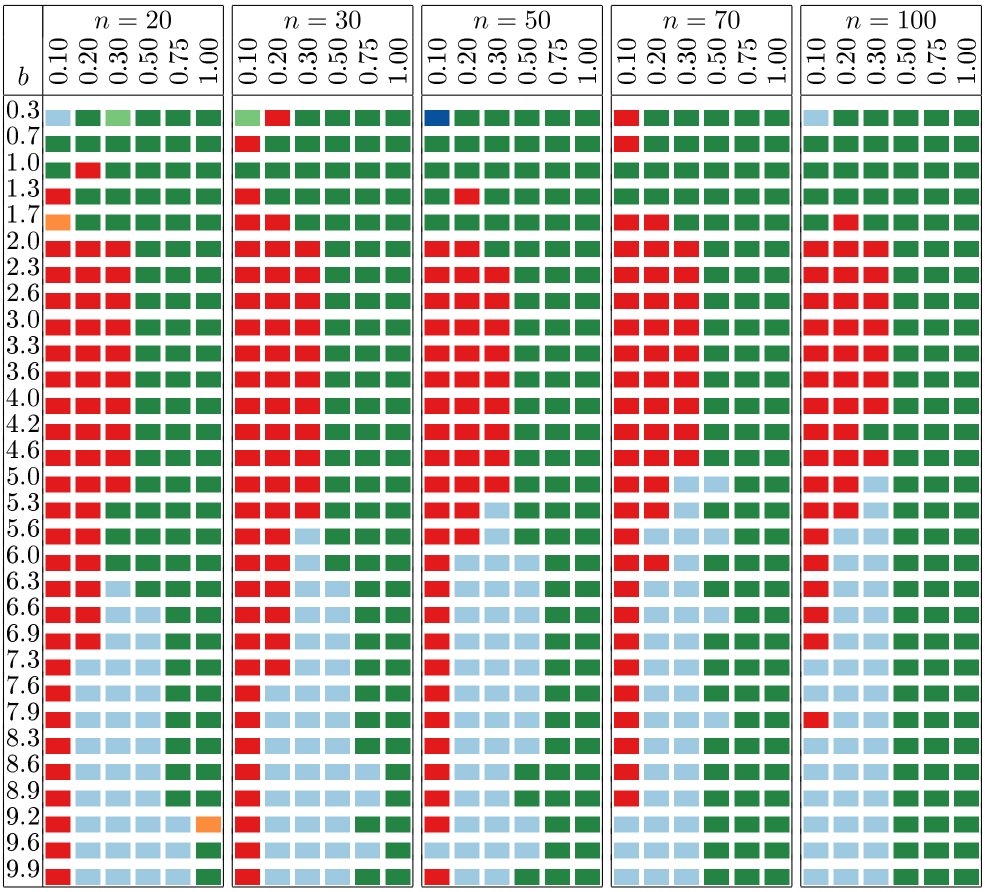

Table S2: Best tests for all combinations of sample size n and subgroup proportions $q = 0.1, 0.2, 0.3, 0.5, 0.75, 1$ in scenario II, $p_{H_{0a}} = 0.5$.
Color code: ■ FisherSum, ■ Bartlett's test, ■ t-test, ■ ORT, ■ Kurtosis, ■ OS, ■ PADGE.

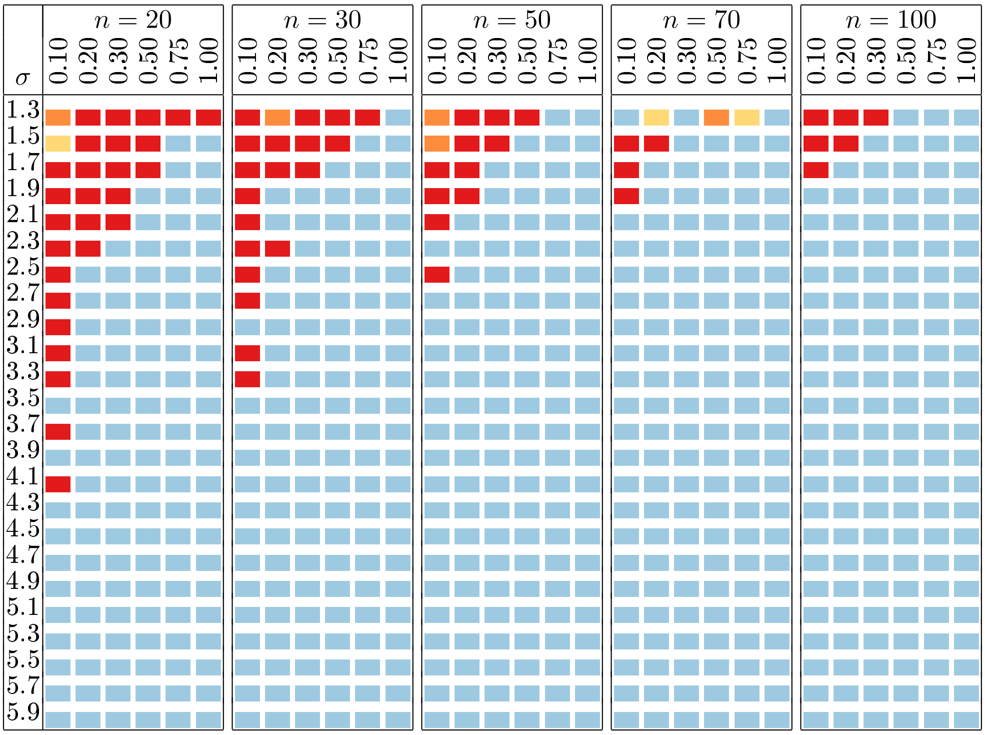

Table S3: Best tests for all combinations of sample size n and subgroup proportions $q = 0.1, 0.2, 0.3, 0.5, 0.75, 1$ in scenario III, $p_{H_{0a}} = 0.5$.
Color code: FisherSum, Bartlett's test, t-test, ORT, Kurtosis, OS, PADGE.

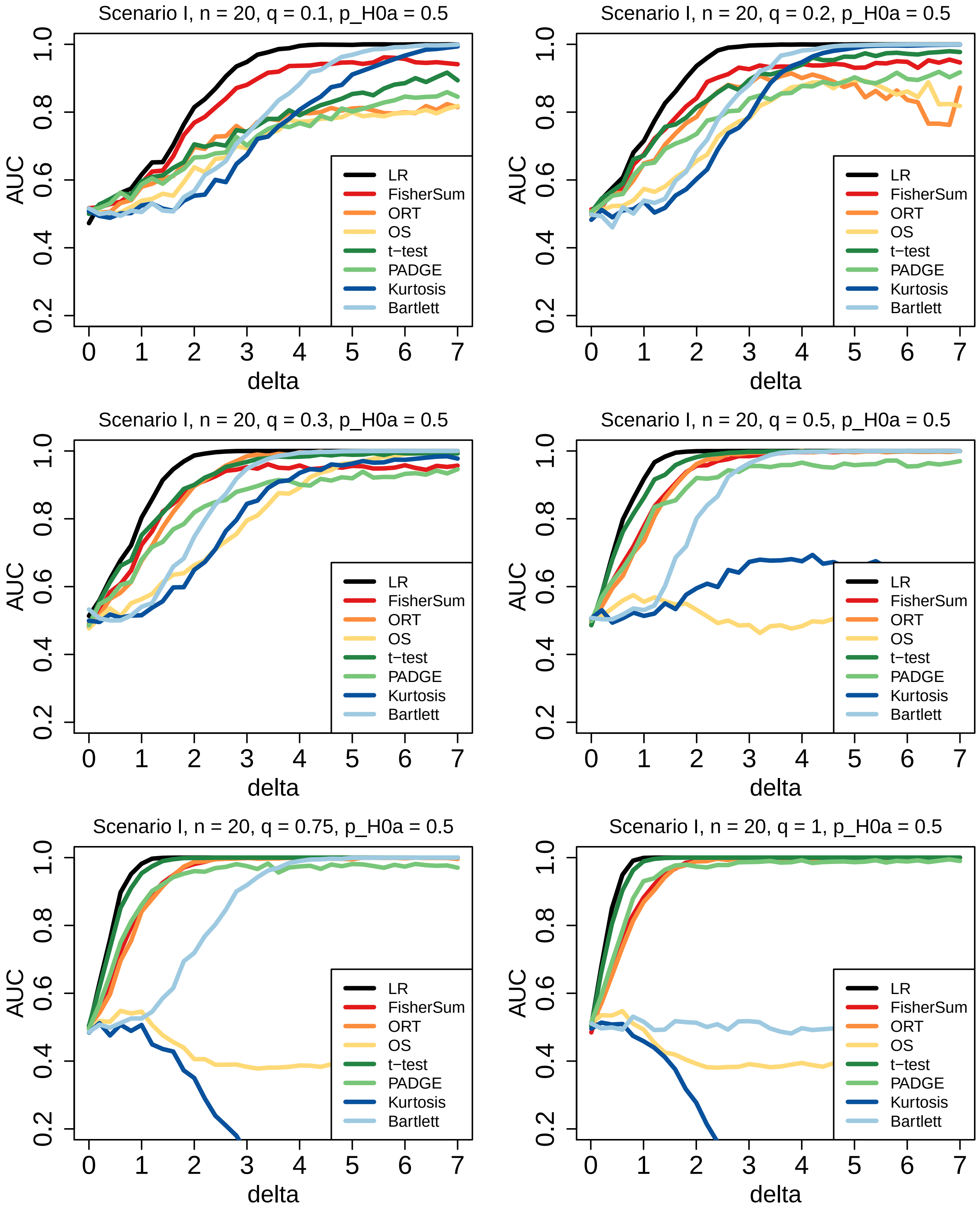

Scenario I, n = 20, q = 0.1, p_H0a = 0.5
Scenario I, n = 20, q = 0.2, p_H0a = 0.5
Scenario I, n = 20, q = 0.3, p_H0a = 0.5
Scenario I, n = 20, q = 0.5, p_H0a = 0.5
Scenario I, n = 20, q = 0.75, p_H0a = 0.5
Scenario I, n = 20, q = 1, p_H0a = 0.5
AUC
0.2
0.4
0.6
0.8
1.0
delta
0
1
2
3
4
5
6
7
LR
FisherSum
ORT
OS
t−test
PADGE
Kurtosis
Bartlett

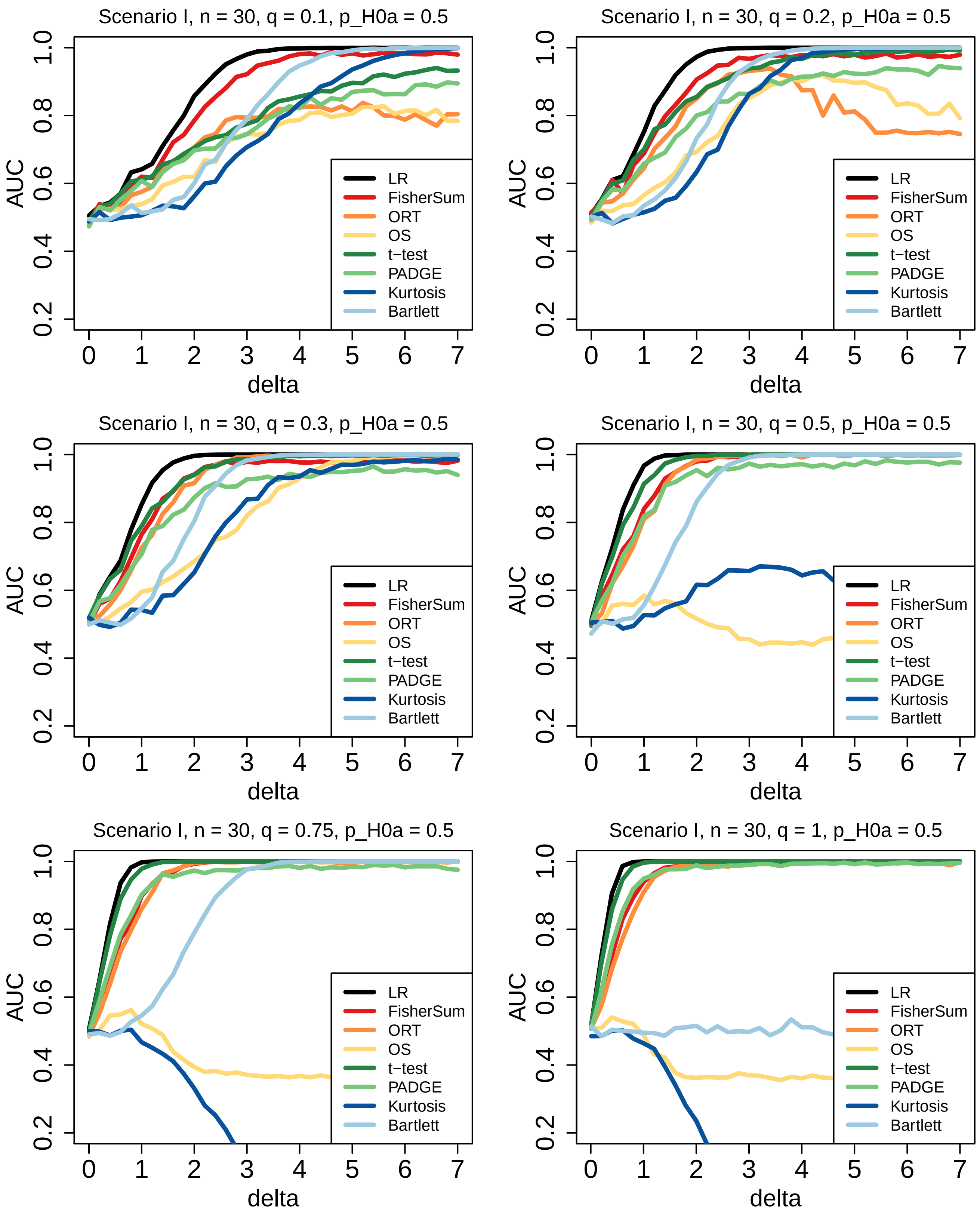
Scenario I, n = 30, q = 0.1, p_H0a = 0.5
Scenario I, n = 30, q = 0.2, p_H0a = 0.5
Scenario I, n = 30, q = 0.3, p_H0a = 0.5
Scenario I, n = 30, q = 0.5, p_H0a = 0.5
Scenario I, n = 30, q = 0.75, p_H0a = 0.5
Scenario I, n = 30, q = 1, p_H0a = 0.5
AUC
0.2
0.4
0.6
0.8
1.0
delta
0
1
2
3
4
5
6
7
LR
FisherSum
ORT
OS
t−test
PADGE
Kurtosis
Bartlett

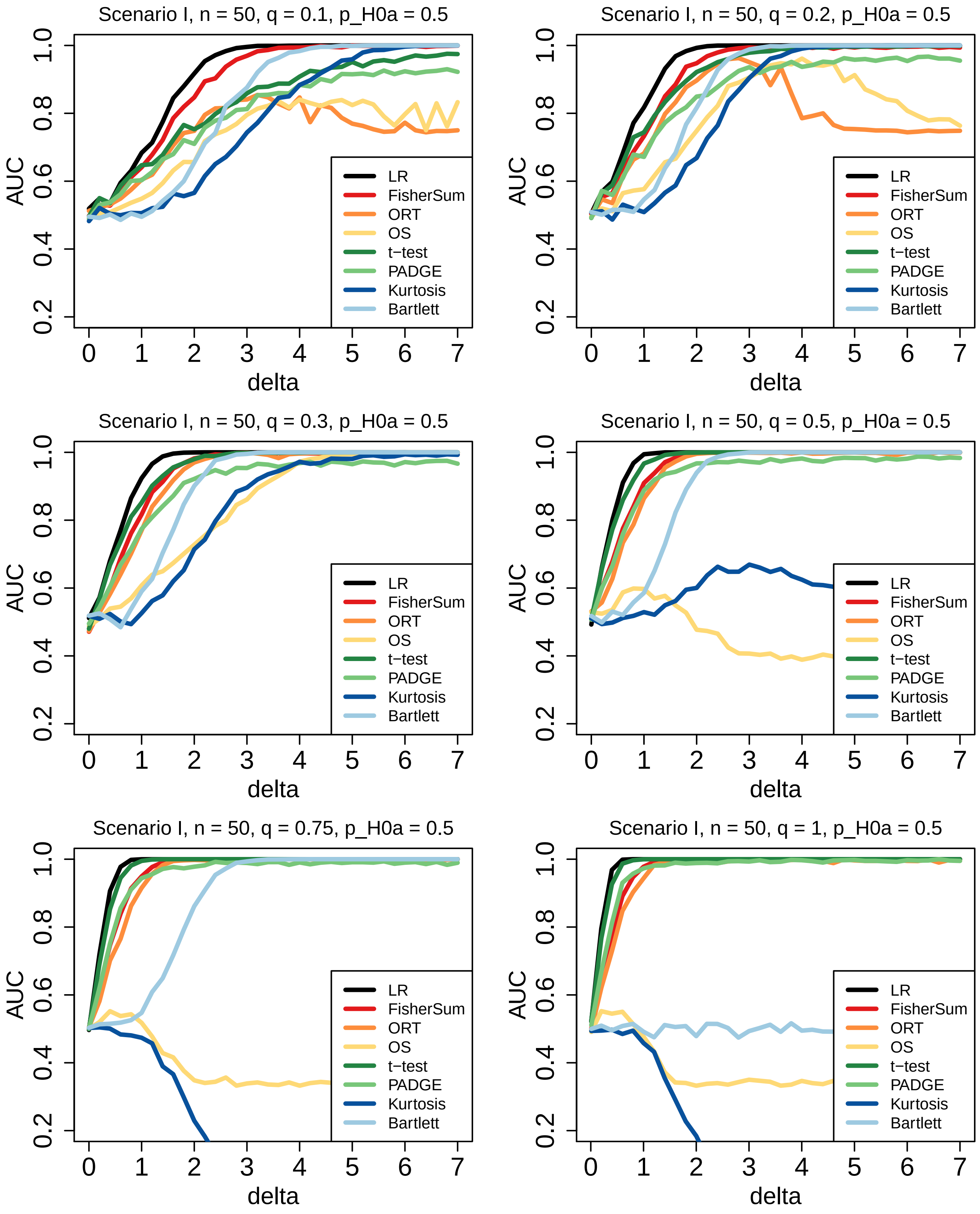
Scenario I, n = 50, q = 0.1, p_H0a = 0.5
Scenario I, n = 50, q = 0.2, p_H0a = 0.5
Scenario I, n = 50, q = 0.3, p_H0a = 0.5
Scenario I, n = 50, q = 0.5, p_H0a = 0.5
Scenario I, n = 50, q = 0.75, p_H0a = 0.5
Scenario I, n = 50, q = 1, p_H0a = 0.5
AUC
0.2
0.4
0.6
0.8
1.0
delta
0
1
2
3
4
5
6
7
LR
FisherSum
ORT
OS
t−test
PADGE
Kurtosis
Bartlett

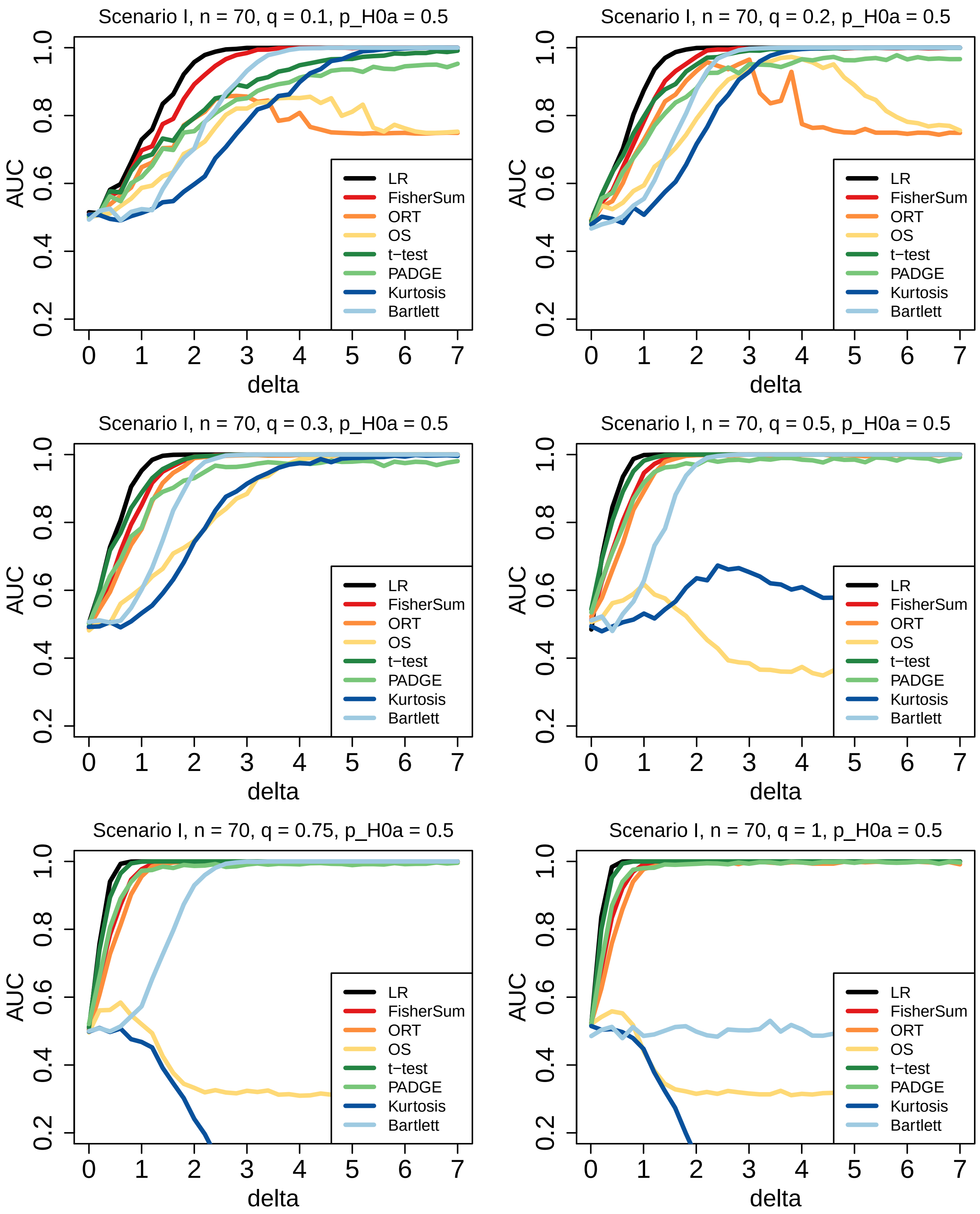

Scenario I, n = 70, q = 0.1, p_H0a = 0.5
Scenario I, n = 70, q = 0.2, p_H0a = 0.5
Scenario I, n = 70, q = 0.3, p_H0a = 0.5
Scenario I, n = 70, q = 0.5, p_H0a = 0.5
Scenario I, n = 70, q = 0.75, p_H0a = 0.5
Scenario I, n = 70, q = 1, p_H0a = 0.5
AUC
delta
LR
FisherSum
ORT
OS
t−test
PADGE
Kurtosis
Bartlett

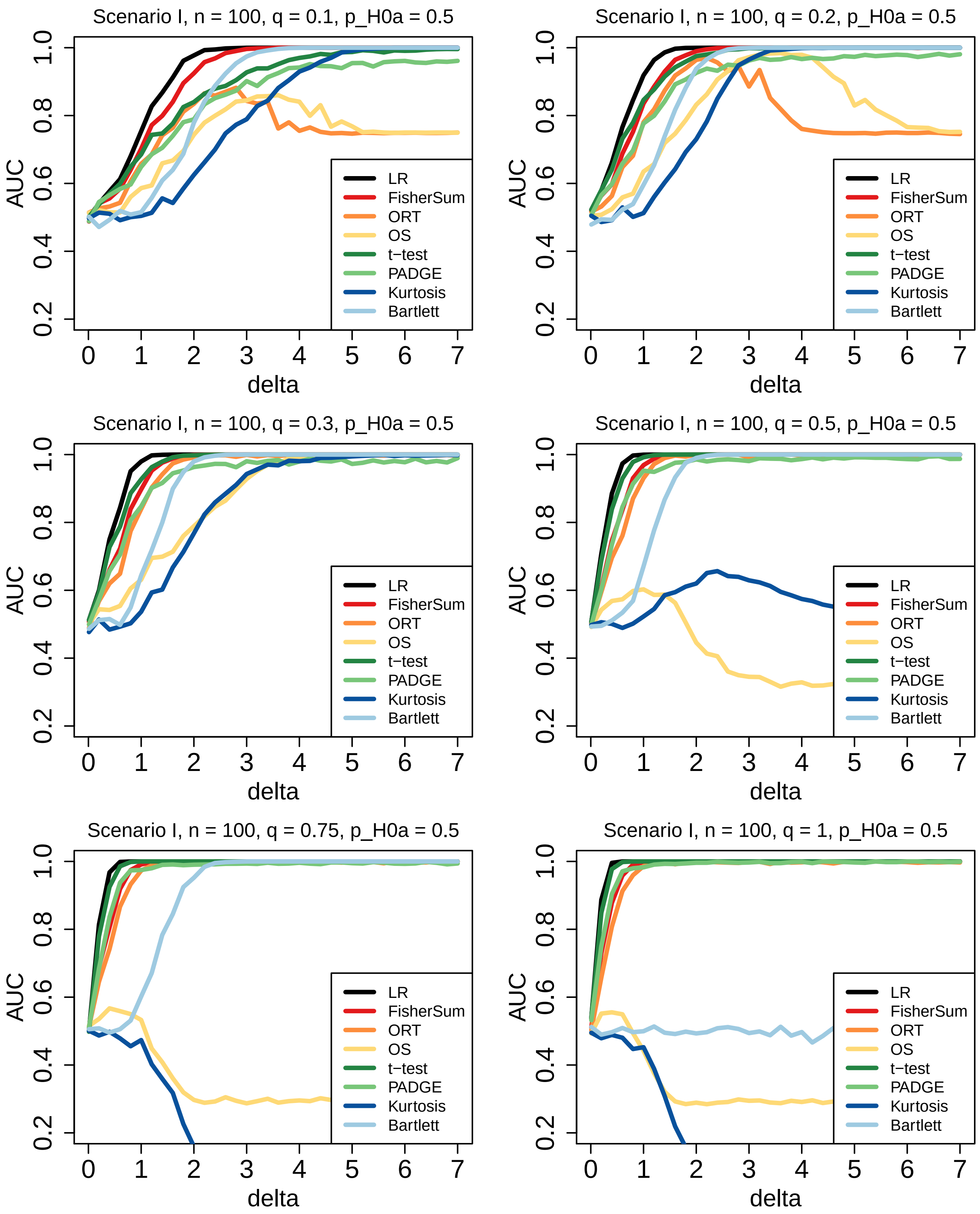
Scenario I, n = 100, q = 0.1, p_H0a = 0.5
Scenario I, n = 100, q = 0.2, p_H0a = 0.5
Scenario I, n = 100, q = 0.3, p_H0a = 0.5
Scenario I, n = 100, q = 0.5, p_H0a = 0.5
Scenario I, n = 100, q = 0.75, p_H0a = 0.5
Scenario I, n = 100, q = 1, p_H0a = 0.5
AUC
delta
0.2
0.4
0.6
0.8
1.0
0
1
2
3
4
5
6
7
LR
FisherSum
ORT
OS
t−test
PADGE
Kurtosis
Bartlett

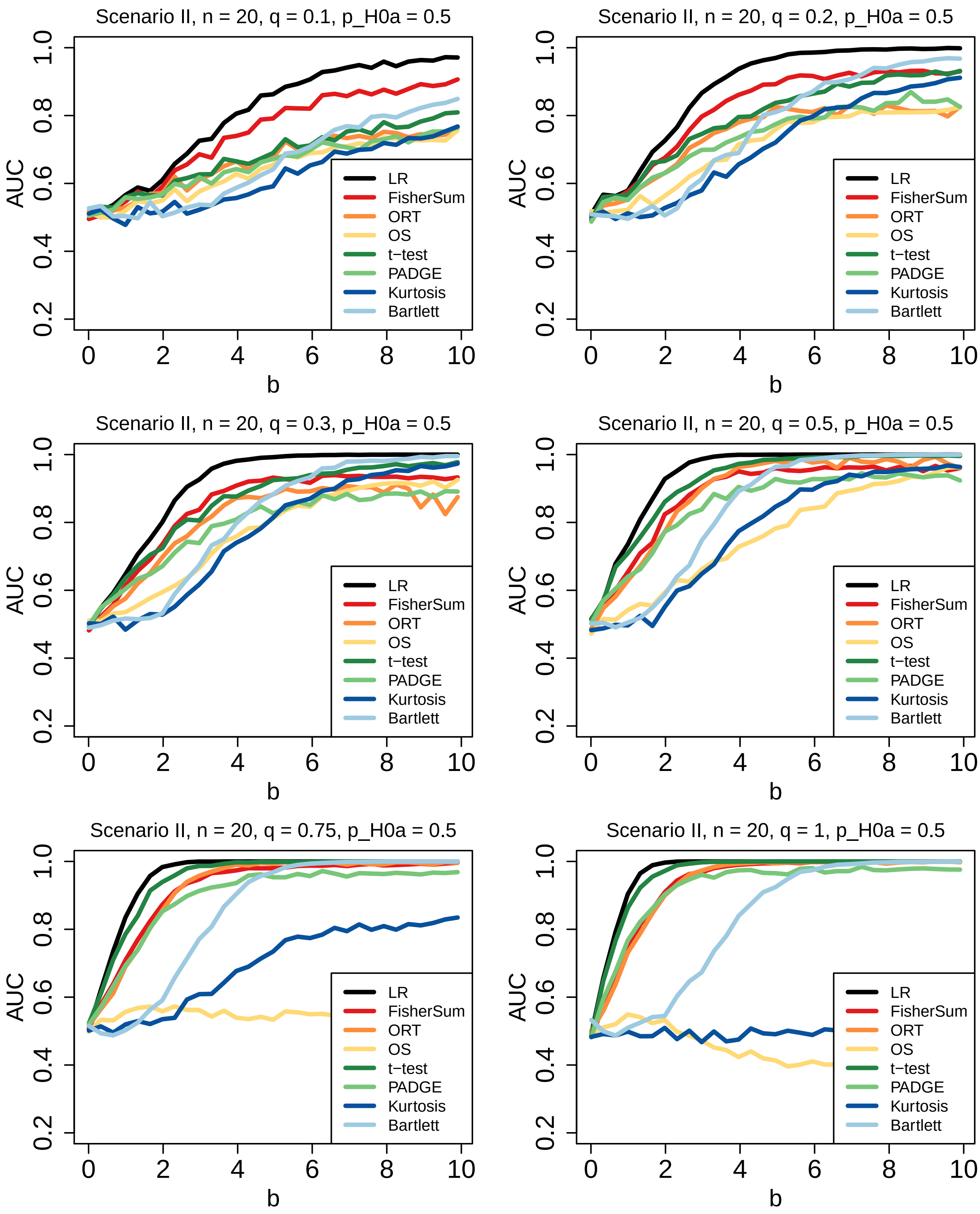
Scenario II, n = 20, q = 0.1, p_H0a = 0.5
Scenario II, n = 20, q = 0.2, p_H0a = 0.5
Scenario II, n = 20, q = 0.3, p_H0a = 0.5
Scenario II, n = 20, q = 0.5, p_H0a = 0.5
Scenario II, n = 20, q = 0.75, p_H0a = 0.5
Scenario II, n = 20, q = 1, p_H0a = 0.5
AUC
b
LR
FisherSum
ORT
OS
t−test
PADGE
Kurtosis
Bartlett

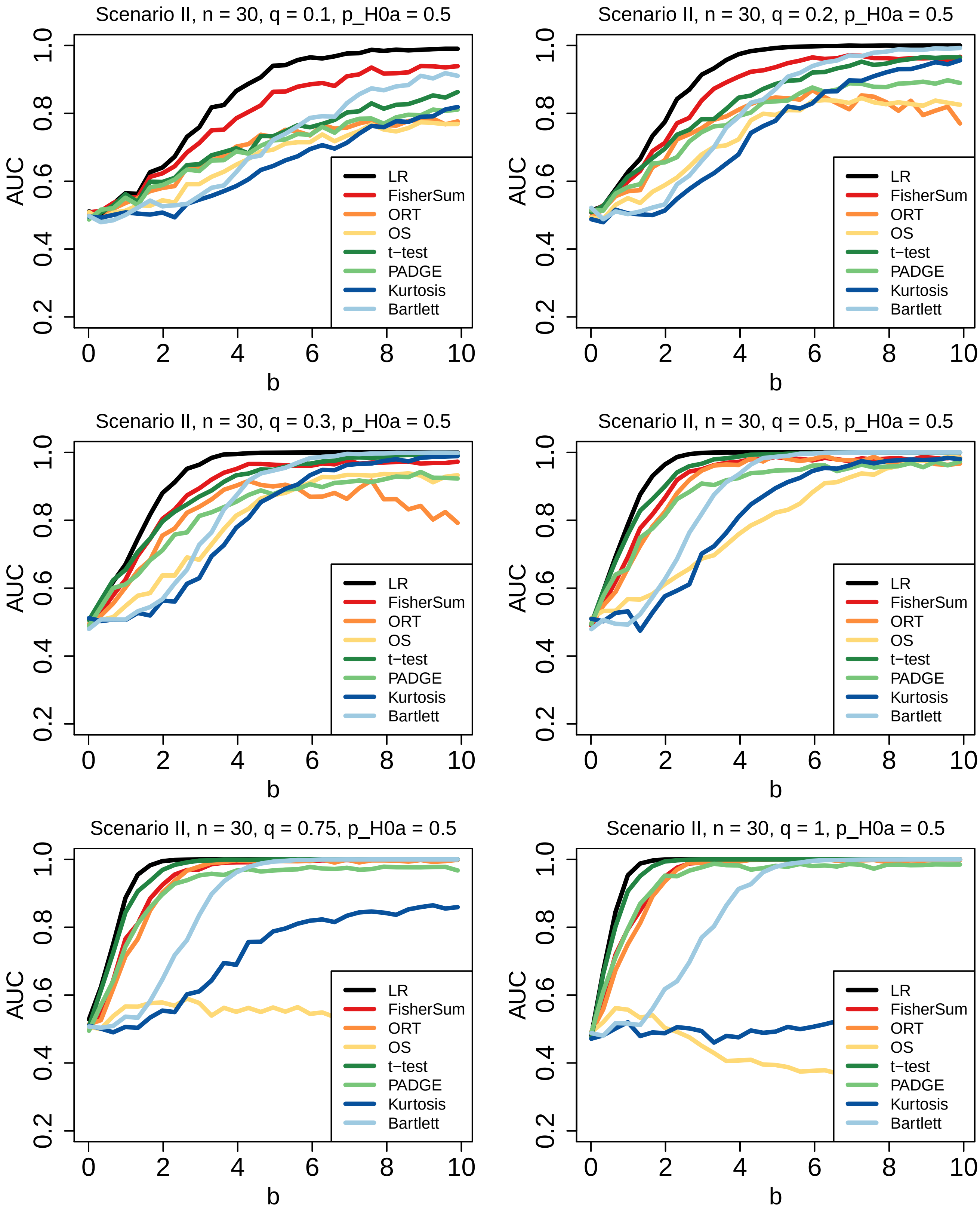
Scenario II, n = 30, q = 0.1, p_H0a = 0.5
Scenario II, n = 30, q = 0.2, p_H0a = 0.5
Scenario II, n = 30, q = 0.3, p_H0a = 0.5
Scenario II, n = 30, q = 0.5, p_H0a = 0.5
Scenario II, n = 30, q = 0.75, p_H0a = 0.5
Scenario II, n = 30, q = 1, p_H0a = 0.5
AUC
b
0.2
0.4
0.6
0.8
1.0
0
2
4
6
8
10
LR
FisherSum
ORT
OS
t−test
PADGE
Kurtosis
Bartlett

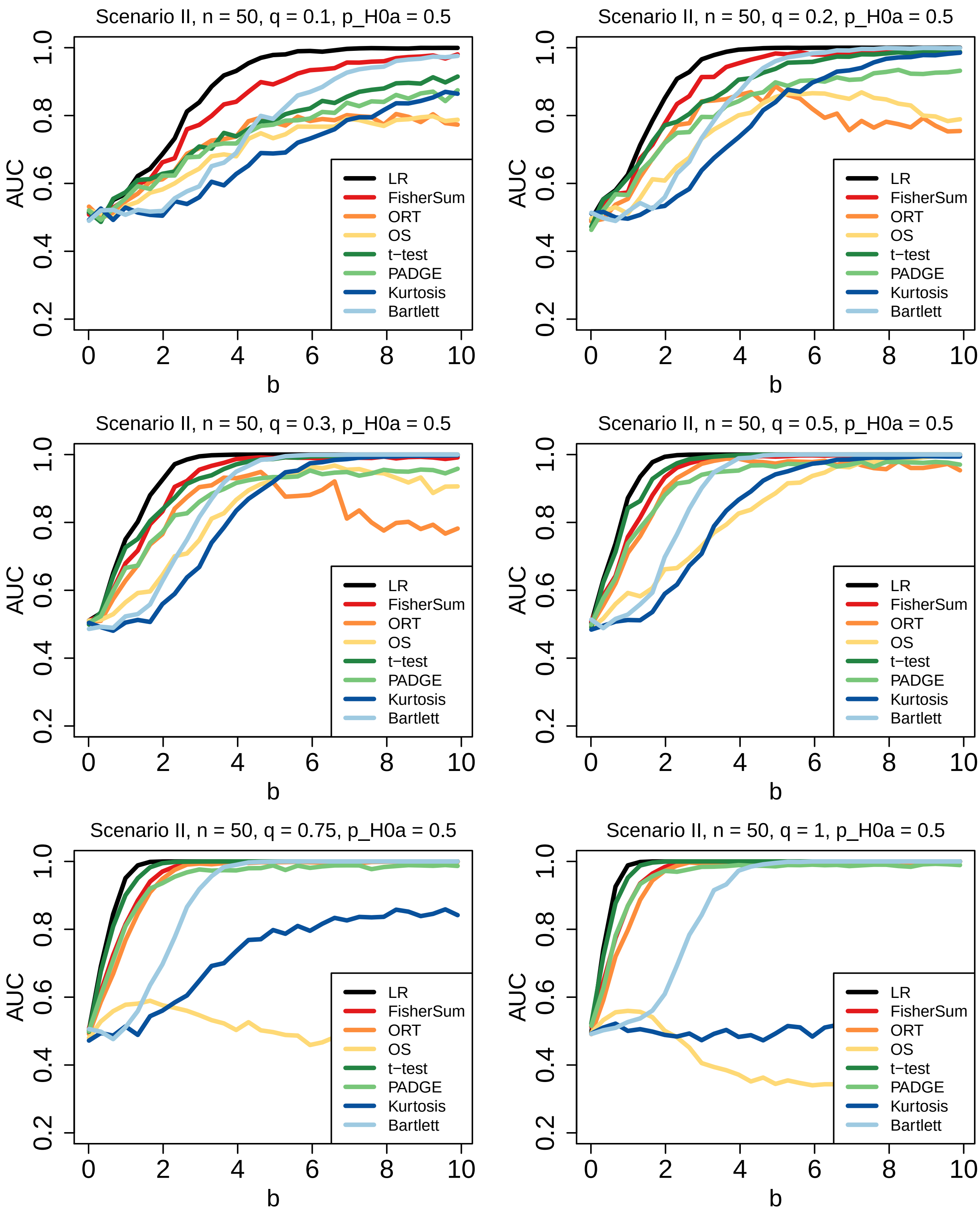
Scenario II, n = 50, q = 0.1, p_H0a = 0.5
Scenario II, n = 50, q = 0.2, p_H0a = 0.5
Scenario II, n = 50, q = 0.3, p_H0a = 0.5
Scenario II, n = 50, q = 0.5, p_H0a = 0.5
Scenario II, n = 50, q = 0.75, p_H0a = 0.5
Scenario II, n = 50, q = 1, p_H0a = 0.5
AUC
b
0.2
0.4
0.6
0.8
1.0
0
2
4
6
8
10
LR
FisherSum
ORT
OS
t−test
PADGE
Kurtosis
Bartlett

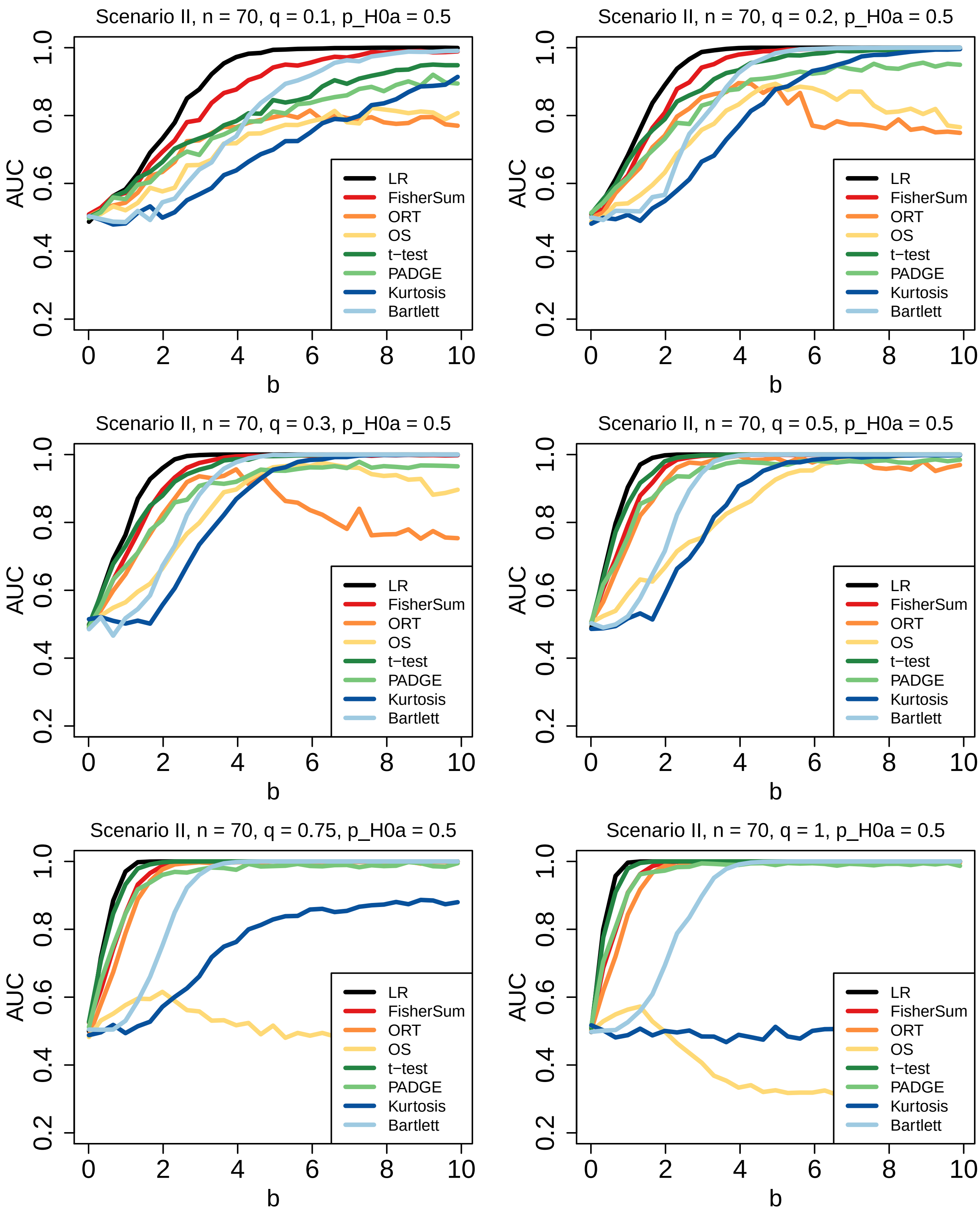
Scenario II, n = 70, q = 0.1, p_H0a = 0.5
Scenario II, n = 70, q = 0.2, p_H0a = 0.5
Scenario II, n = 70, q = 0.3, p_H0a = 0.5
Scenario II, n = 70, q = 0.5, p_H0a = 0.5
Scenario II, n = 70, q = 0.75, p_H0a = 0.5
Scenario II, n = 70, q = 1, p_H0a = 0.5
AUC
b
LR
FisherSum
ORT
OS
t−test
PADGE
Kurtosis
Bartlett

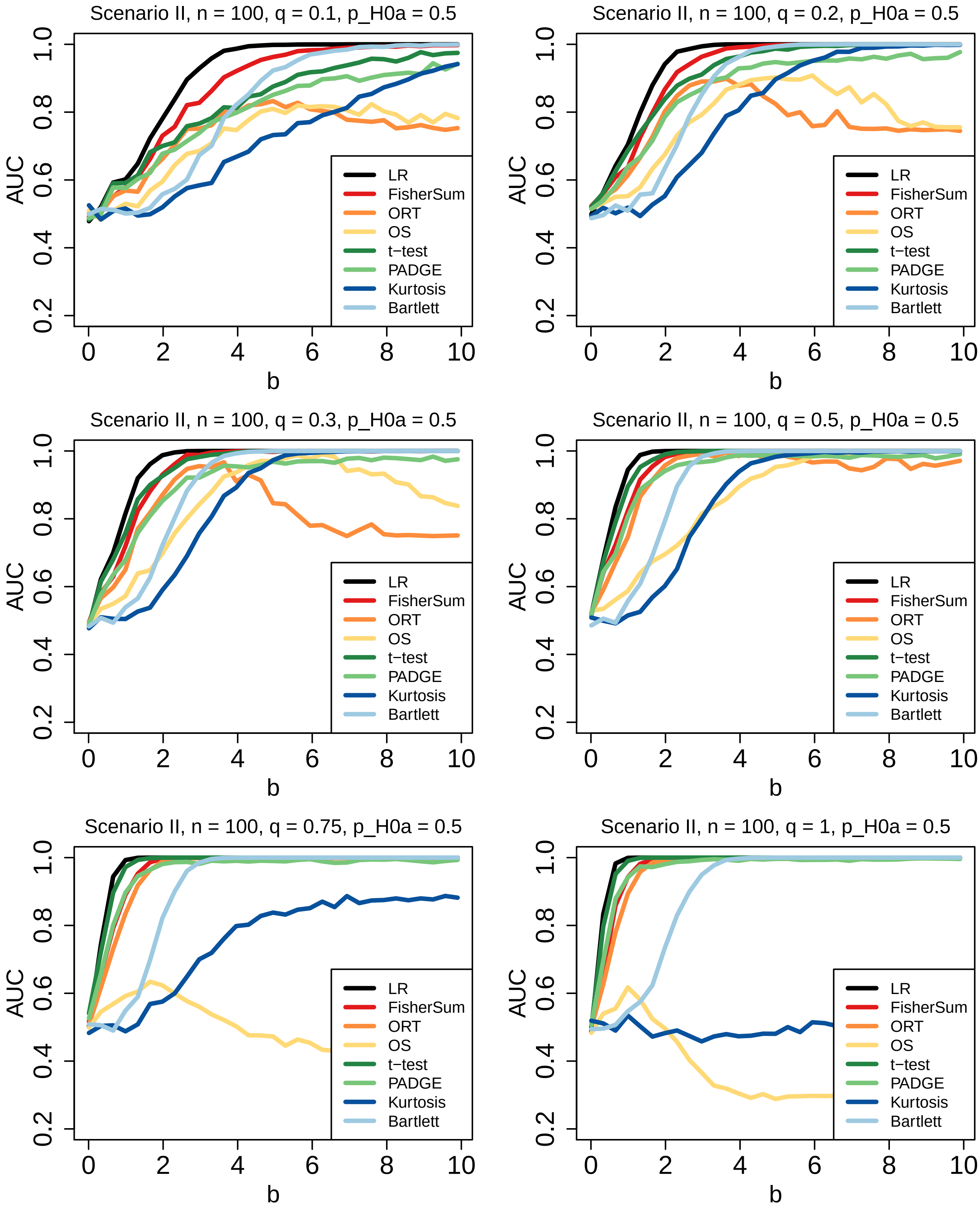

Scenario II, n = 100, q = 0.1, p_H0a = 0.5
Scenario II, n = 100, q = 0.2, p_H0a = 0.5
Scenario II, n = 100, q = 0.3, p_H0a = 0.5
Scenario II, n = 100, q = 0.5, p_H0a = 0.5
Scenario II, n = 100, q = 0.75, p_H0a = 0.5
Scenario II, n = 100, q = 1, p_H0a = 0.5
AUC
b
LR
FisherSum
ORT
OS
t−test
PADGE
Kurtosis
Bartlett

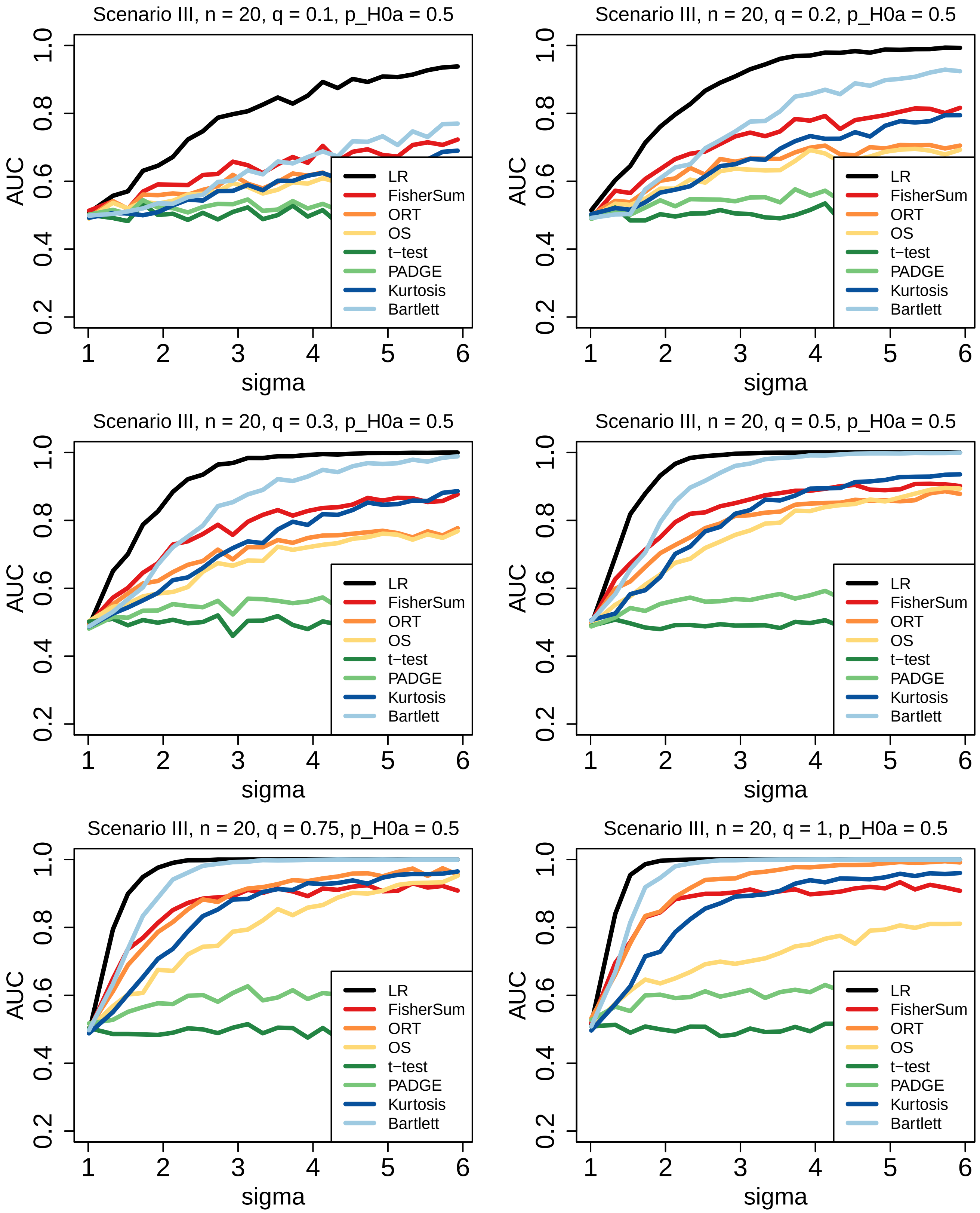
Scenario III, n = 20, q = 0.1, p_H0a = 0.5
Scenario III, n = 20, q = 0.2, p_H0a = 0.5
Scenario III, n = 20, q = 0.3, p_H0a = 0.5
Scenario III, n = 20, q = 0.5, p_H0a = 0.5
Scenario III, n = 20, q = 0.75, p_H0a = 0.5
Scenario III, n = 20, q = 1, p_H0a = 0.5
AUC
sigma
LR
FisherSum
ORT
OS
t−test
PADGE
Kurtosis
Bartlett

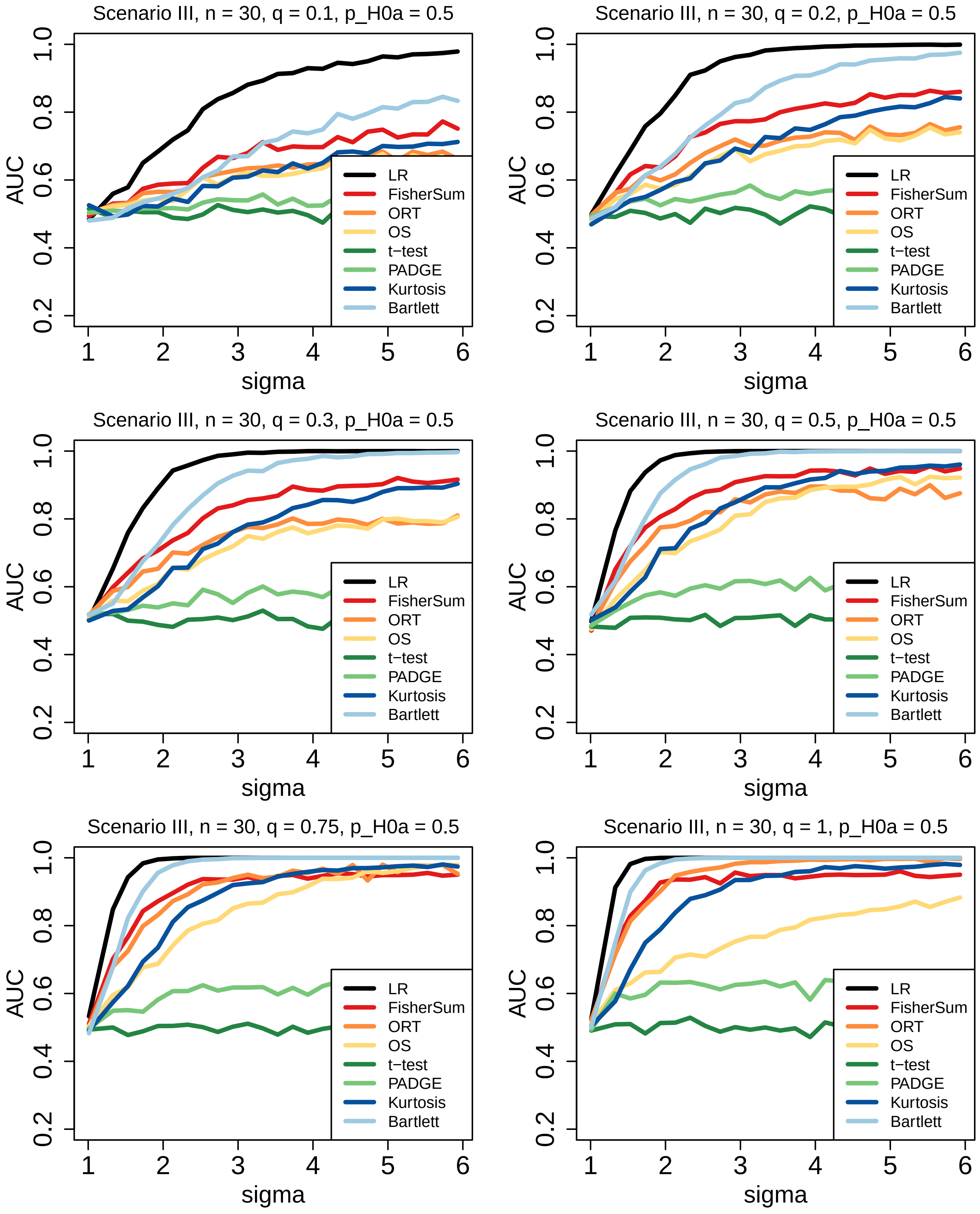
Scenario III, n = 30, q = 0.1, p_H0a = 0.5
Scenario III, n = 30, q = 0.2, p_H0a = 0.5
Scenario III, n = 30, q = 0.3, p_H0a = 0.5
Scenario III, n = 30, q = 0.5, p_H0a = 0.5
Scenario III, n = 30, q = 0.75, p_H0a = 0.5
Scenario III, n = 30, q = 1, p_H0a = 0.5
AUC
0.2 0.4 0.6 0.8 1.0
sigma
1 2 3 4 5 6
LR
FisherSum
ORT
OS
t−test
PADGE
Kurtosis
Bartlett

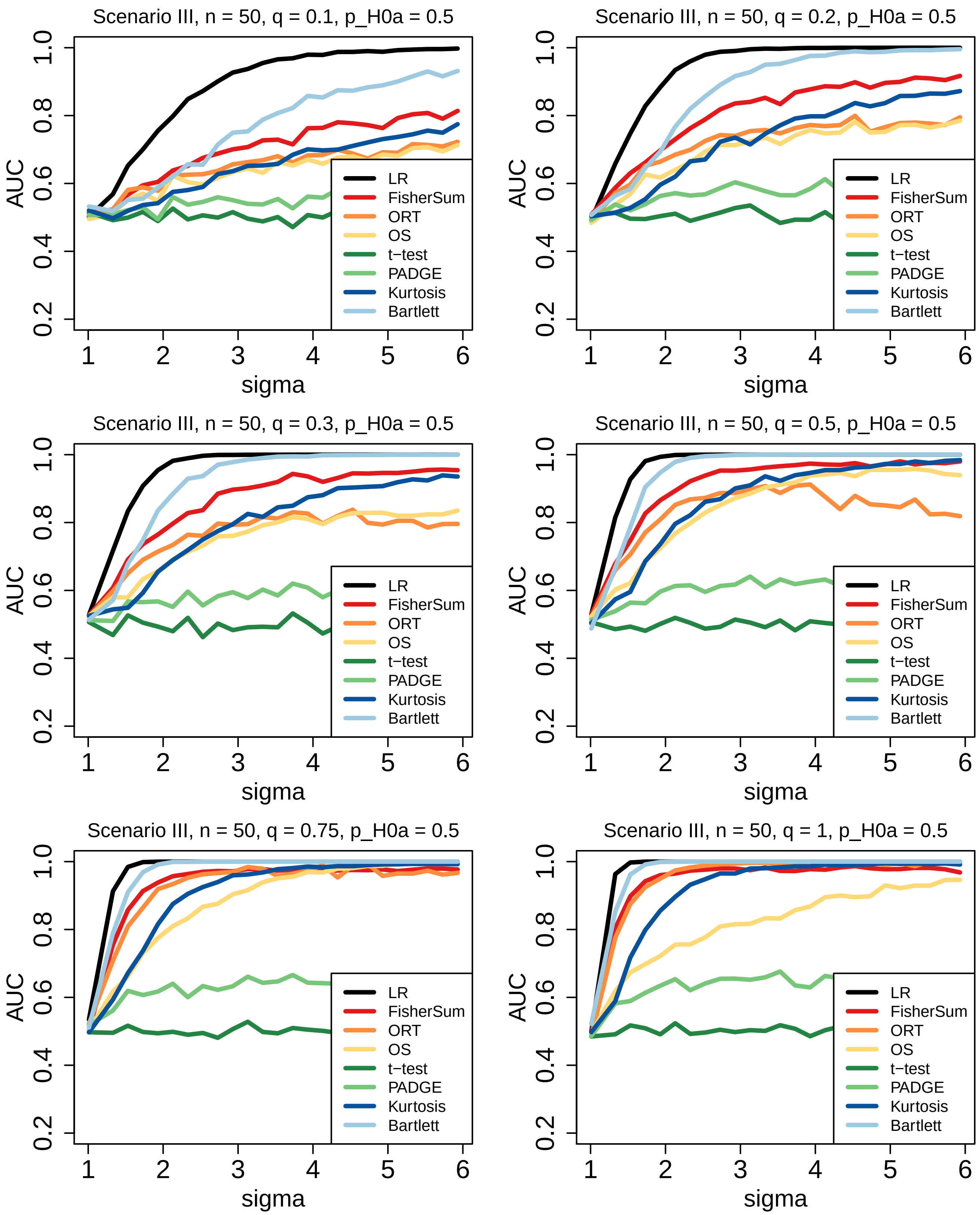
Scenario III, n = 50, q = 0.1, p_H0a = 0.5
Scenario III, n = 50, q = 0.2, p_H0a = 0.5
Scenario III, n = 50, q = 0.3, p_H0a = 0.5
Scenario III, n = 50, q = 0.5, p_H0a = 0.5
Scenario III, n = 50, q = 0.75, p_H0a = 0.5
Scenario III, n = 50, q = 1, p_H0a = 0.5
AUC
sigma
0.2
0.4
0.6
0.8
1.0
1
2
3
4
5
6
LR
FisherSum
ORT
OS
t−test
PADGE
Kurtosis
Bartlett

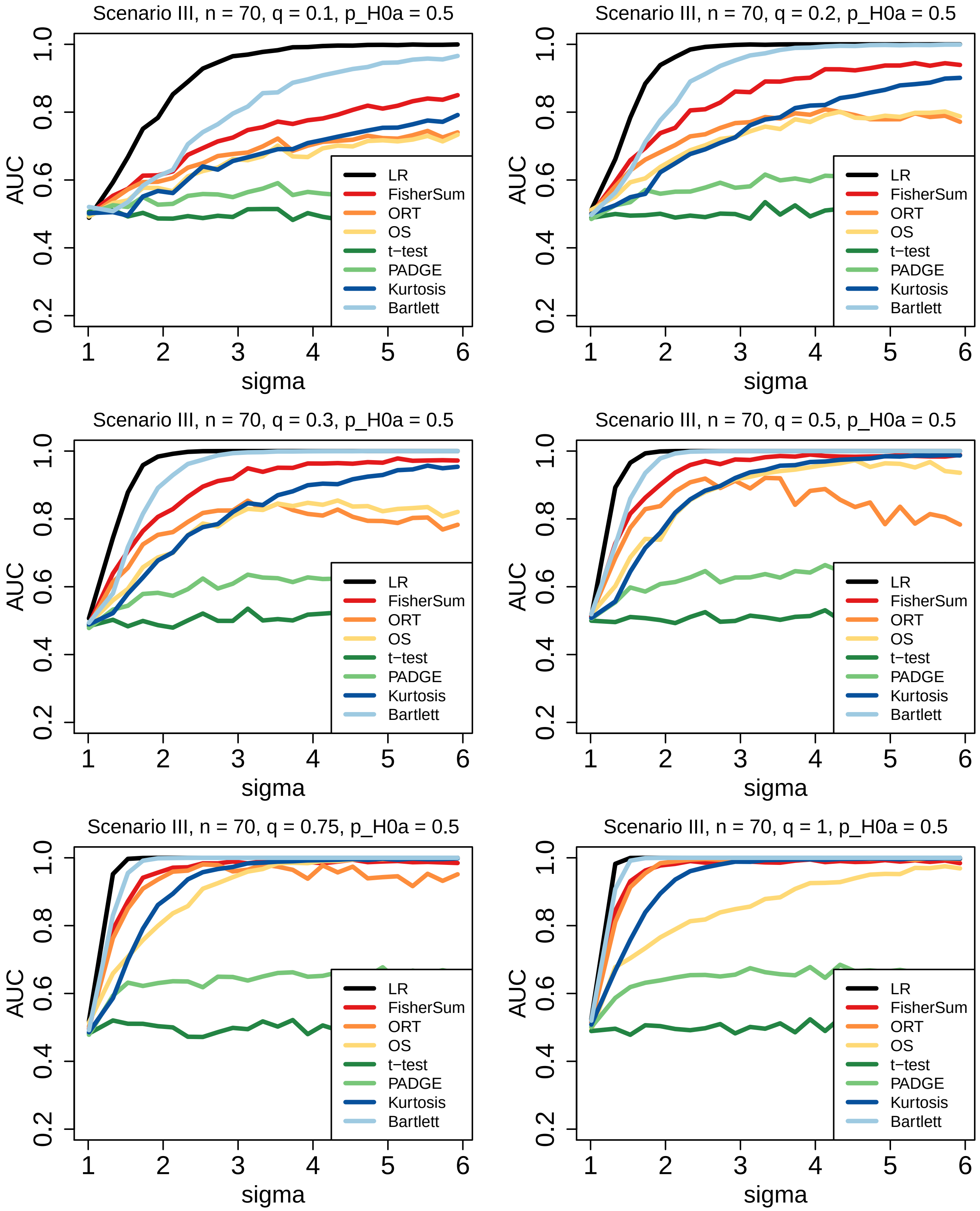
Scenario III, n = 70, q = 0.1, p_H0a = 0.5
Scenario III, n = 70, q = 0.2, p_H0a = 0.5
Scenario III, n = 70, q = 0.3, p_H0a = 0.5
Scenario III, n = 70, q = 0.5, p_H0a = 0.5
Scenario III, n = 70, q = 0.75, p_H0a = 0.5
Scenario III, n = 70, q = 1, p_H0a = 0.5
AUC
0.2
0.4
0.6
0.8
1.0
sigma
1
2
3
4
5
6
LR
FisherSum
ORT
OS
t−test
PADGE
Kurtosis
Bartlett

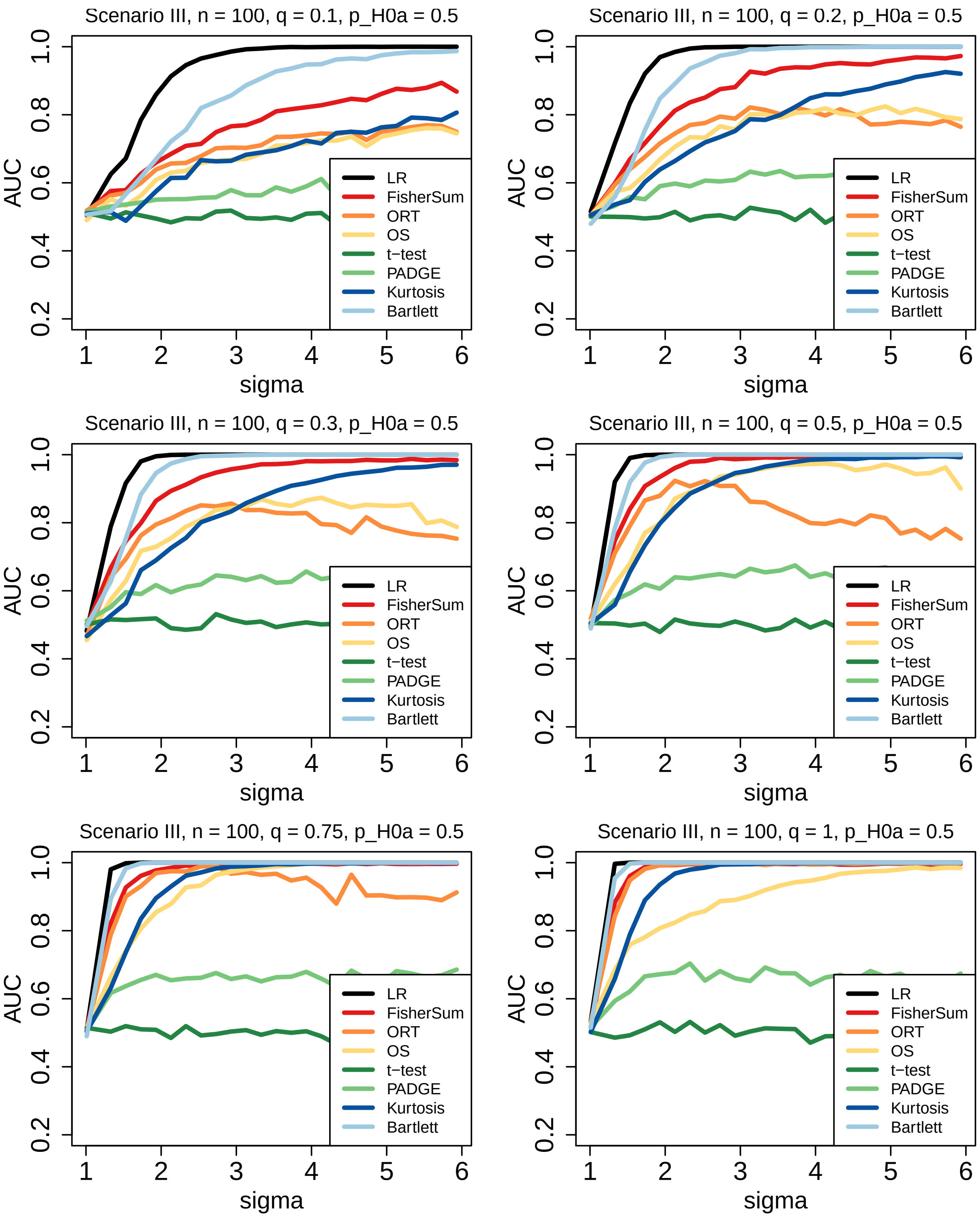

Figure S1. In-depth comparison of different methods' AUC values for the composite null situation. Included are all combinations of sample sizes n, subgroup proportions q, and scenarios s with $p_{H_{0a}} = 0.5$.

1.2 Simulation results (simple null situation)

Analogously to the study presented in section 1.1, we here show the results from a simulation with $p_{H_{0a}} = 1$. In this case, the different methods have to cope with distinctly smaller numbers of false positives among the null situation. Compared to $p_{H_{0a}} = 0.5$, ORT seems to benefit the most from this simplification. For more details, please refer to the main paper.
Table S4- S6 give summary of the best tests for each combination of sample size n and subgroup proportion q for the different scenarios, when the simple null situation is used. According to the simulation results for scenario I and II, FisherSum performs best for small to moderate sample sizes with subgroups up to $30 - 50$ percent. In case of larger samples, FisherSum yields best results for moderate deviations. Again, t-test performs best, for large values of $q \geq 0.5$ and for small deviations from the null situation resp., in scenario I and II. Results scenario III (bidirectional deregulation) suggest that generally, Kurtosis is appropriate in case of small subgroups and Bartlett's test for larger subgroup proportions.

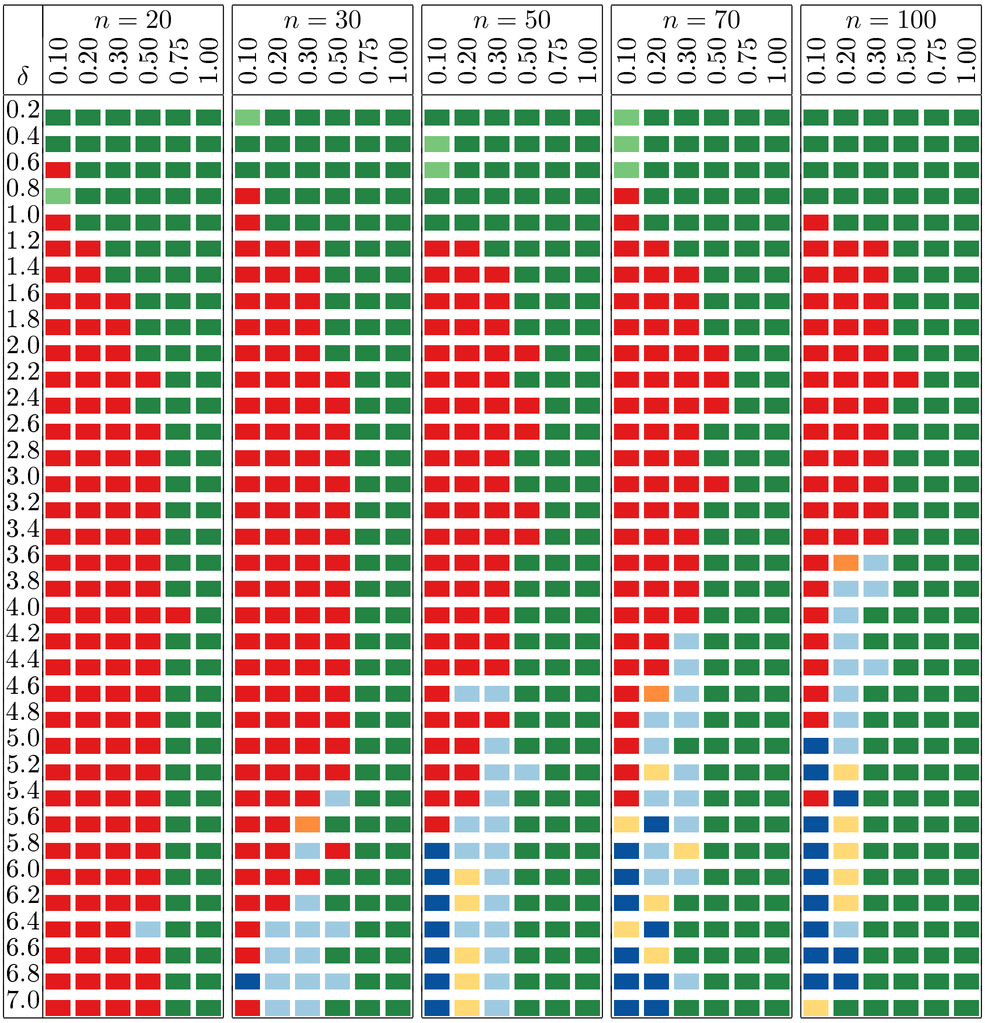

Table S4: Best tests for all combinations of sample size n and subgroup proportions $q = 0.1, 0.2, 0.3, 0.5, 0.75, 1$ in scenario I, $p_{H_{0a}} = 1$.
Color code: ■ FisherSum, ■ Bartlett's test, ■ t-test, ■ ORT, ■ Kurtosis, ■ OS, ■ PADGE.

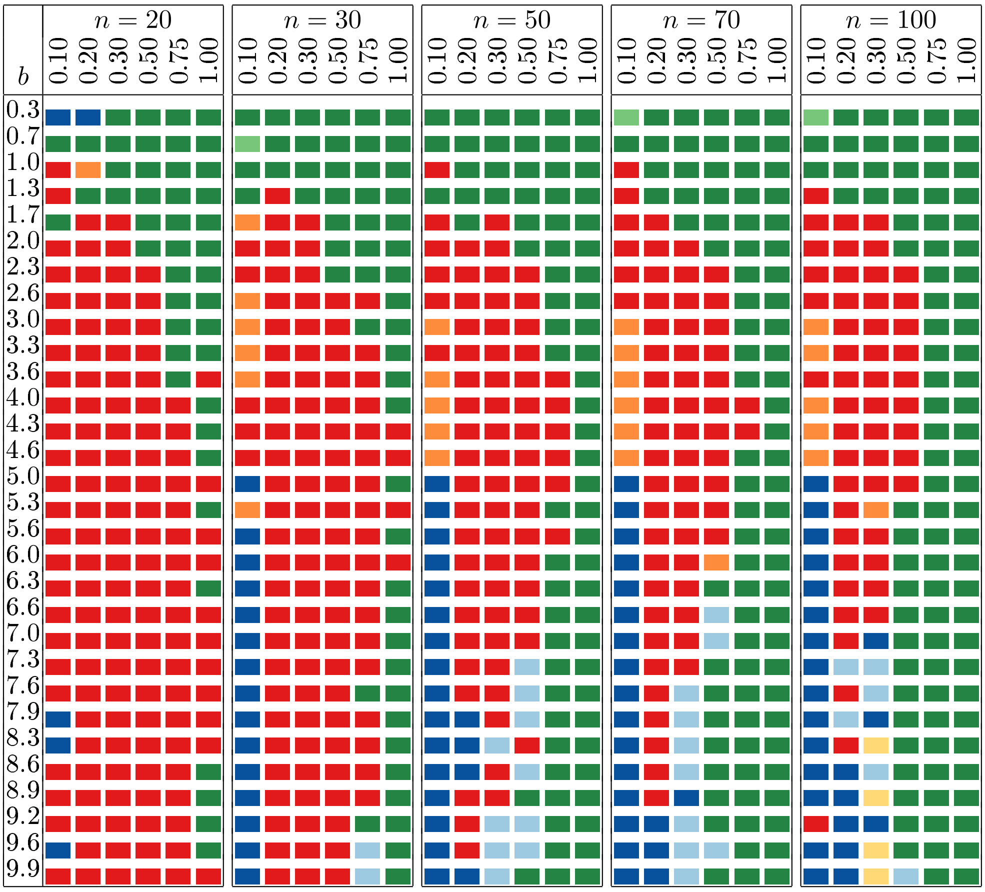

Table S5: Best tests for all combinations of sample size n and subgroup proportions $q = 0.1, 0.2, 0.3, 0.5, 0.75, 1$ in scenario II, $p_{H_{0a}} = 1$.
Color code: ■ FisherSum, ■ Bartlett's test, ■ t-test, ■ ORT, ■ Kurtosis, ■ OS, ■ PADGE.

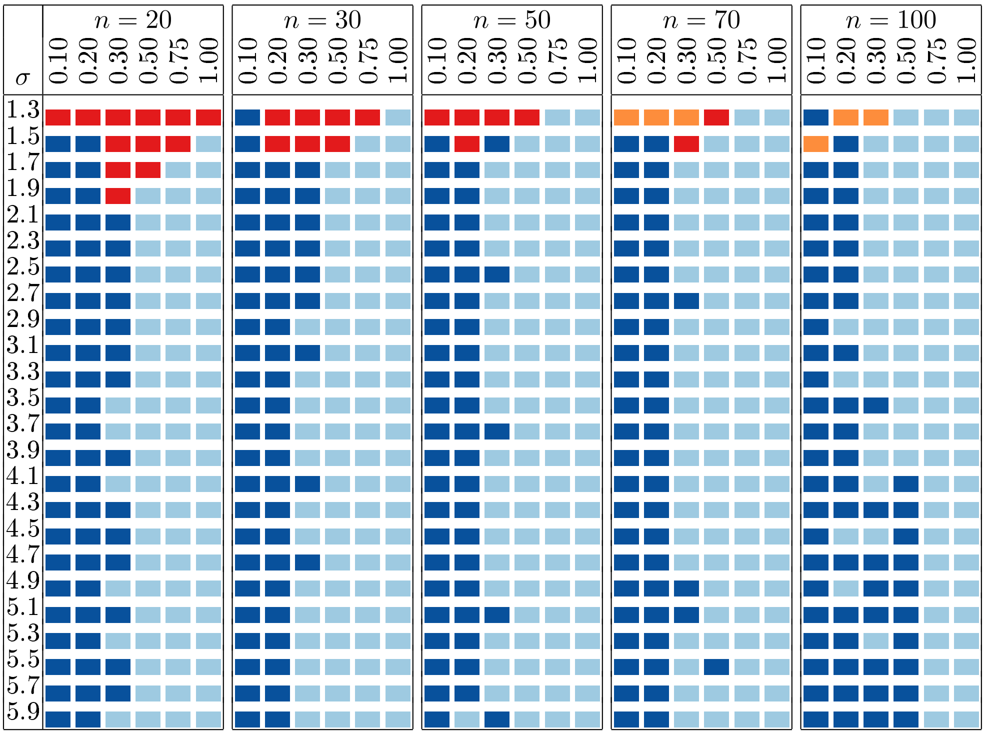

Table S6: Best tests for all combinations of sample size n and subgroup proportions $q = 0.1, 0.2, 0.3, 0.5, 0.75, 1$ in scenario III, $p_{H_{0a}} = 1$.
Color code: ■ FisherSum, ■ Bartlett's test, ■ t-test, ■ ORT, ■ Kurtosis, ■ OS, ■ PADGE.

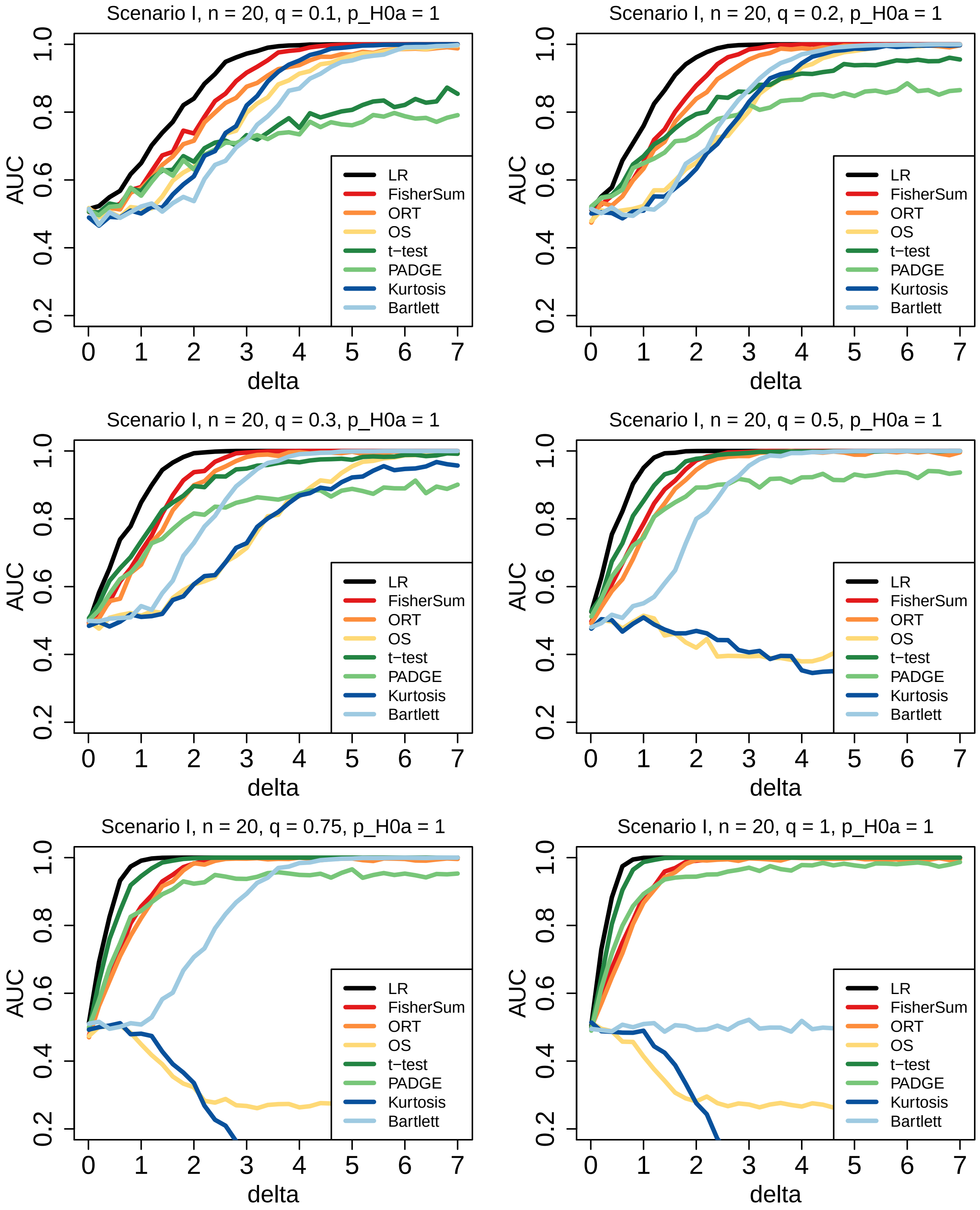
Scenario I, n = 20, q = 0.1, p_H0a = 1
Scenario I, n = 20, q = 0.2, p_H0a = 1
Scenario I, n = 20, q = 0.3, p_H0a = 1
Scenario I, n = 20, q = 0.5, p_H0a = 1
Scenario I, n = 20, q = 0.75, p_H0a = 1
Scenario I, n = 20, q = 1, p_H0a = 1
AUC
0.2
0.4
0.6
0.8
1.0
delta
0
1
2
3
4
5
6
7
LR
FisherSum
ORT
OS
t−test
PADGE
Kurtosis
Bartlett

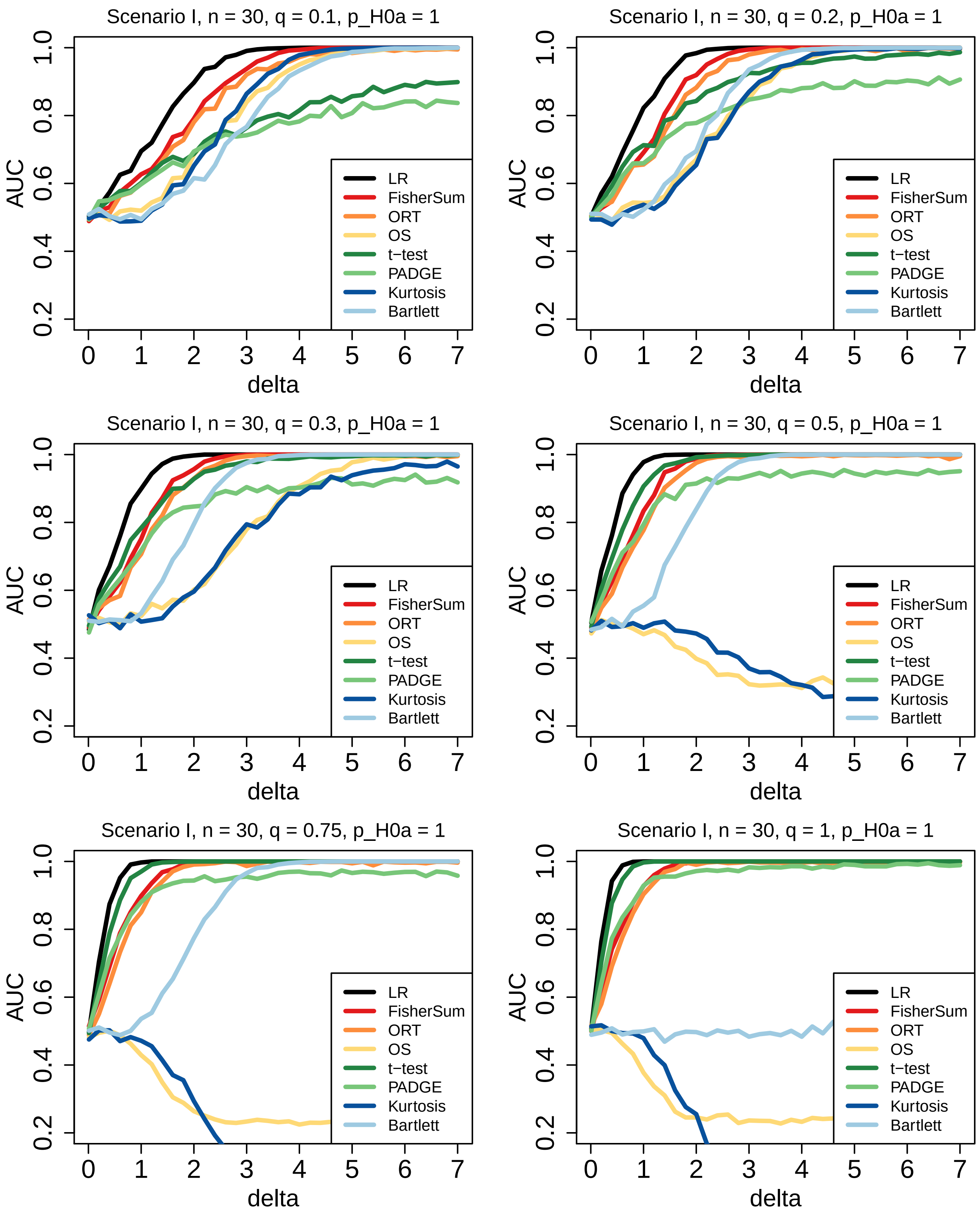
Scenario I, n = 30, q = 0.1, p_H0a = 1
Scenario I, n = 30, q = 0.2, p_H0a = 1
Scenario I, n = 30, q = 0.3, p_H0a = 1
Scenario I, n = 30, q = 0.5, p_H0a = 1
Scenario I, n = 30, q = 0.75, p_H0a = 1
Scenario I, n = 30, q = 1, p_H0a = 1
AUC
delta
0.2
0.4
0.6
0.8
1.0
0
1
2
3
4
5
6
7
LR
FisherSum
ORT
OS
t−test
PADGE
Kurtosis
Bartlett

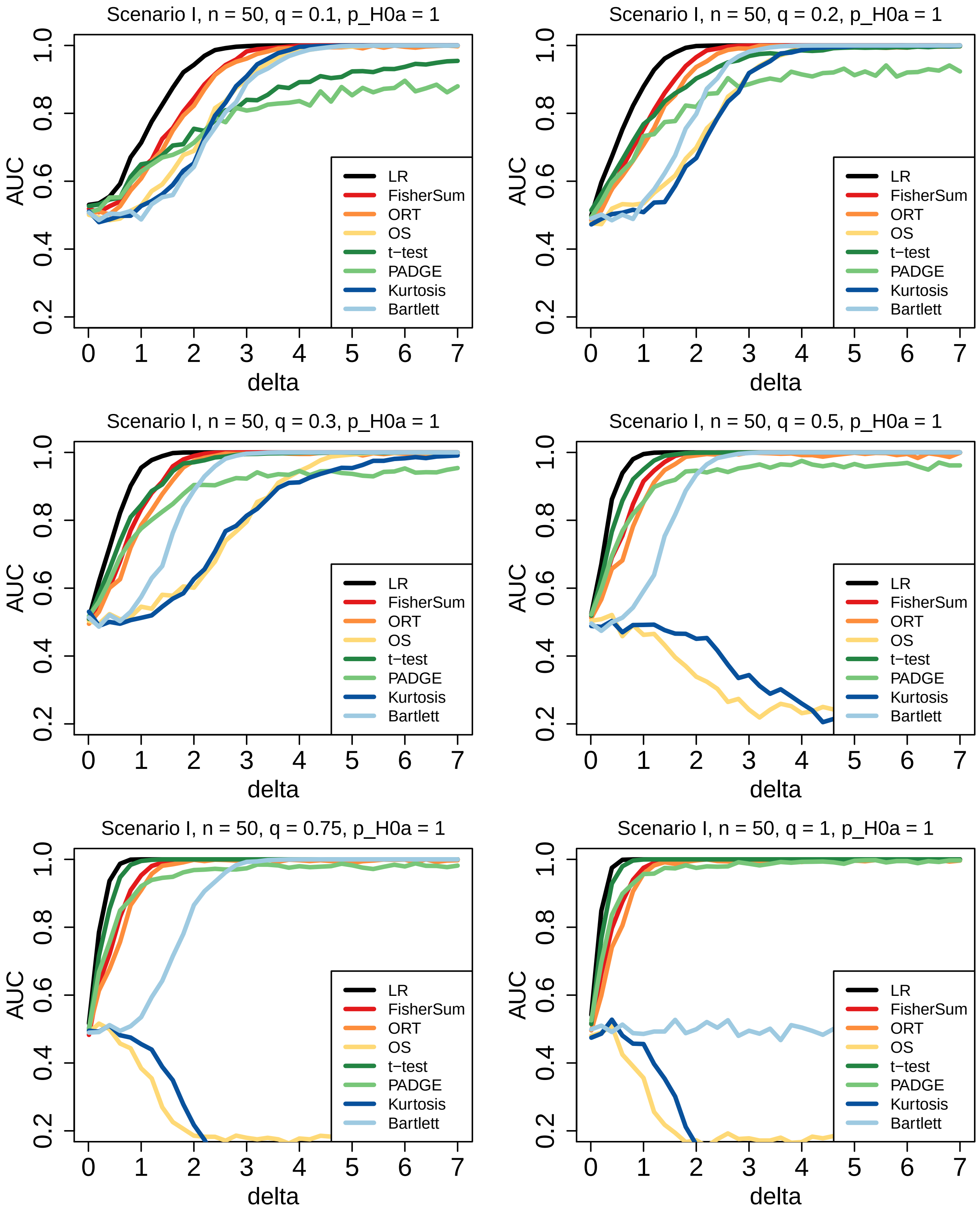
Scenario I, n = 50, q = 0.1, p_H0a = 1
Scenario I, n = 50, q = 0.2, p_H0a = 1
Scenario I, n = 50, q = 0.3, p_H0a = 1
Scenario I, n = 50, q = 0.5, p_H0a = 1
Scenario I, n = 50, q = 0.75, p_H0a = 1
Scenario I, n = 50, q = 1, p_H0a = 1
AUC
0.2
0.4
0.6
0.8
1.0
delta
0
1
2
3
4
5
6
7
LR
FisherSum
ORT
OS
t−test
PADGE
Kurtosis
Bartlett

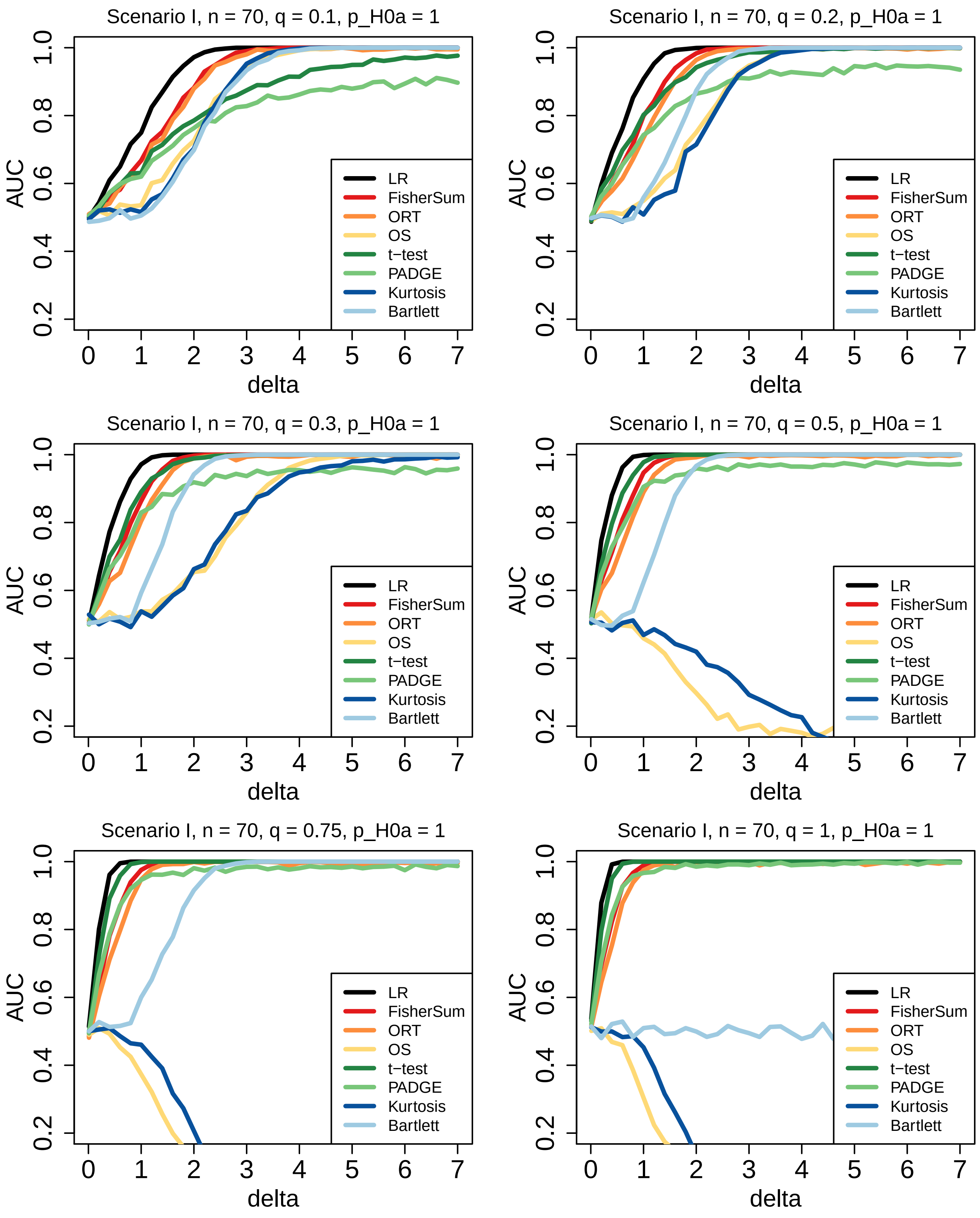
Scenario I, n = 70, q = 0.1, p_H0a = 1
Scenario I, n = 70, q = 0.2, p_H0a = 1
Scenario I, n = 70, q = 0.3, p_H0a = 1
Scenario I, n = 70, q = 0.5, p_H0a = 1
Scenario I, n = 70, q = 0.75, p_H0a = 1
Scenario I, n = 70, q = 1, p_H0a = 1
AUC
delta
LR
FisherSum
ORT
OS
t−test
PADGE
Kurtosis
Bartlett

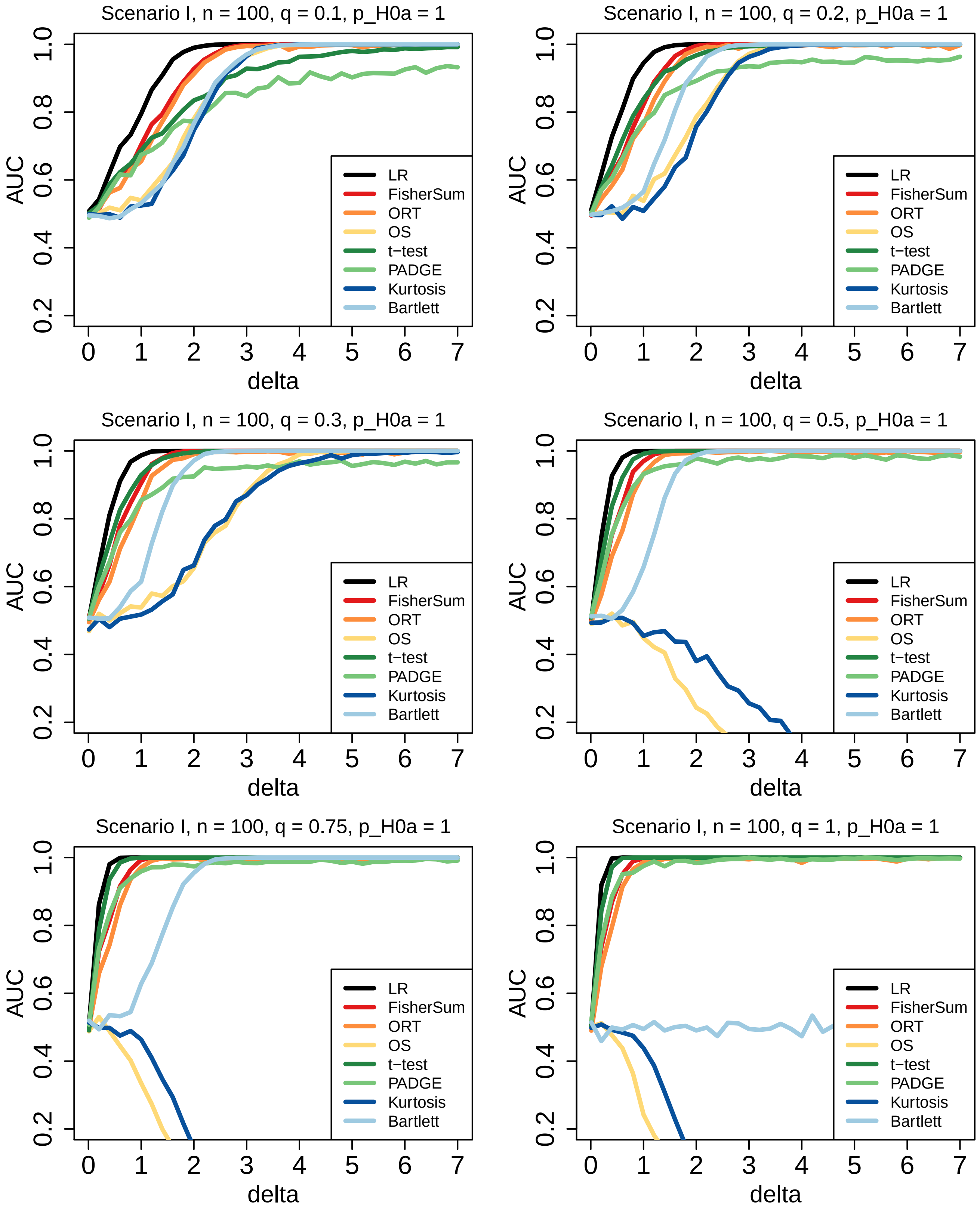
Scenario I, n = 100, q = 0.1, p_H0a = 1
Scenario I, n = 100, q = 0.2, p_H0a = 1
Scenario I, n = 100, q = 0.3, p_H0a = 1
Scenario I, n = 100, q = 0.5, p_H0a = 1
Scenario I, n = 100, q = 0.75, p_H0a = 1
Scenario I, n = 100, q = 1, p_H0a = 1
AUC
0.2
0.4
0.6
0.8
1.0
delta
0
1
2
3
4
5
6
7
LR
FisherSum
ORT
OS
t−test
PADGE
Kurtosis
Bartlett

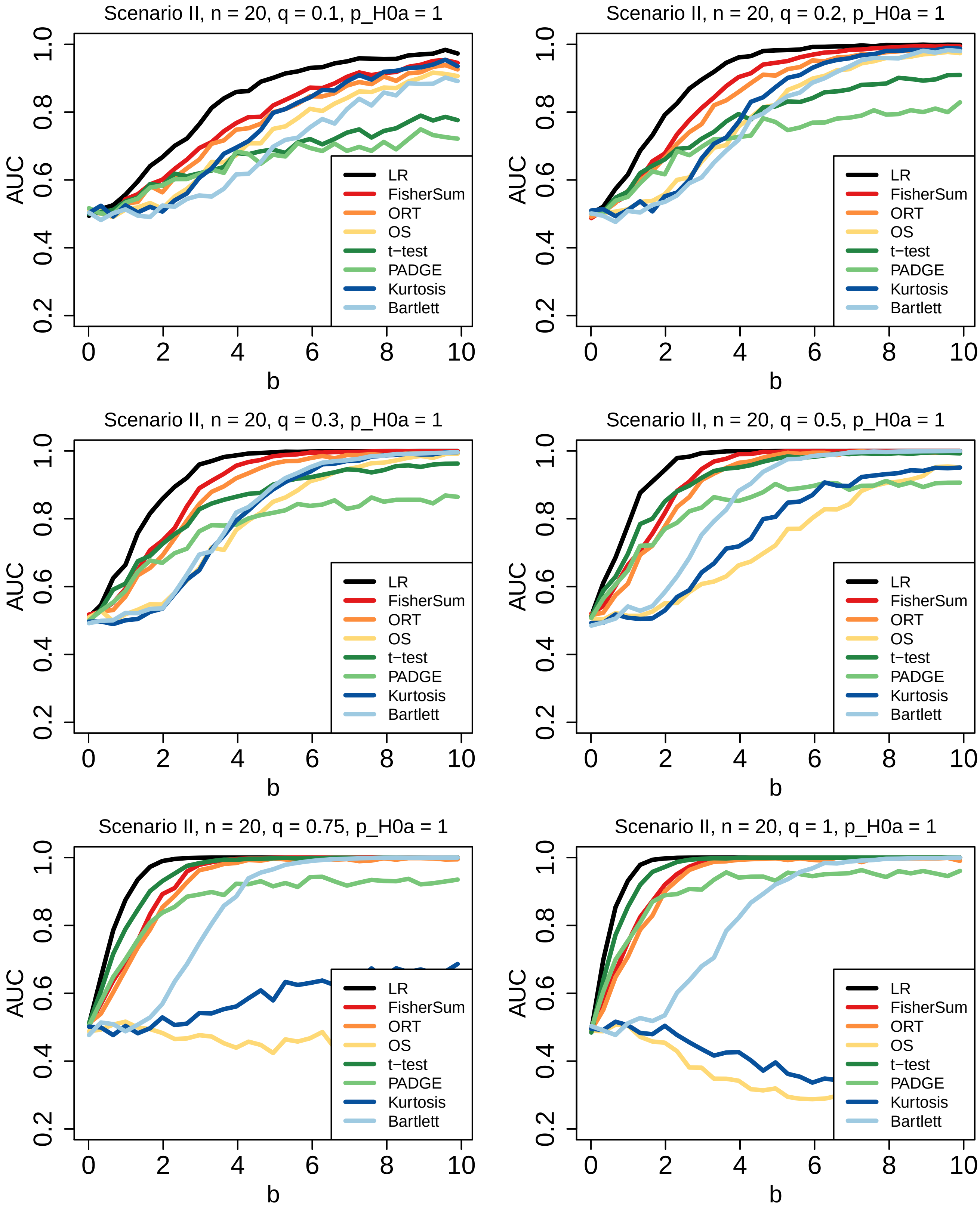
Scenario II, n = 20, q = 0.1, p_H0a = 1
Scenario II, n = 20, q = 0.2, p_H0a = 1
Scenario II, n = 20, q = 0.3, p_H0a = 1
Scenario II, n = 20, q = 0.5, p_H0a = 1
Scenario II, n = 20, q = 0.75, p_H0a = 1
Scenario II, n = 20, q = 1, p_H0a = 1
AUC
b
0.2
0.4
0.6
0.8
1.0
0
2
4
6
8
10
LR
FisherSum
ORT
OS
t−test
PADGE
Kurtosis
Bartlett

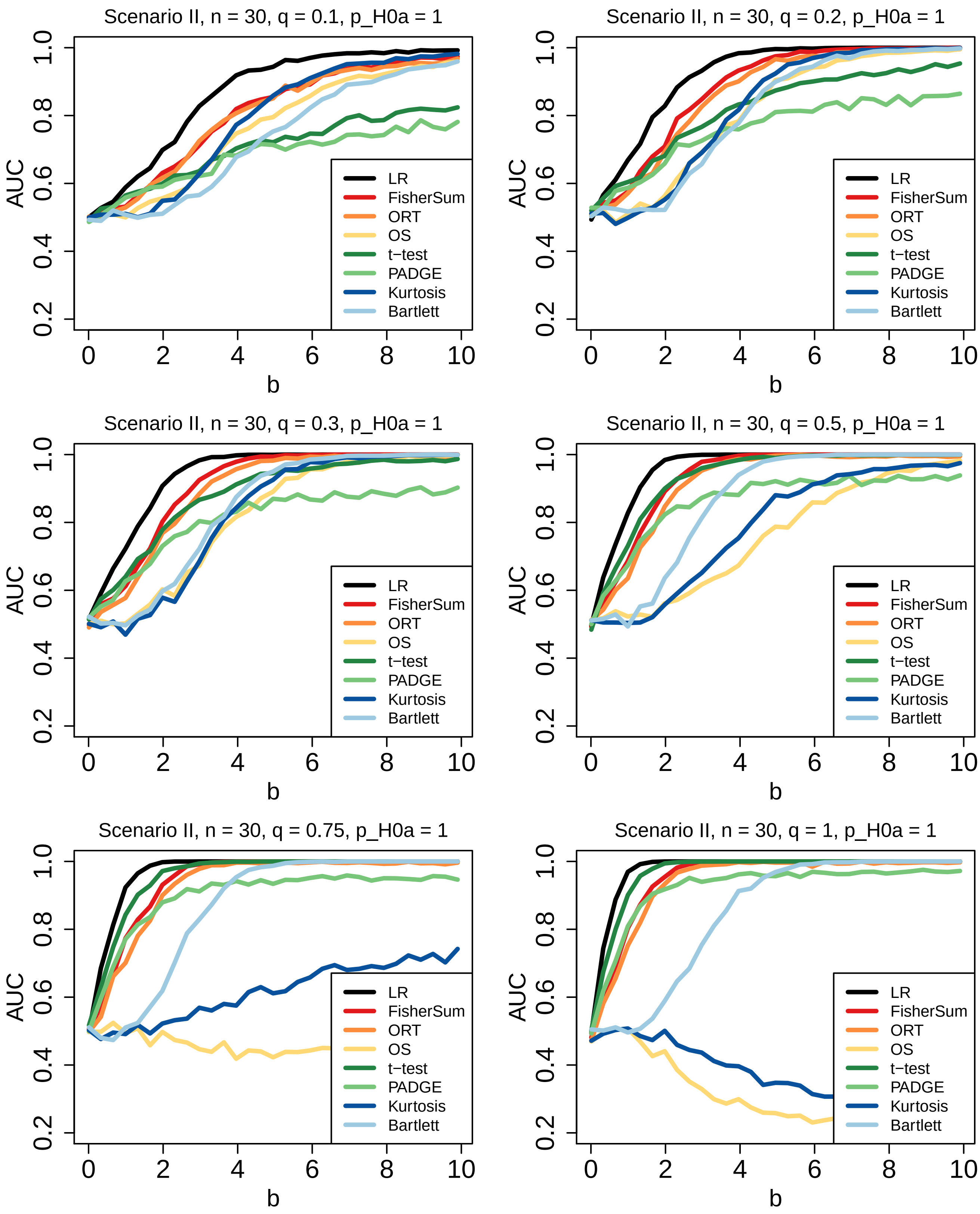

Scenario II, n = 30, q = 0.1, p_H0a = 1
Scenario II, n = 30, q = 0.2, p_H0a = 1
Scenario II, n = 30, q = 0.3, p_H0a = 1
Scenario II, n = 30, q = 0.5, p_H0a = 1
Scenario II, n = 30, q = 0.75, p_H0a = 1
Scenario II, n = 30, q = 1, p_H0a = 1
AUC
b
0.2
0.4
0.6
0.8
1.0
0
2
4
6
8
10
LR
FisherSum
ORT
OS
t−test
PADGE
Kurtosis
Bartlett

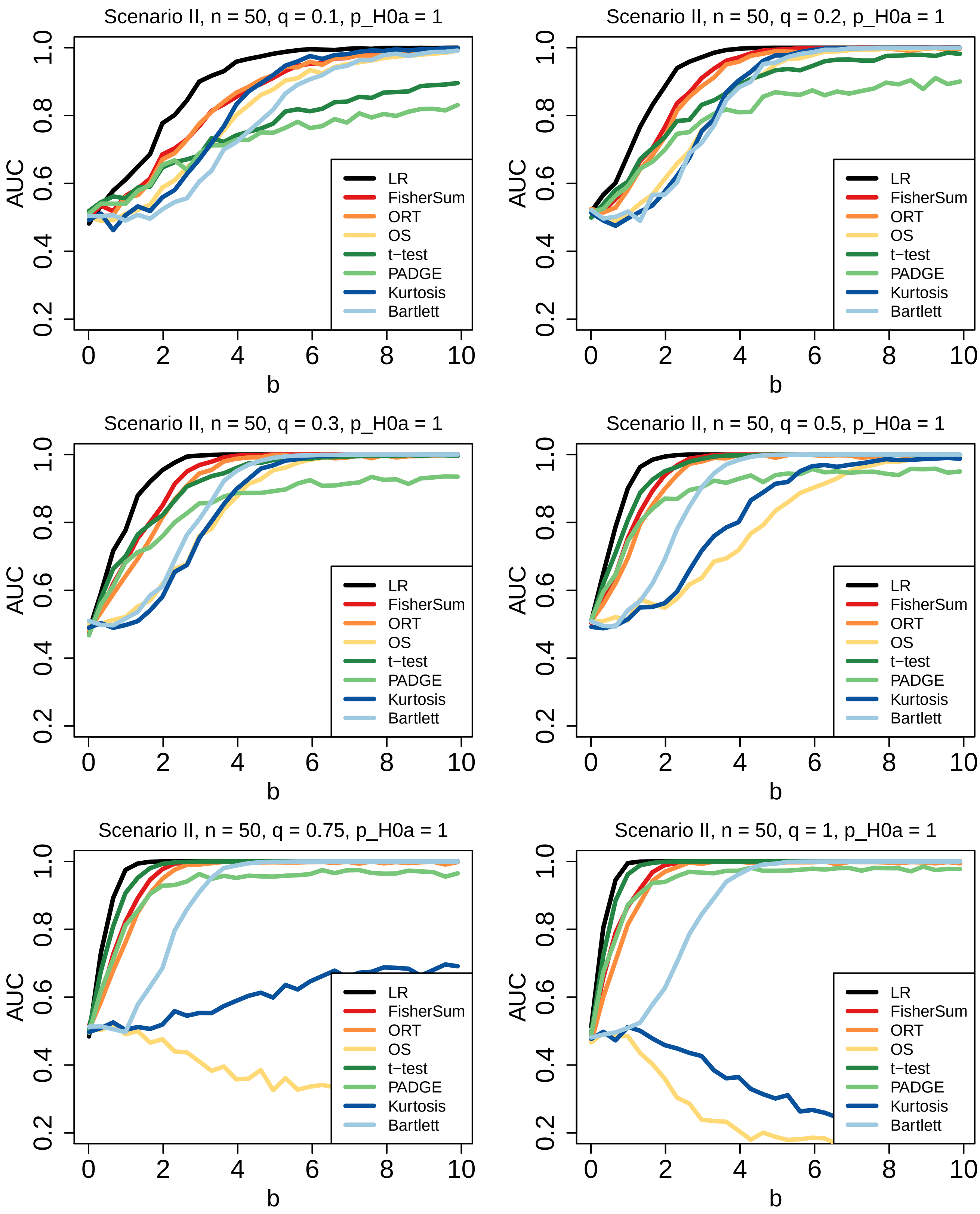
Scenario II, n = 50, q = 0.1, p_H0a = 1
Scenario II, n = 50, q = 0.2, p_H0a = 1
Scenario II, n = 50, q = 0.3, p_H0a = 1
Scenario II, n = 50, q = 0.5, p_H0a = 1
Scenario II, n = 50, q = 0.75, p_H0a = 1
Scenario II, n = 50, q = 1, p_H0a = 1
AUC
b
LR
FisherSum
ORT
OS
t−test
PADGE
Kurtosis
Bartlett

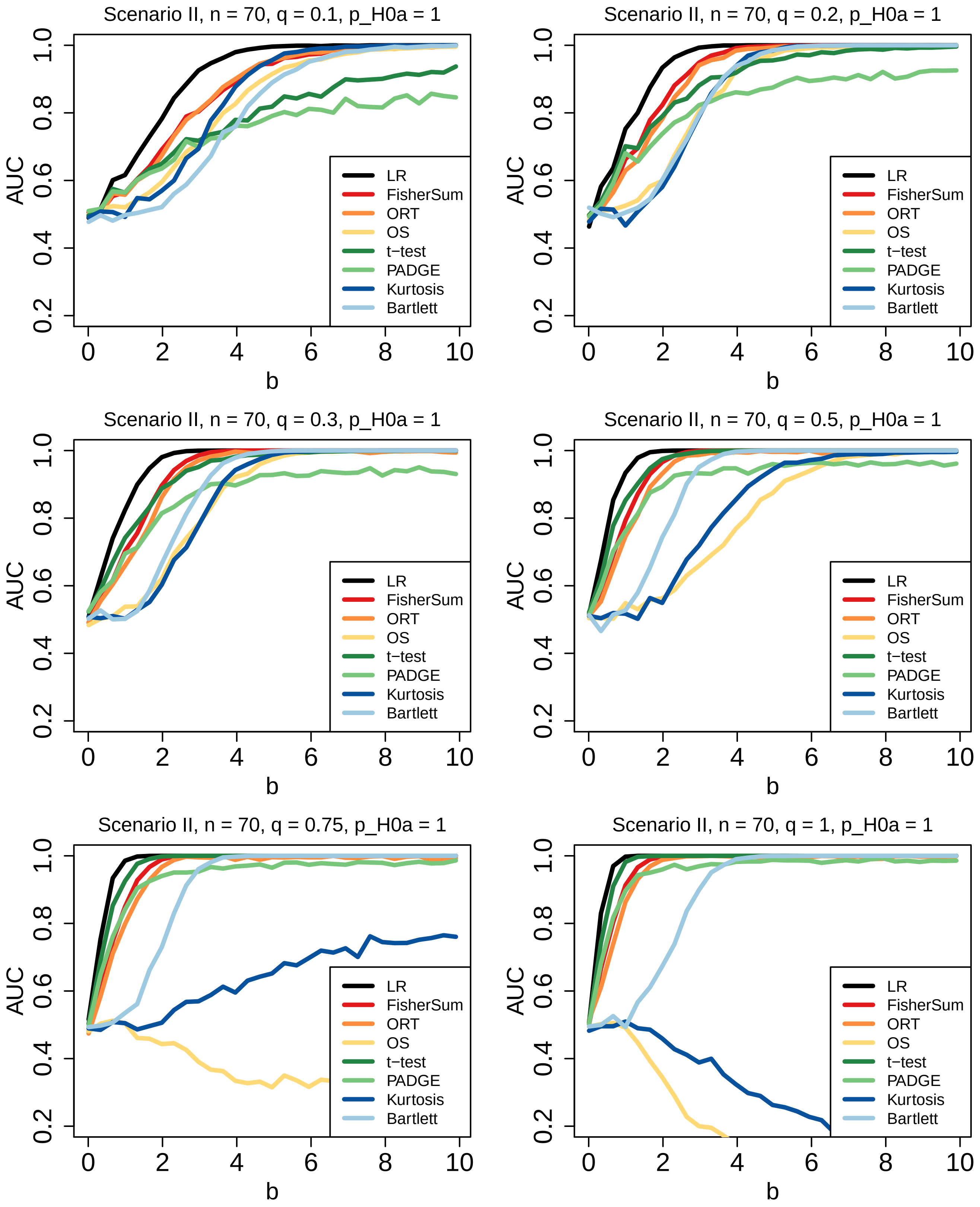
Scenario II, n = 70, q = 0.1, p_H0a = 1
Scenario II, n = 70, q = 0.2, p_H0a = 1
Scenario II, n = 70, q = 0.3, p_H0a = 1
Scenario II, n = 70, q = 0.5, p_H0a = 1
Scenario II, n = 70, q = 0.75, p_H0a = 1
Scenario II, n = 70, q = 1, p_H0a = 1
AUC
b
0.2
0.4
0.6
0.8
1.0
0
2
4
6
8
10
LR
FisherSum
ORT
OS
t−test
PADGE
Kurtosis
Bartlett

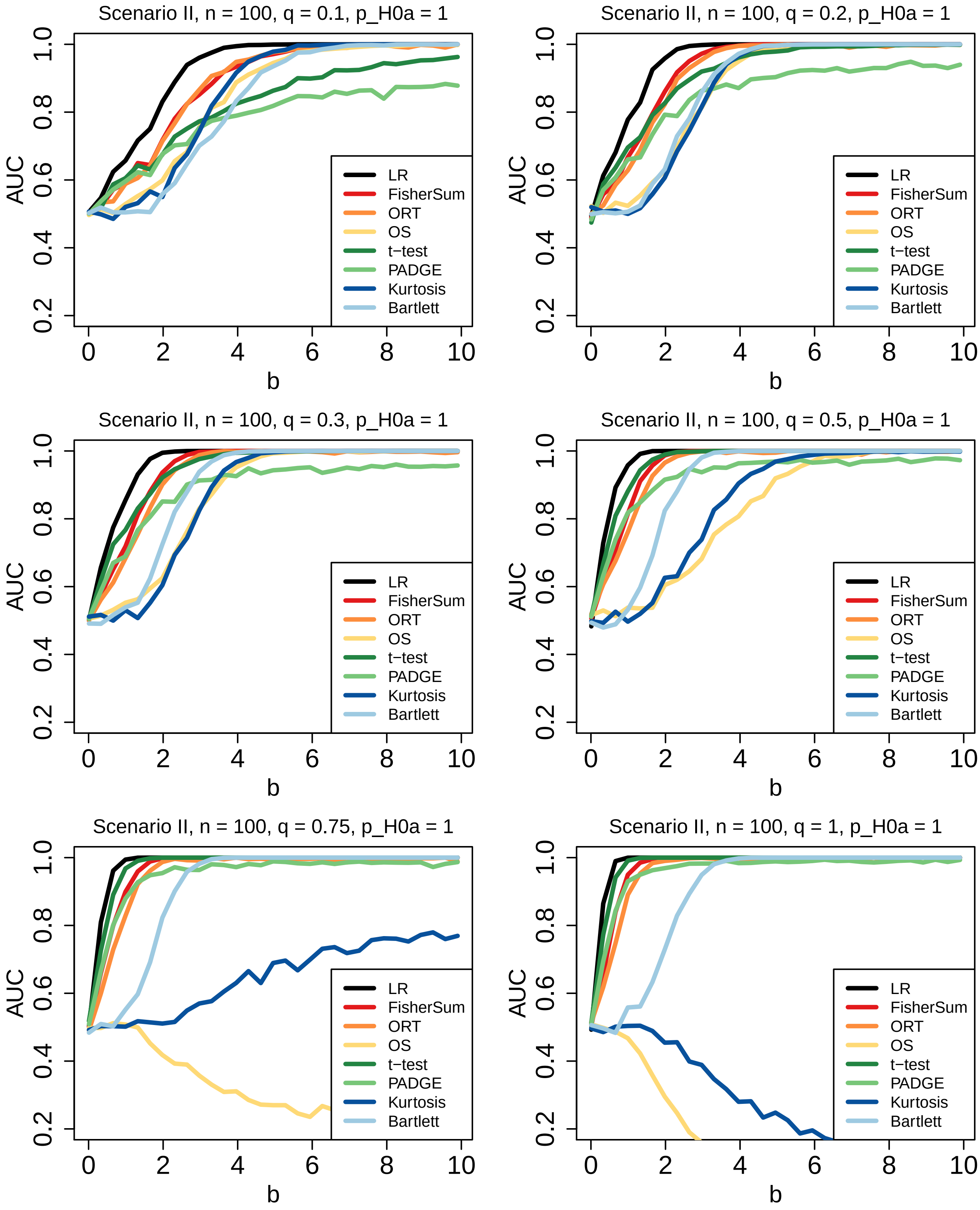
Scenario II, n = 100, q = 0.1, p_H0a = 1
Scenario II, n = 100, q = 0.2, p_H0a = 1
Scenario II, n = 100, q = 0.3, p_H0a = 1
Scenario II, n = 100, q = 0.5, p_H0a = 1
Scenario II, n = 100, q = 0.75, p_H0a = 1
Scenario II, n = 100, q = 1, p_H0a = 1
AUC
0.2
0.4
0.6
0.8
1.0
b
0
2
4
6
8
10
LR
FisherSum
ORT
OS
t−test
PADGE
Kurtosis
Bartlett

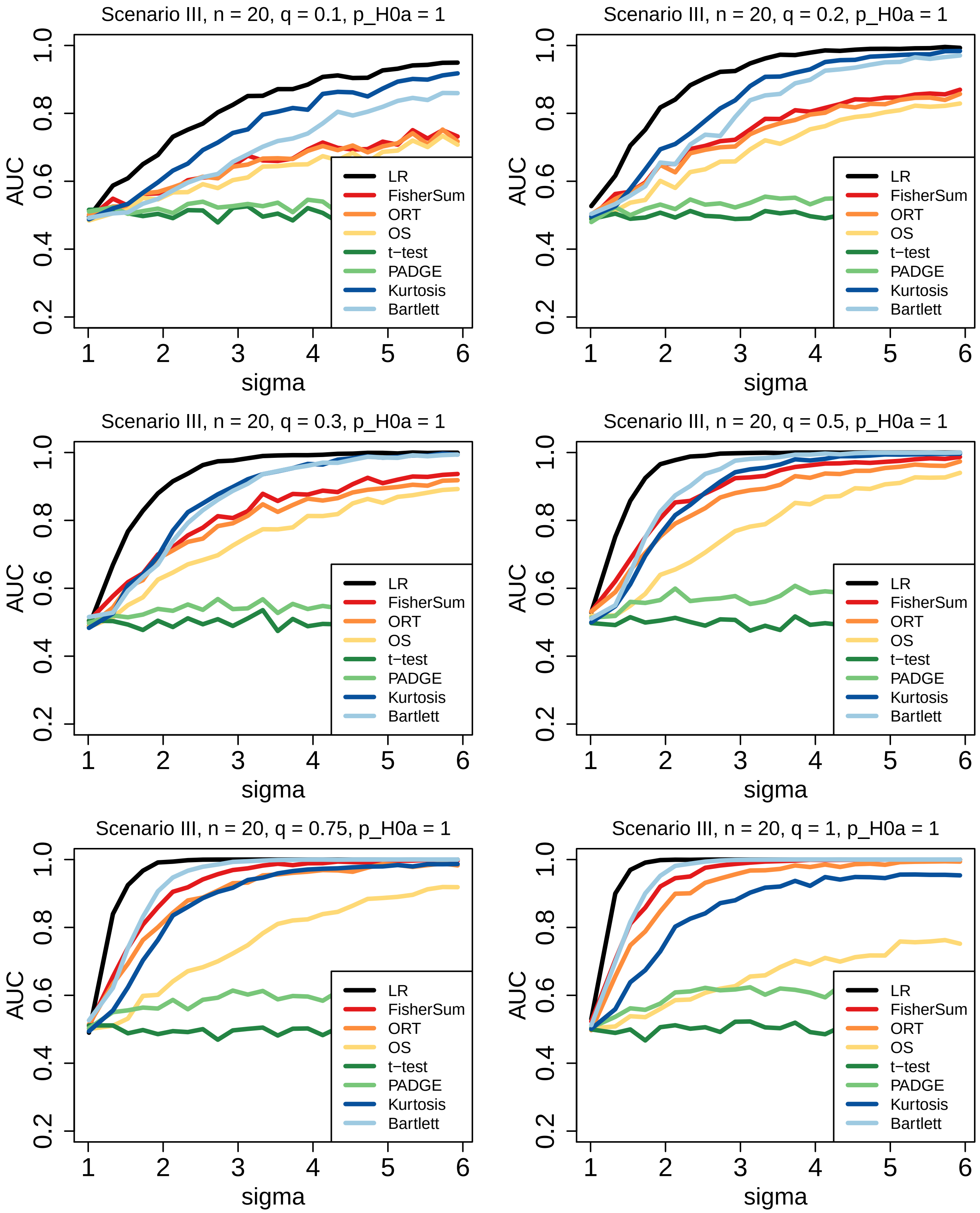

Scenario III, n = 20, q = 0.1, p_H0a = 1
Scenario III, n = 20, q = 0.2, p_H0a = 1
Scenario III, n = 20, q = 0.3, p_H0a = 1
Scenario III, n = 20, q = 0.5, p_H0a = 1
Scenario III, n = 20, q = 0.75, p_H0a = 1
Scenario III, n = 20, q = 1, p_H0a = 1
AUC
sigma
0.2
0.4
0.6
0.8
1.0
1
2
3
4
5
6
LR
FisherSum
ORT
OS
t−test
PADGE
Kurtosis
Bartlett

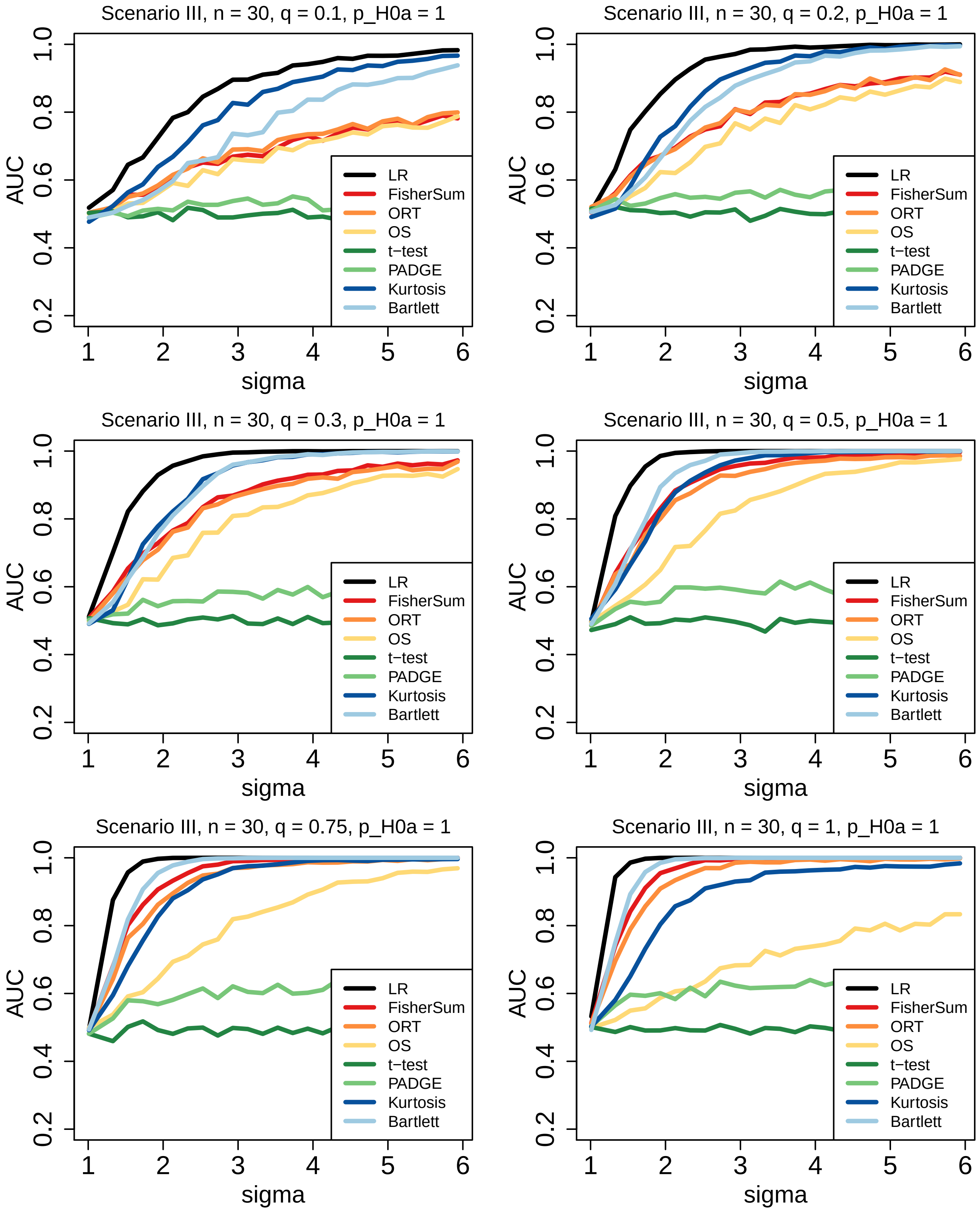
Scenario III, n = 30, q = 0.1, p_H0a = 1
Scenario III, n = 30, q = 0.2, p_H0a = 1
Scenario III, n = 30, q = 0.3, p_H0a = 1
Scenario III, n = 30, q = 0.5, p_H0a = 1
Scenario III, n = 30, q = 0.75, p_H0a = 1
Scenario III, n = 30, q = 1, p_H0a = 1
AUC
0.2
0.4
0.6
0.8
1.0
sigma
1
2
3
4
5
6
LR
FisherSum
ORT
OS
t−test
PADGE
Kurtosis
Bartlett

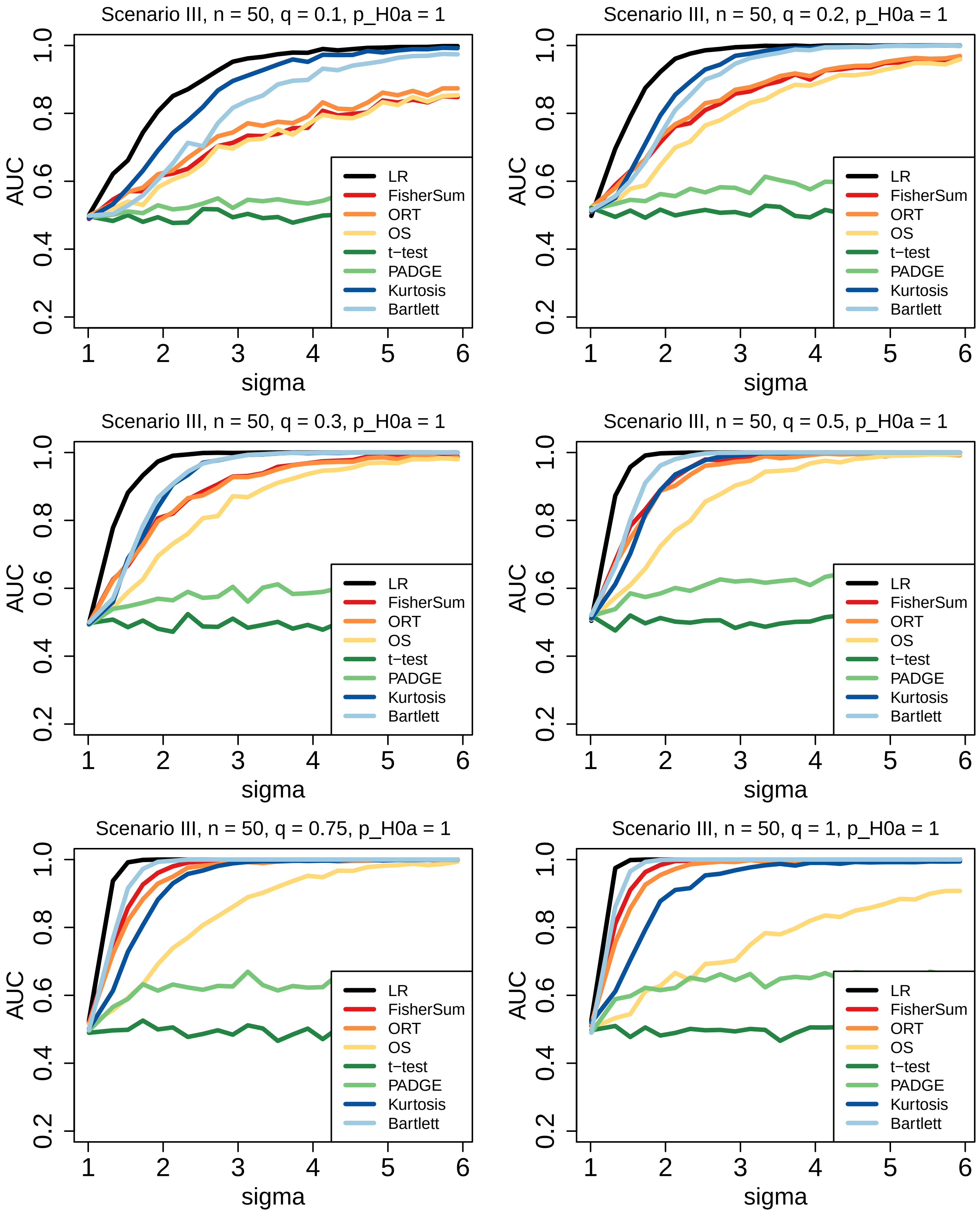
Scenario III, n = 50, q = 0.1, p_H0a = 1
Scenario III, n = 50, q = 0.2, p_H0a = 1
Scenario III, n = 50, q = 0.3, p_H0a = 1
Scenario III, n = 50, q = 0.5, p_H0a = 1
Scenario III, n = 50, q = 0.75, p_H0a = 1
Scenario III, n = 50, q = 1, p_H0a = 1
AUC
0.2
0.4
0.6
0.8
1.0
sigma
1
2
3
4
5
6
LR
FisherSum
ORT
OS
t−test
PADGE
Kurtosis
Bartlett

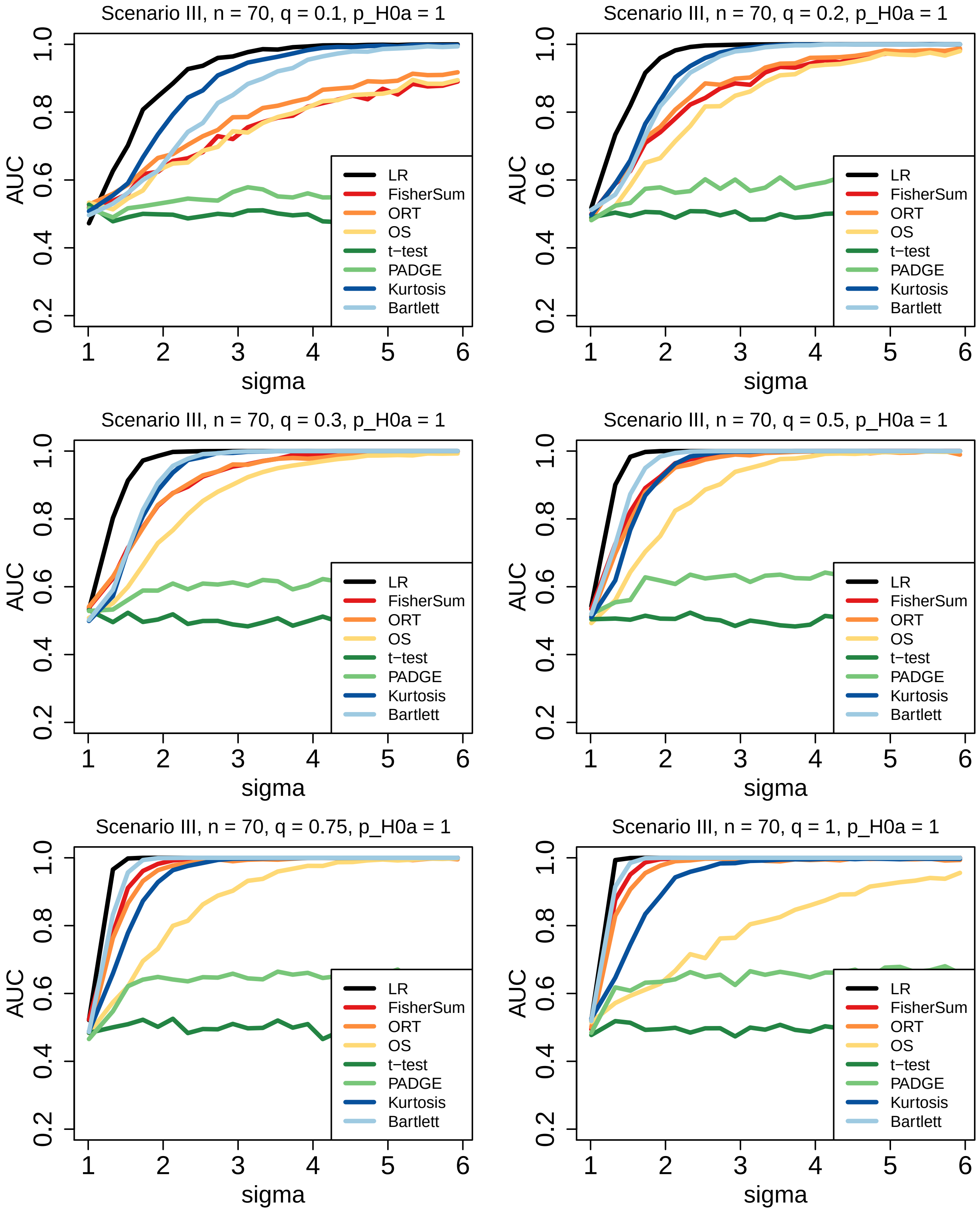

Scenario III, n = 70, q = 0.1, p_H0a = 1
Scenario III, n = 70, q = 0.2, p_H0a = 1
Scenario III, n = 70, q = 0.3, p_H0a = 1
Scenario III, n = 70, q = 0.5, p_H0a = 1
Scenario III, n = 70, q = 0.75, p_H0a = 1
Scenario III, n = 70, q = 1, p_H0a = 1
AUC
0.2
0.4
0.6
0.8
1.0
sigma
1
2
3
4
5
6
LR
FisherSum
ORT
OS
t−test
PADGE
Kurtosis
Bartlett

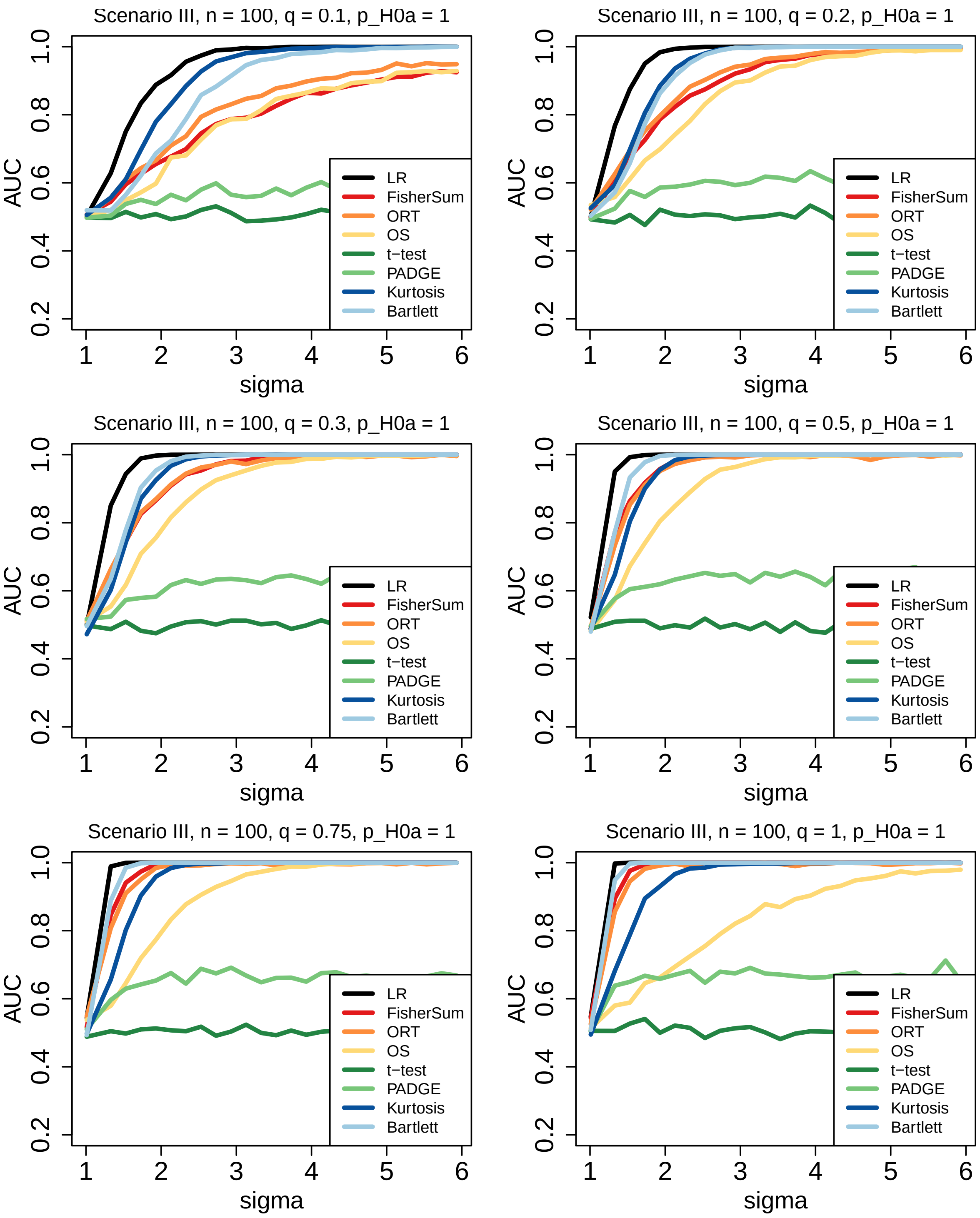

Figure S2. In-depth comparison of different methods' AUC values for the simple null situation. Included are all combinations of sample sizes n, subgroup proportions q, and scenarios s with $p_{H_{0a}} = 1$.

1.3 Top candidates of different measures for ParkCHIP data

On the following pages, we present ($\log_2$-)intensity plots of the top candidates of each method. In the ParkCHIP project more than two groups were analysed. The data set used for the following analyses is available at
`www.medizinisches-proteom-center.de/Ahrens_et_al`.

Observations on the left-hand side of each plot represent the control group (healthy controls, HC), and the right-hand side corresponds to patients with Parkinson's disease (PD). Observations per group are in random but fixed order across the variables. The header of each plot gives the Database ID corresponding to the depicted spot. For reasons of comparability of the characteristic distributional patterns of the top candidates, we used the same scale for all plots.

Here are some notes to summarize the distributional patterns of each method's top candidates:

FisherSum most top candidates show desired distributional pattern

ORT, OS mainly non-disease-specific subgroups (esp. ORT), detect slight to large increases

t-test detects global shifts as expected and apparently subgroups with slight to moderate up-regulation (distinct shift in means while variance remains similar)

PADGE some good candidates, some candidates do not show obvious subgroup patterns

Kurtosis most top candidates show single outliers

Bartlett single outliers and few potential subgroup candidates

Fisher10 50 variables yield the minimal p-value of 0.014, among them some with pretty slight up-regulation that seem to be false positive results, but some promising ones as well

To avoid any differences in the ranking of ORT and OS due to simulated p-values, we ranked the variables according to the actual test statistics. This approach also reduces the number of ties. In case of Fisher10, ties are non-avoidable. For simplicity, we chose the first 15 variables in the data sheet corresponding to the microarray that yield the minimal p-value.

Top candidates: FisherSum

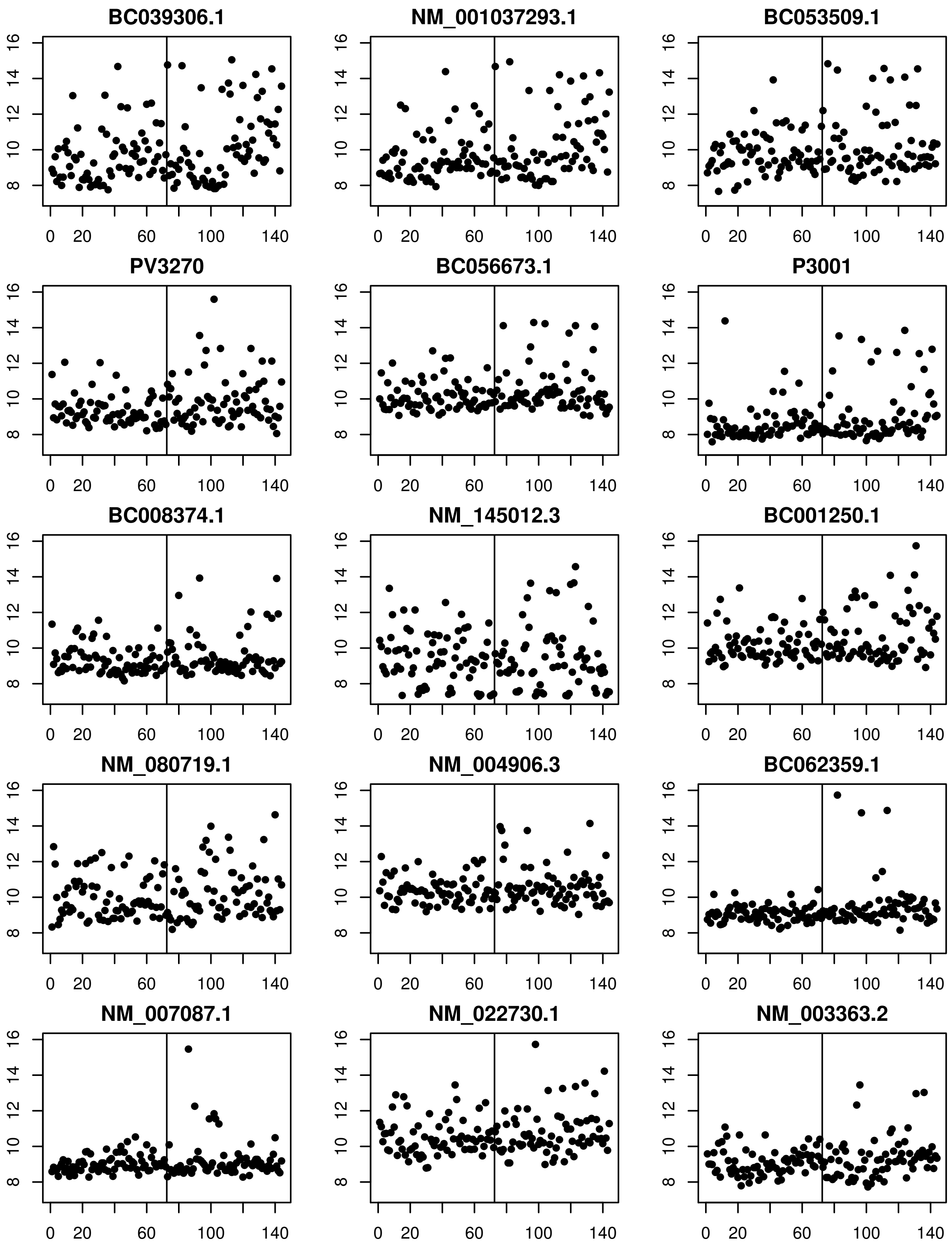

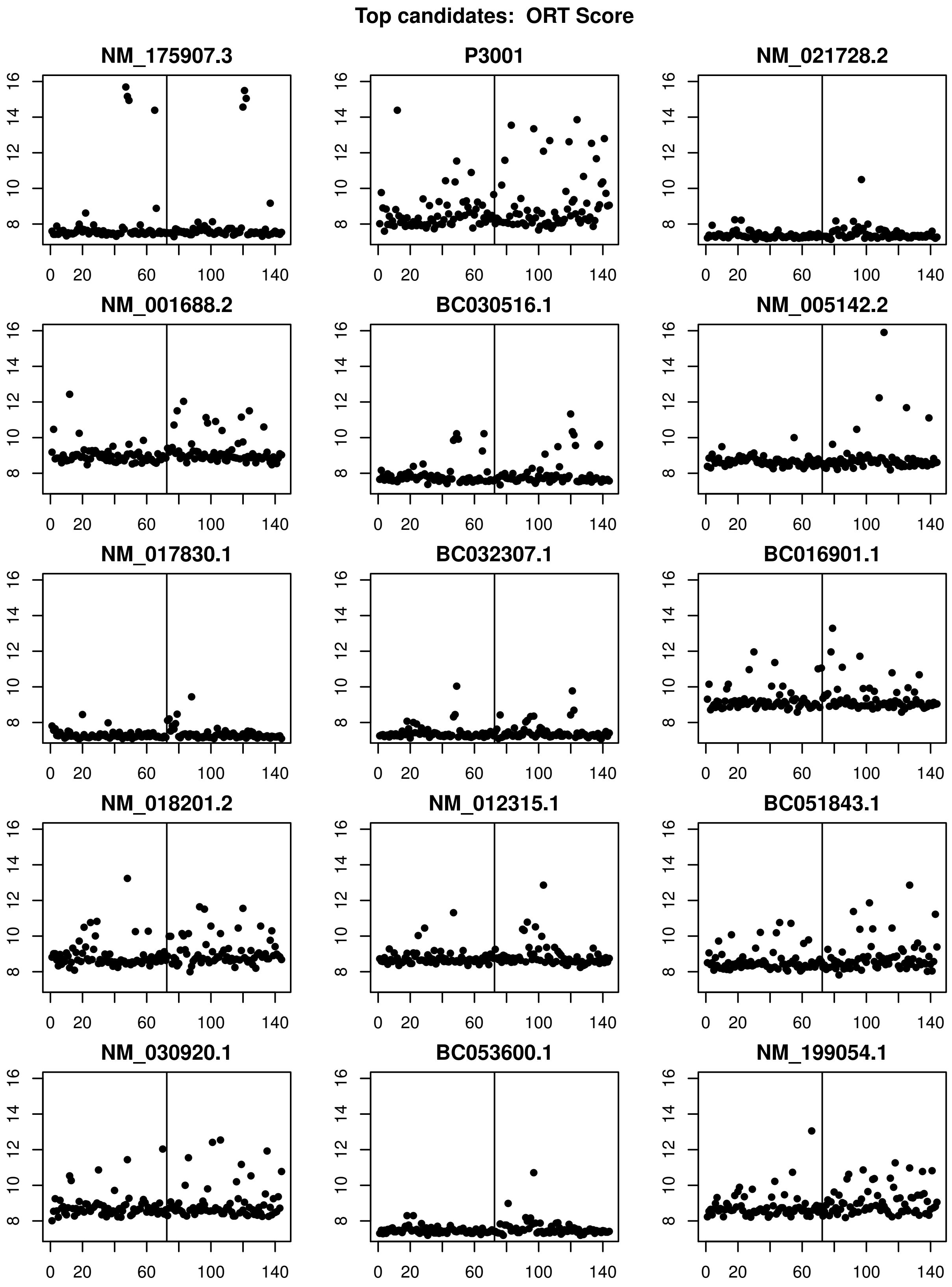
Top candidates: ORT Score
NM_175907.3
P3001
NM_021728.2
NM_001688.2
BC030516.1
NM_005142.2
NM_017830.1
BC032307.1
BC016901.1
NM_018201.2
NM_012315.1
BC051843.1
NM_030920.1
BC053600.1
NM_199054.1

Top candidates: OS Score

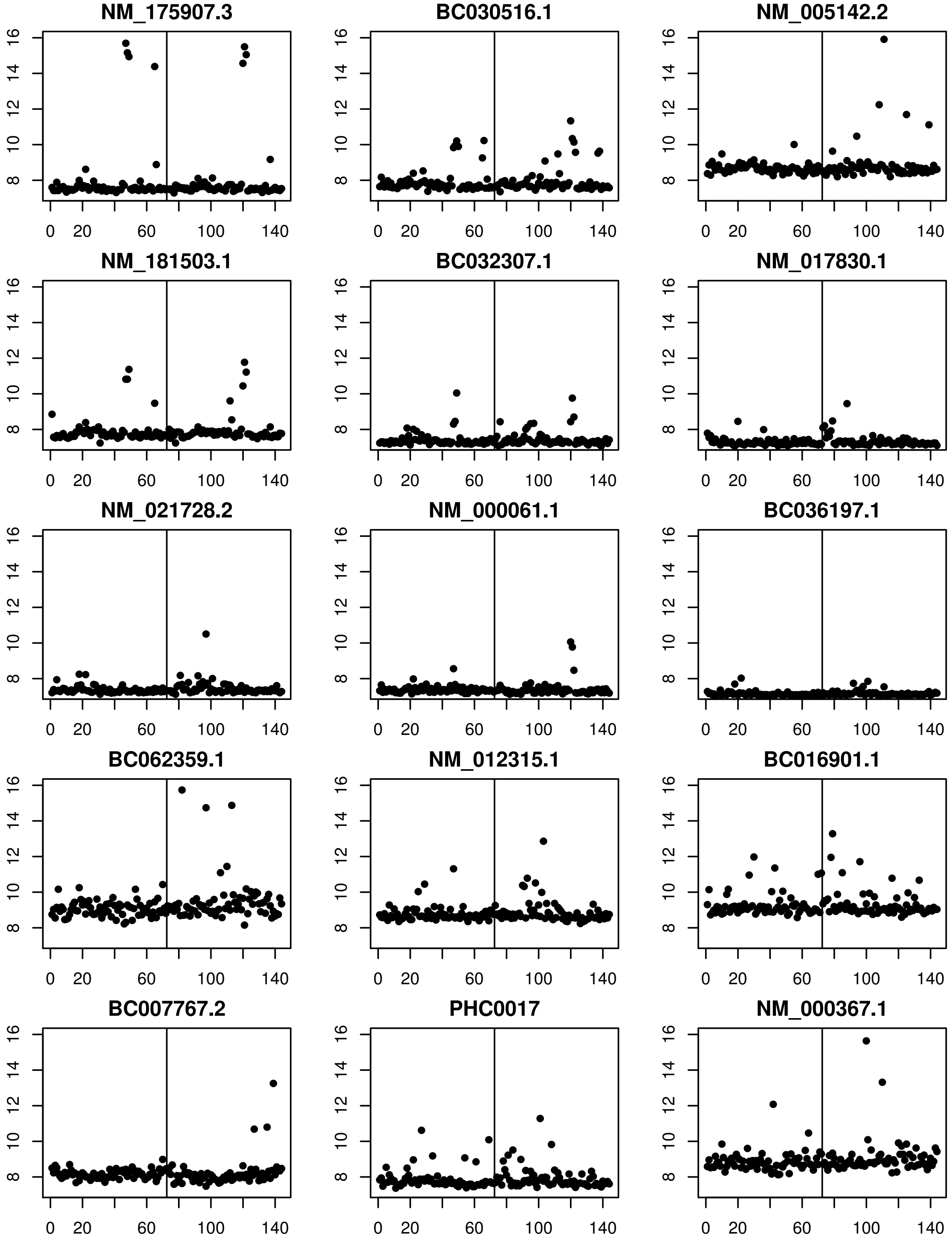

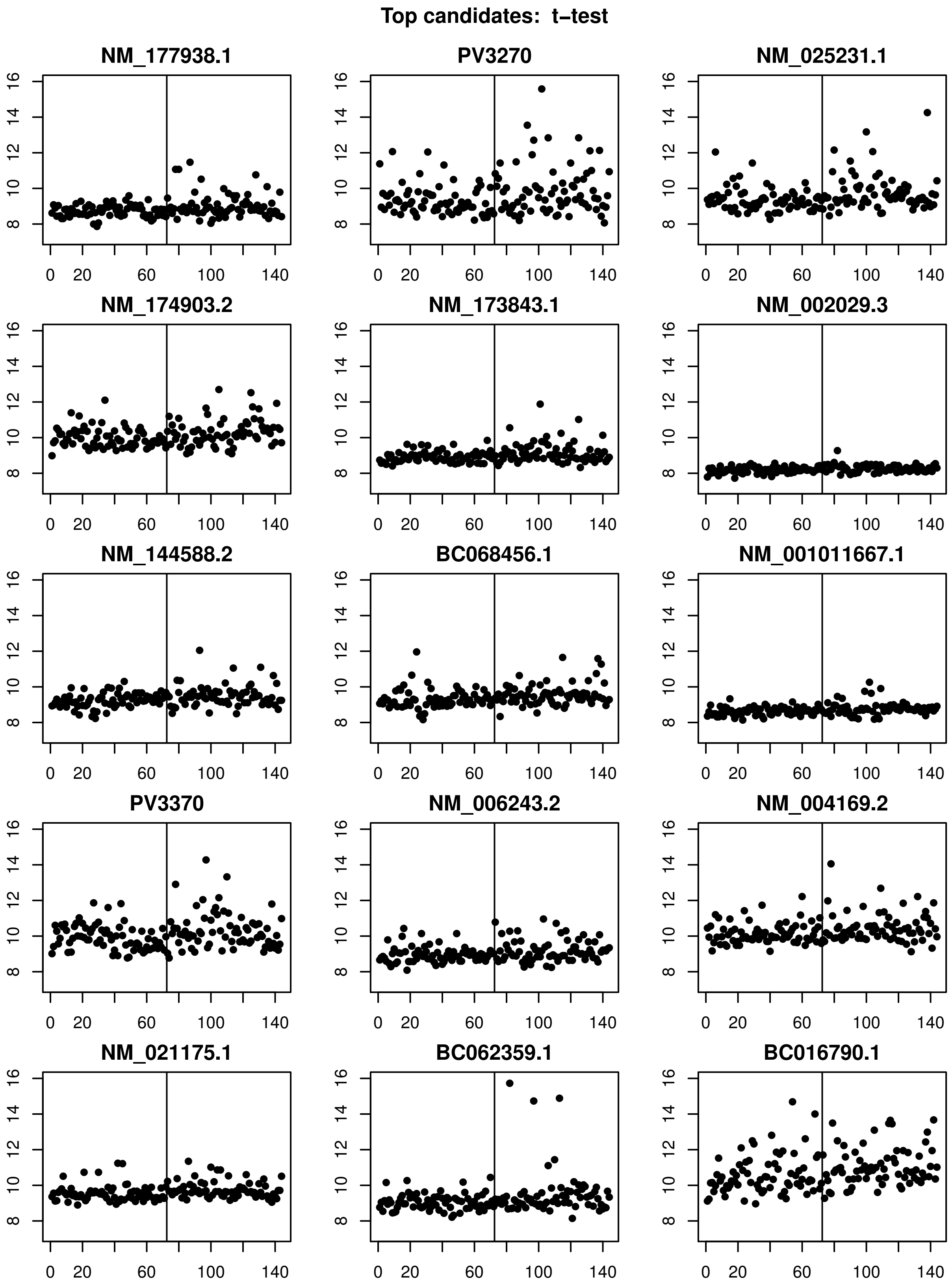
Top candidates: t−test
NM_177938.1
PV3270
NM_025231.1
NM_174903.2
NM_173843.1
NM_002029.3
NM_144588.2
BC068456.1
NM_001011667.1
PV3370
NM_006243.2
NM_004169.2
NM_021175.1
BC062359.1
BC016790.1
8
10
12
14
16
0
20
60
100
140

Top candidates: PADGE

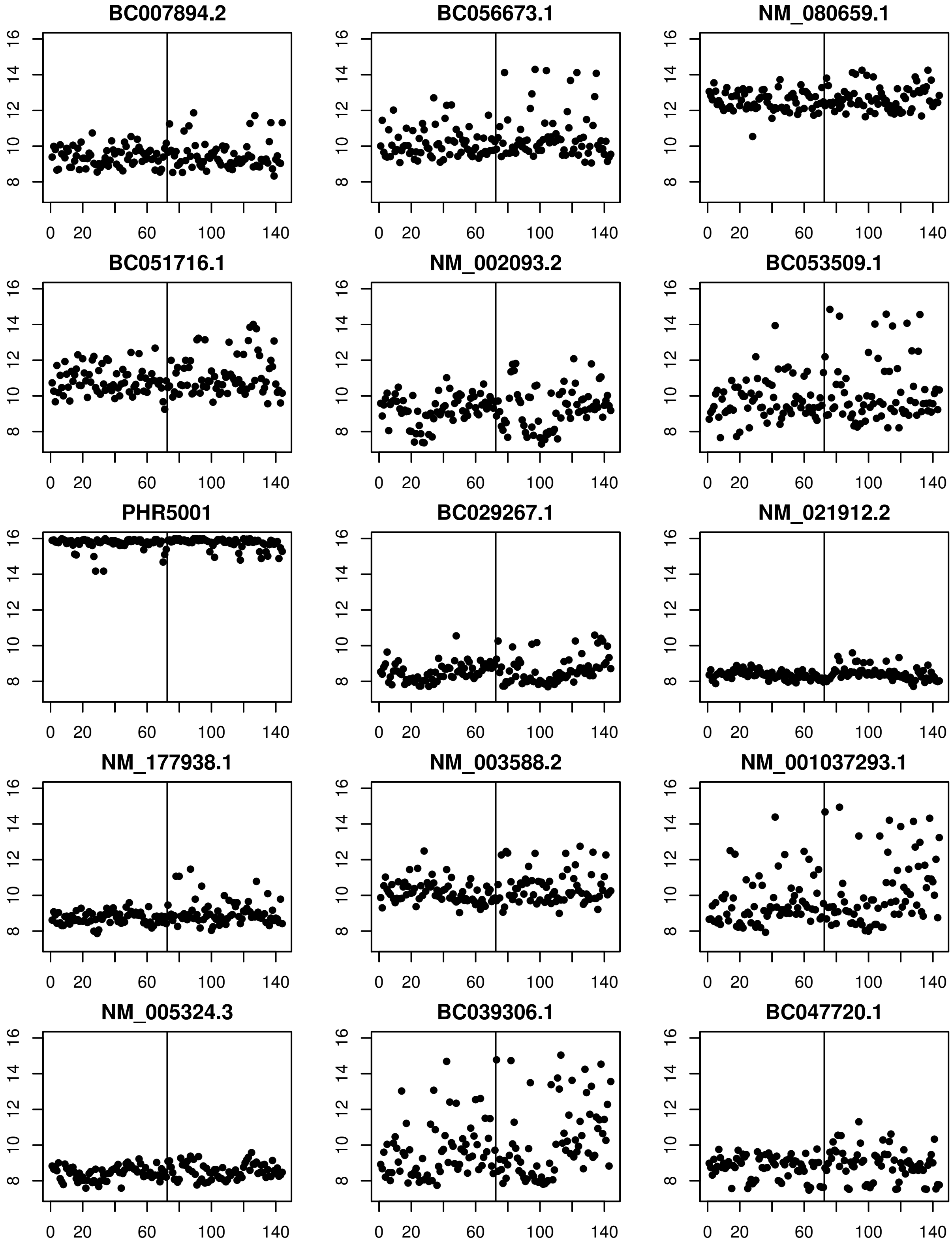

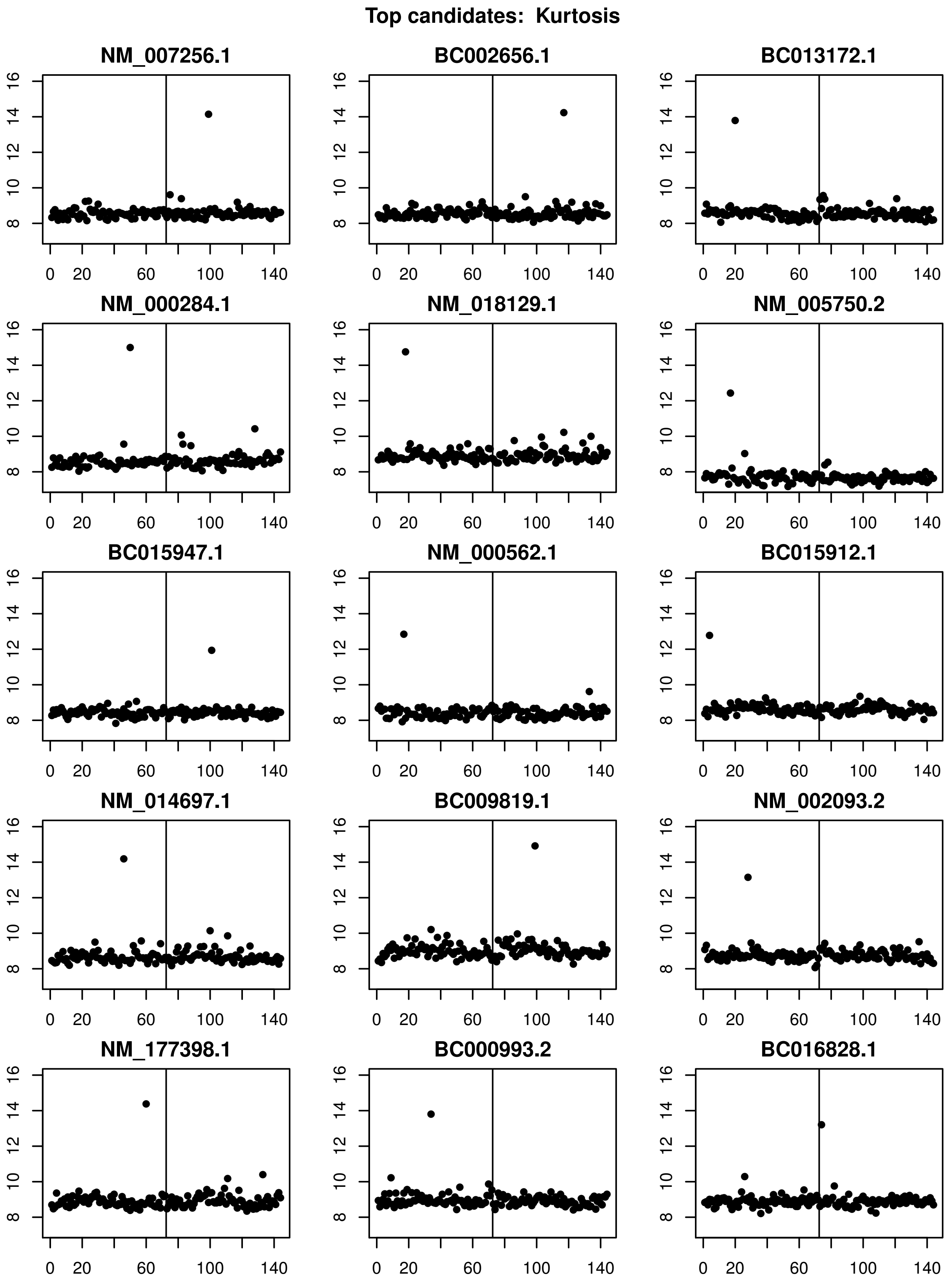
Top candidates: Kurtosis
NM_007256.1
BC002656.1
BC013172.1
NM_000284.1
NM_018129.1
NM_005750.2
BC015947.1
NM_000562.1
BC015912.1
NM_014697.1
BC009819.1
NM_002093.2
NM_177398.1
BC000993.2
BC016828.1
8 10 12 14 16
0 20 60 100 140

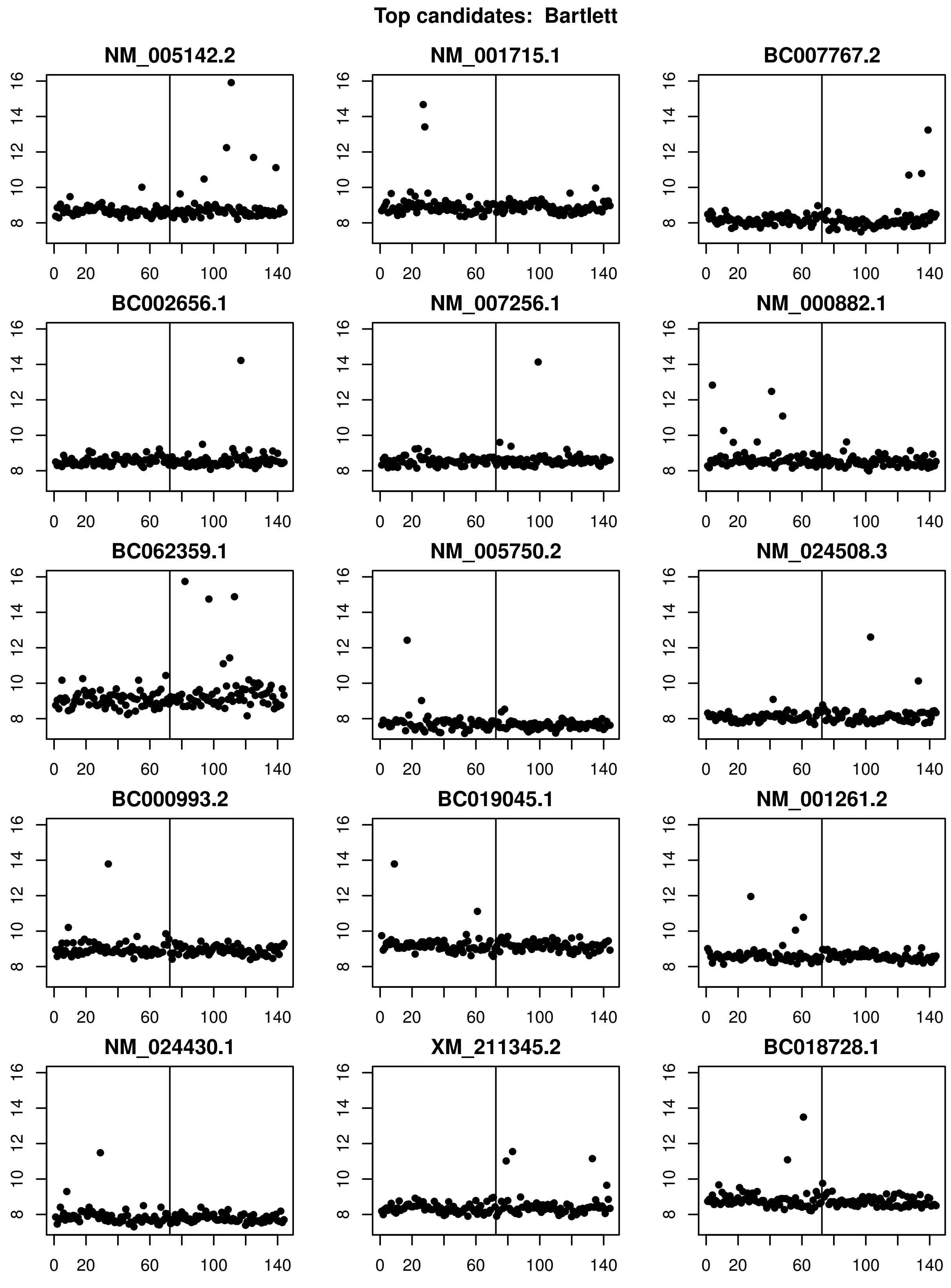

Top candidates: Bartlett
NM_005142.2
NM_001715.1
BC007767.2
BC002656.1
NM_007256.1
NM_000882.1
BC062359.1
NM_005750.2
NM_024508.3
BC000993.2
BC019045.1
NM_001261.2
NM_024430.1
XM_211345.2
BC018728.1

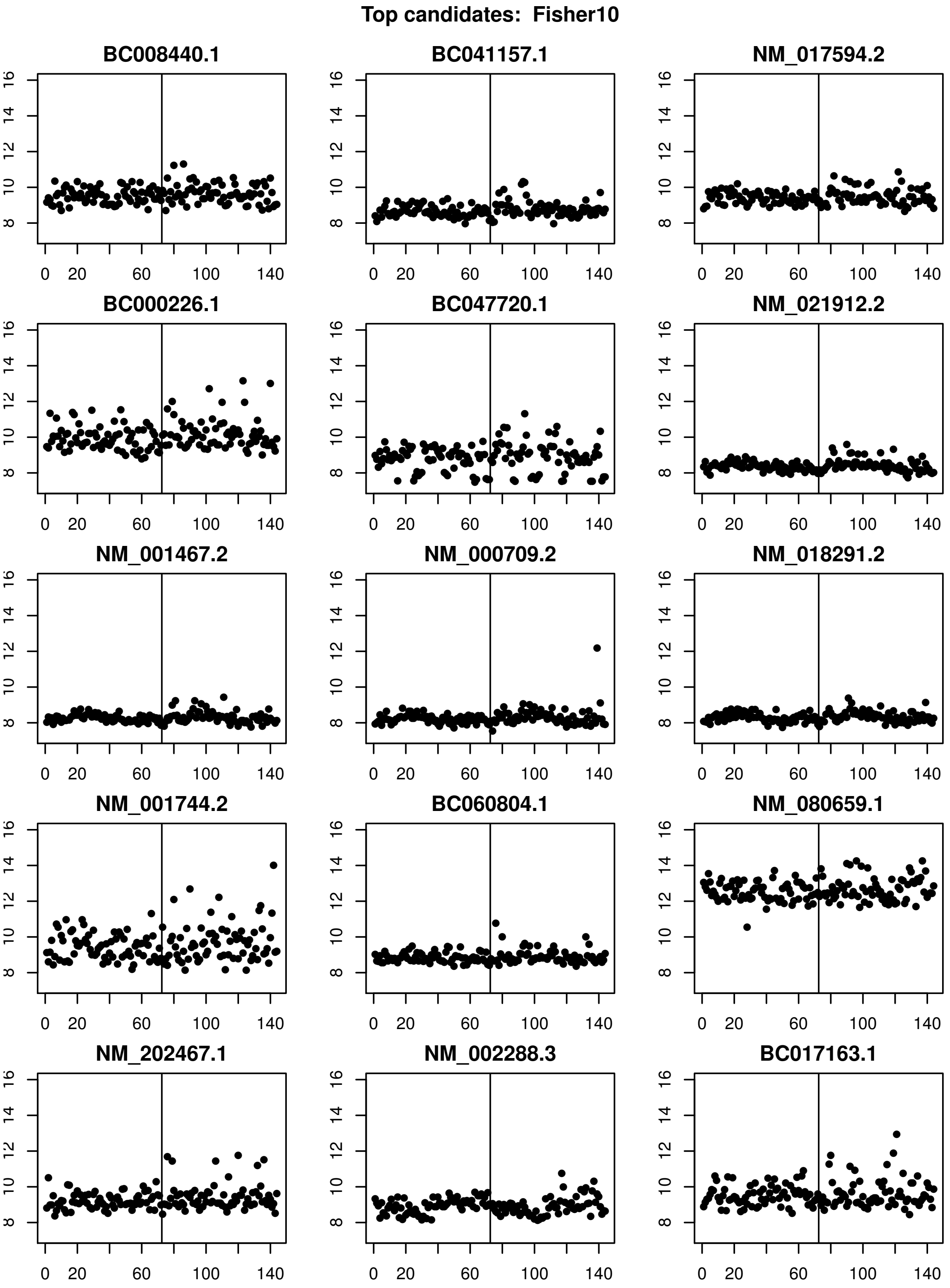

Figure S3. To compare the considered methods, the respective top 15 candidates of each method are shown in (log2-)intensity plots. We used controls and Parkinson patients from the ParkCHIP project.

2 Pseudocode

2.1 FisherSum and Fisher10

Simple version with weights $w_C = w_D = 1$.

```
fishXp.score <- function(control, disease,   q = 0.1,
                 cut.in= 'sg',       # c('sg', 'both')
                 plot=FALSE){
    n.c <- length(control)
    n.d <- length(disease)

    # define cut.off
    if(cut.in=='both'){
        ord  <- round( (n.c + n.d) * q/2)
        cut  <- sort(c(disease, control), decreasing=TRUE)[ord]
        rows <- c(  paste('> pooled[', ord, ']', sep=''),
                    paste('<= pooled[', ord, ']', sep=''))
    }else{
        ord  <- round( n.d * q)
        cut  <- sort(disease, decreasing=TRUE)[ord]
        rows <-   c(  paste('> disease[', ord, ']', sep=''),
                      paste('<= disease[', ord, ']', sep=''))
   }

   # fill in contingency table
 n.sg.d   <-   sum(disease > cut)
   which.up <- which(disease > cut)
   n.sg.c   <-   sum(control > cut)

   tab      <- matrix(c(       n.sg.d,       n.sg.c,
                        n.d - n.sg.d, n.c - n.sg.c ),2,2,
              byrow=TRUE,
dimnames=list(rows, c('disease','control')))
   p        <- fisher.test(tab, alternative= 'greater')$p.value

   # calculate FisherSum
   FS <- 0
   if(n.sg.d > 0){
    location.sg   <- median(disease[which(disease  > cut)])
    location.rest <- median(disease[which(disease <= cut)])

    FS <- (sum( disease[which.up]       - median(control)))-
         (sum( control[control > cut ] - median(control)))
```

```
   }

   # plot results
if(plot==TRUE) { plot(c(control, disease),
      xlab=' control \t \t disease', ylab='',
        main= paste('p Fisher_[', q*100, '%] = ',
     round(p,3), sep='') )
        abline(v=n.c+.5, lty=2)
        abline(h=cut)  }

  return( list(p.value = p, S= FS, table = tab,
          SG.cand = which.up, cutoff= cut   ))
}
```

2.2 PADGE

```
padge <- function(C, D, test= c('wilcox', 't'),
props = c(0.1,.15,.2)
          ){
props <- sort(props, decreasing=TRUE)
test  <- as.character(test)

n.C <- length(C);  n.D <- length(D)
r1  <-  mean(D)/ mean(C)
if(! (1 %in% props)) props <- c(1, props)

ratios <- rel.ratios <- scores <- p.values <- numeric(length(props))

for(i in 1:length(props)){
       q.C    <- quantile(C, 1-props[i])
       q.D    <- quantile(D, 1-props[i])
       subset.C <- C[which(C >= q.C)]
       subset.D <- D[which(D >= q.D)]
       # 'expression ratio'
       ratios[i] <- rn <- mean(subset.D)/ mean(subset.C)
       # 'relative variability of overexpression'
       rel.ratios[i] <- rn/r1

      # calculate p-values
      if(length(subset.C) > 0 & length(subset.D) > 0){
             my.call <- call(paste(test, '.test', sep=''),
                              subset.C , subset.D, alt='less')
                  p <- try(eval(my.call)$p.value, silent=TRUE)
```

```
         p.values[i] <- as.numeric(p)
      }else{
         p.values[i] <-  NA
      }
}
 p.values.adj <- p.adjust(p.values, method='bonferroni')

for(i in 1:length(scores)){
   scores[i] <- -rel.ratios[i]*log(p.values[i])
}
max.score <- max(scores, na.rm=TRUE)

# return(...)
}
```

2.3 ORT

```
ORT <- function(data, col.C=NULL, col.D=NULL, pvalue=TRUE, L=10^4){
     # define observations in control group...
     if(is.null(col.C)){ ifelse(  !(is.null(ncol(data))) ,
          col.C <- 1:(ncol(data)/2),
          col.C <- 1:(length(data)/2) )
     }
     # ... and diseased group
     if( is.null(col.D)){  ifelse( !(is.null(ncol(data))) ,
          col.D  <- (ncol(data)/2) + (1: (ncol(data)/2)  ),
          col.D  <- (length(data)/2) + (1: (length(data)/2)  ) )
      }
    # numbers of samples and variables
    ns <- length(c(col.C , col.D))
    ng <- nrow(data)
    # equation (2.8) in original publication
    expr.med1  <- data[,col.C] - apply(data[,col.C], 1, median) *
                   matrix(1, nrow = ng, ncol = length(col.C))
    expr.med2  <- data[,col.D]   - apply(data[,col.D]  , 1, median)*
    matrix(1, nrow = ng, ncol = length(col.D))
    mads       <- apply(cbind(expr.med1, expr.med2), 1, function(x){
    x <- abs(as.numeric(x)) ;  median(x)  })
    #
    expr.centr <- data -   apply(data[,col.C], 1, median) *
    matrix(1, nrow = ng, ncol = ns)
    expr.std   <- expr.centr/(mads*matrix(1, nrow = ng, ncol = ns))
    #
```

```
    q25 <- apply(data[, col.C], 1, function(x) quantile(x, 0.25))
    q75 <- apply(data[, col.C], 1, function(x) quantile(x, 0.75))
    iqr <- q75 - q25
    # equation (2.9)
    ind.pos <- data[,col.D] > q75 + iqr
    s1      <- rowSums(ind.pos)
    t       <- rowSums(ind.pos * expr.std[,col.D])
    #
    ### simulate p-values
    if(pvalue==TRUE){
    sim.data  <-  matrix(rnorm(ns*L), nrow=L)
    sim.centr <- (sim.data - apply(sim.data[,1:round(ns/2)],
                  1, median)*matrix(1, nrow = L, ncol = ns))
      sim.std   <- sim.centr /  ( apply(sim.data, 1, function(x){
                      x <- as.numeric(x)
                      median( c( abs( x[  1:round(ns/2) ] -
                      median(x[  1:round(ns/2) ] )),
                      abs( x[-(1:round(ns/2))] -
                      median(x[-(1:round(ns/2))] ))  ))
                    })  *  matrix(1, nrow = L, ncol = ns)    )
    sim.q25     <- apply(sim.data, 1, function(x) quantile(x, 0.25))
    sim.q75     <- apply(sim.data, 1, function(x) quantile(x, 0.75))
    sim.iqr     <- sim.q75 - sim.q25
    sim.ind.pos <- sim.data[,-(1:round(ns/2))] > sim.q75 + sim.iqr
    sim.s1      <- rowSums(sim.ind.pos)
    sim.t       <- rowSums(sim.ind.pos * sim.std[,-(1:(ns/2))])
    sim.p       <- as.numeric( length(t) )
    for(i in 1: length(t)) sim.p[i] <- sum(sim.t>t[i])/length(sim.t)
   }
   ####   return()
ret  <-  t
if(pvalue == TRUE & ng == 1){
    ret <-    c(ret,      'p' = sim.p) }
if(pvalue == TRUE & ng > 1){
  ret <- list('t*'=ret, 'p' = sim.p)   }
return(noquote(ret)  )
}
```

2.4 OS

```
OS <- function(data, col.c=NULL, col.d=NULL,
               pvalue=TRUE, L=10^4){
```

```
# default: first half of columns corresponds to controls
#          second half to diseased samples
if(is.null(col.c)) col.c <- 1:(ncol(data)/2)
if(is.null(col.d)) col.d <-   (ncol(data)/2)+(1:(ncol(data)/2))

# calculate numbers of samples and genes
ns <- length(c(col.c , col.d))
ng <- nrow(data)

# equation (2.2) in original paper
exprs.mad <- (data - apply(data, 1, median) *
             matrix(1, nrow = ng, ncol = ns)) /
             (apply(data, 1, mad) * matrix(1, nrow = ng, ncol = ns))
# calculate quantiles
q25 <- apply(data, 1, function(x) quantile(x, 0.25))
q75 <- apply(data, 1, function(x) quantile(x, 0.75))
iqr <- q75 - q25

# identify up-regulated outliers in disease group
ind.pos <- exprs.mad[,col.d] > q75 + iqr

# equation (2.3)
w1       <- rowSums(ind.pos * exprs.mad[,col.d])

# simulate p-values if desired
if(pvalue==TRUE){
    sim.data      <-  matrix(rnorm(ns*L), nrow=L)
    # analogously to real data:
    # sim.exprs.mad, sim.q25, sim.q75, sim.iqr, sim.ind.pos, sim.w1

    # proportion of larger OS values
    sim.p  <- as.numeric( length(w1) )
    for(i in 1: length(w1)){
        sim.p[i]    <- sum( sim.w1 > w1[i])/length(sim.w1)
}
}

# return(...)
}
```
